# Supplementary material for: A mixed-methods study measuring the effectiveness of a menstrual health intervention on menstrual health knowledge, perceptions and practices among young women in Zimbabwe
Source: BMJ Open. 2023 Mar 9;13(3):e067897. doi: 10.1136/bmjopen-2022-067897 (PMC10008401; doi:10.1136/bmjopen-2022-067897)
Supplement: Supplementary data [file bmjopen-2022-067897supp001.pdf]

MH STUDY ADMINISTRATION<br>This section is to be filled out by the attending staff member			
**A00**	*STAFFNO*	Staff Initials	☐☐☐
**A01**	*STUDYNO*	Study Participant Cohort Number	☐☐☐☐☐☐
**A02**	*DOB*	Date of Birth (dd/mm/yyyy)	☐☐/☐☐/☐☐☐☐
**A03**	*AGE*		☐☐
**A04**	*DATE*	Date of visit (dd/mm/yyyy)	☐☐/☐☐/20☐☐
**A05**	*VISIT*	Type of visit	Initial ☐ 6m☐ 12m☐

This questionnaire is to be filled out by participants in the menstrual health study

The purpose of this questionnaire is to collect information about you and how you feel and behave during your period.

There are 9 parts to this questionnaire. It should not take more than 30 minutes of your time. Please read the questions carefully and fill in the questionnaire as honestly as possible.

Thank you for your participation.

PART I: SOCIODEMOGRAPHIC INFORMATION

These questions are to help understand more about your life

**A06**	*EDU*	Highest Level of Education	Please tick your highest level of education:<br>No Formal School Education ☐<br>Incomplete Primary School ☐<br>Complete Primary School ☐<br>Incomplete High School Education ☐<br>Complete High School Education (O'Levels) ☐<br>Complete High School Education (A'Levels) ☐<br>Incomplete Tertiary Education ☐

			Complete University ☐
**A07**	*JOB*	Do you have a paying job?	Yes ☐ No ☐
**A08**	*MONEY*	If you do not work, where do you get your money	I do not get money from anyone ☐<br>I get money from a relative in the household (mother/ father/brother/sister etc.) ☐<br>I get money from a relative outside the household ☐<br>I get money from my partner ☐<br>Other ☐<br>If you chose "Other", please list here: ___________
**A09**	*MSTAT*	Marital Status	Single (Never Married) ☐<br>Married, or living as if you are married ☐<br>Widowed ☐<br>Divorced/Separated ☐
**A10**	*RES*	Residence	Urban ☐<br>Peri-Urban ☐<br>Rural ☐
**A11**	*RELGN*	Religion	Not Religious ☐<br>Traditional ☐<br>Christian (Pentecostal) ☐<br>Christian (Protestant) ☐<br>Roman Catholic ☐<br>Other ☐

			If you chose "Other", please list here: __________
**A12**	*HH*	Please tick any items your household has:	Please tick all that apply:<br>Television ☐<br>Radio ☐<br>Mobile Phone ☐<br>Electricity ☐<br>Water source inside the house ☐<br>Latrine inside the house ☐
**A13**	*INC*	How many people live in your household with you:	None (I live alone) ☐<br>1 – 3 other people ☐<br>4 – 6 other people ☐<br>More than 6 people ☐
**PART II: GENERAL QUESTIONS ABOUT YOURSELF**<br>**These questions are about you and why you came to the CHIEDZA site**			
**A14**	*CHIEDZA 1*	What brought you to CHIEDZA today?	Please tick all that apply:<br>STI Treatment ☐<br>Menstrual Hygiene Management ☐<br>HIV Testing ☐<br>HIV Treatment ☐<br>Condoms ☐<br>Family Planning ☐<br>Counselling ☐

			Other ☐<br><br>If you chose "Other", please list here: ___________
**A15**	*CHIEDZA 2*	Would you come to CHIEDZA if you could not receive MH products and information?	Yes ☐ No ☐
**PART III: PERIOD and PUBERTY KNOWLEDGE**<br>**These questions are about your understanding of puberty and periods**			
**QUESTIONS ABOUT PUBERTY AND MENSTRUATION**			
**A16**	*KNW1*	Changes in the body during puberty happen because of hormones	Yes ☐ No ☐ I don't know ☐
**A17**	*KNW2*	Puberty continues throughout a girl's life	Yes ☐ No ☐ I don't know ☐
**A18**	*KNW2*	Menstruation in girls and women is normal	Yes ☐ No ☐ I don't know ☐
**A19**	*KNW3*	When a girl gets her first menstrual period, her body is able to carry a child	Yes ☐ No ☐ I don't know ☐
**A20**	*KNW4*	Menstrual blood is caused by the breakdown of the lining of the womb	Yes ☐ No ☐ I don't know ☐
**A21**	*KNW5*	It is normal to have irregular periods as a teenager	Yes ☐ No ☐ I don't know ☐
**A22**	*KNW6*	Menstruation continues throughout a girl's life	Yes ☐ No ☐ I don't know ☐
**A23**	*KNW7*	A period normally lasts 2 days or less	Yes ☐ No ☐ I don't know ☐
**A24**	*KNW8*	Period products that are inserted into the vagina	Yes ☐ No ☐ I don't know ☐

		(such as tampons and the menstrual cup) affect your virginity	
**PART IV: QUESTIONS ABOUT YOUR PERIOD HISTORY**<br>**The following questions are about you and your menstrual periods**			
The following questions are about your period history			
**A25**	*MENARCHE*	How old were you when you first started your periods?	Age ☐☐<br>I don't remember ☐
**A26**	*TAUGHT*	Who first told you or taught you about periods	No one told me about periods ☐<br>My mother ☐<br>My father ☐<br>My female/male friend ☐<br>My teacher ☐<br>Other ☐<br>If you chose "Other", please list here: __________
**A27**	*INFO1*	Do you feel you had enough information and education to manage your period when you got your first period?	Yes ☐<br>Somewhat ☐<br>Not Really ☐<br>Definitely No ☐
**A28**	*INFO2*	What did you think was happening when you got your first period?	Curse ☐<br>Disease ☐<br>Injury ☐<br>Normal function for young women ☐<br>I did not know what was happening ☐

			Other ☐<br><br>If you chose "Other", please list here: ___________
**A29**	*DAYS*	On average, how many days do you usually bleed for during your periods?	1 – 3 days ☐<br>4 – 7 days ☐<br>More than 7 days ☐
**A30**	*RCNTDAYS*	How many days did you bleed during your most recent period?	☐☐<br>I Do Not Remember ☐
**PART V: QUESTIONS ABOUT PAIN MANAGEMENT**<br>**The following questions are about menstruation-related pain**			
**A31**	*PAINKNW1*	Painkillers can be used to relieve menstrual pain	Yes ☐ No ☐ I don't know ☐
**A32**	*PAINKNW2*	Painkillers can cause problems having children (barrenness)	Yes ☐ No ☐ I don't know ☐
**A33**	*PAINKNW3*	Exercising, stretching, or putting a warm towel or bottle on your stomach can relieve menstrual pain	Yes ☐ No ☐ I don't know ☐
During my last (most recent) menstrual period…			
**A34**	*PAIN1*	I experienced period-related pain in my lower back and/or abdomen	Yes ☐ No ☐
**A35**	*PAIN2*	I missed work or school due to period-related pain	Yes ☐ No ☐
**PART VI:**<br>**QUESTIONS ABOUT PRACTICES AND PERCEPTIONS DURING MENSTRUATION**<br>**The following questions are about how you manage your menstruation**			
The following questions are about your last (most recent) period			

**A36**	*PRDCT*	During your most recent period, what material(s)did you mostly use to collect or absorb your period blood?	Please tick all that apply:<br>Cloth/Rags/Fabric ☐<br>Reusable Pads ☐<br>Disposable Pads ☐<br>Menstrual Cup ☐<br>Period Pants ☐<br>Other ☐<br>Not Applicable ☐<br>If you chose "Other", please list here: ___________
**A37**	*HPRDCT*	During your most recent period, what material(s)did you mostly use to collect or absorb your period blood when at home?	Please tick all that apply:<br>Cloth/Rags/Fabric ☐<br>Reusable Pads ☐<br>Disposable Pads ☐<br>Menstrual Cup ☐<br>Period Pants ☐<br>Other ☐<br>Not Applicable ☐<br>If you chose "Other", please list here: ___________
**A38**	*SWPRDCT*	During your most recent period, what material(s)did you mostly use to collect or absorb your period blood when at school/work?	Please tick all that apply:<br>Cloth/Rags/Fabric ☐<br>Reusable Pads ☐<br>Disposable Pads ☐<br>Menstrual Cup ☐<br>Period Pants ☐

			Other ☐<br>Not Applicable ☐<br>If you chose "Other", please list here: __________
During my last (most recent) menstrual period…			
**A39**	*PRDCT 1*	My menstrual products were comfortable	Strongly Disagree ☐<br>Disagree ☐<br>Agree ☐<br>Strongly Agree ☐
**A40**	*PRDCT 2*	I had enough of my menstrual products to change them as often as I wanted to	Strongly Disagree ☐<br>Disagree ☐<br>Agree ☐<br>Strongly Agree ☐
**A41**	*PRDCT 3*	Before I used them, I was satisfied with the cleanliness of my menstrual products	Strongly Disagree ☐<br>Disagree ☐<br>Agree ☐<br>Strongly Agree ☐
**A42**	*PRDCT 4*	I could get more of my menstrual materials when I needed to	Strongly Disagree ☐<br>Disagree ☐<br>Agree ☐<br>Strongly Agree ☐
**A43**	*PRDCT 5*	I was worried that my menstrual products would allow blood to pass through my outer garments	Strongly Disagree ☐<br>Disagree ☐<br>Agree ☐<br>Strongly Agree ☐
**A44**	*PRDCT 6*	I was worried that my menstrual products would	Strongly Disagree ☐<br>Disagree ☐

		move from place while I was wearing them	Agree ☐<br>Strongly Agree ☐
**A45**	*PRDCT 7*	I worried about how I would get more of my menstrual material if I ran out	Strongly Disagree ☐<br>Disagree ☐<br>Agree ☐<br>Strongly Agree ☐
**A46**	*PRDCT 8*	I felt comfortable carrying spare menstrual products with me outside my home	Strongly Disagree ☐<br>Disagree ☐<br>Agree ☐<br>Strongly Agree ☐
**A47**	*PRDCT 9*	I felt comfortable carrying menstrual products to the place where I changed them	Strongly Disagree ☐<br>Disagree ☐<br>Agree ☐<br>Strongly Agree ☐
**A48**	*PRDCT 10*	I felt comfortable storing my menstrual products until my next period	Strongly Disagree ☐<br>Disagree ☐<br>Agree ☐<br>Strongly Agree ☐
**A49**	*PRDCT 11*	I was able to wash my hands when I wanted to	Strongly Disagree ☐<br>Disagree ☐<br>Agree ☐<br>Strongly Agree ☐
**If participant ticks "Disposable Pads" or "Cloth/Rags/Fabric"**			
During my last (most recent) period…			
**B1**	*DIS1*	I was able to immediately dispose of my used menstrual material	Strongly Disagree ☐<br>Disagree ☐<br>Agree ☐

			Strongly Agree ☐
**B2**	*DIS2*	I was able to dispose of my used menstrual material in the way I wanted to	Strongly Disagree ☐<br>Disagree ☐<br>Agree ☐<br>Strongly Agree ☐
**B3**	*DIS3*	I worried about where to dispose of my used menstrual materials	Strongly Disagree ☐<br>Disagree ☐<br>Agree ☐<br>Strongly Agree ☐
**B4**	*DIS4*	I was concerned that others would see my menstrual materials in the place I disposed them	Strongly Disagree ☐<br>Disagree ☐<br>Agree ☐<br>Strongly Agree ☐
**If participant ticks "Cloth/Rags/Fabric", "Period Pants" and/or "Reusable Pads":**			
During my last (most recent) period…			
**C1**	*REUS1*	I had enough water to soak or wash my menstrual products	Strongly Disagree ☐<br>Disagree ☐<br>Agree ☐<br>Strongly Agree ☐
**C2**	*REUS2*	I had access to a basin or dish to soak or wash my menstrual products whenever I needed	Strongly Disagree ☐<br>Disagree ☐<br>Agree ☐<br>Strongly Agree ☐
**C3**	*REUS3*	I was able to wash my menstrual products <u>when</u> I wanted to	Strongly Disagree ☐<br>Disagree ☐<br>Agree ☐<br>Strongly Agree ☐

**C4**	*REUS4*	I had enough soap to wash my menstrual products	Strongly Disagree ☐<br>Disagree ☐<br>Agree ☐<br>Strongly Agree ☐
**C5**	*REUS5*	I was able to dry my products when I wanted to	Strongly Disagree ☐<br>Disagree ☐<br>Agree ☐<br>Strongly Agree ☐
**C6**	*REUS6*	I was worried that someone would see me while I was washing my menstrual products	Strongly Disagree ☐<br>Disagree ☐<br>Agree ☐<br>Strongly Agree ☐
**C7**	*REUS7*	I was worried that my menstrual products would not be dry when I needed them	Strongly Disagree ☐<br>Disagree ☐<br>Agree ☐<br>Strongly Agree ☐
**C8**	*REUS8*	I was worried that others would see my menstrual products while they were drying	Strongly Disagree ☐<br>Disagree ☐<br>Agree ☐<br>Strongly Agree ☐
**C9**	*REUS9*	How often did you change your menstrual products during the heaviest day of your most recent period?	Once a day (within a 24hr period) ☐<br>Twice a day (within a 24hr period) ☐<br>Three times a day (within a 24hr period) ☐<br>More than three times a day (within a 24hr period) ☐
**C10**	*REUS10*	At home, where did you most often change your menstrual products during your most recent period?	In a toilet inside the building ☐<br>In a toilet outside the building ☐<br>In a private room ☐<br>Outside (in the field/by the river) ☐

			Other ☐<br>If you chose "Other", please explain: __________
**C11**	*REUS11*	At school/work, where did you most often change your menstrual products during your most recent period?	In a toilet inside the building ☐<br>In a toilet inside the building ☐<br>In a toilet outside the building ☐<br>In a private room ☐<br>Outside (in the field/by the river) ☐<br>Other ☐<br>Not applicable ☐<br>If you chose "Other", please explain: __________
**C12**	*REUS12*	Where did you wash your menstrual products during your last period?	In the bathroom ☐<br>At a private well or tap in the house ☐<br>At a public well or tap outside the house ☐<br>In a pond or river ☐<br>Did not wash your menstrual products ☐<br>Other ☐<br>If you chose "Other", please explain: __________
**C13**	*REUS13*	What did you use to wash your products with during your most recent period?	With water only ☐<br>With soap and water ☐<br>Did not wash my menstrual products ☐<br>Other ☐<br>If you chose "Other", please explain: __________
**C14**	*REUS14*	Where did you dry your menstrual products during your most recent period?	Outside in the sun ☐<br>Outside in the sun, under a cloth ☐<br>Inside in the house in an open space ☐

			Inside the house in a closed space ☐<br>Did not dry my reusable pad, period pants, or cloth/rags/fabric ☐<br>Other ☐<br>If you chose "Other", please explain: ___________
**C15**	*REUS15*	Did you ever wear your menstrual product when it was damp (not yet dry) during your most recent period?	Yes ☐ **(**No ☐<br>Do Not Remember ☐
**C16**	*REUS16*	Where did you store your menstrual products after your most recent period?	In a cupboard ☐<br>In the toilet ☐<br>In an open space ☐<br>In a hidden place ☐<br>Did not store ☐<br>Other ☐<br>If you chose "Other", please explain: ___________
**If participant ticks "Menstrual Cup":**			
During my last (most recent) period…			
**D1**	*MC1*	I was able to sterilize my menstrual cup <u>when</u> I wanted after my period	Strongly Disagree ☐<br>Disagree ☐<br>Agree ☐<br>Strongly Agree ☐
**D2**	*MC2*	I was able to rinse my menstrual cup <u>when</u> I wanted	Strongly Disagree ☐<br>Disagree ☐<br>Agree ☐<br>Strongly Agree ☐

**D3**	*MC3*	I had enough water to rinse my menstrual cup	Strongly Disagree ☐<br>Disagree ☐<br>Agree ☐<br>Strongly Agree ☐
**D4**	*MC4*	I was worried that someone would see me while I was rinsing my menstrual cup	Strongly Disagree ☐<br>Disagree ☐<br>Agree ☐<br>Strongly Agree ☐
**D5**	*MC5*	I was worried that someone would see me while I was sterilizing my menstrual cup	Strongly Disagree ☐<br>Disagree ☐<br>Agree ☐<br>Strongly Agree ☐
**D6**	*MC6*	How long did it take you to get comfortable and relaxed using your menstrual cup?	1 Period ☐<br>2 Periods ☐<br>3 Periods ☐<br>4 or More Periods ☐<br>Still Not Comfortable or Relaxed ☐
**D7**	*MC7*	What is difficult about using your menstrual cup?	Tick All that Apply:<br>Could not get the cup in ☐<br>Too much pain and discomfort ☐<br>Difficult to clean ☐<br>Difficult to remove ☐<br>Other ☐<br>If you chose “Other”, please explain: ___________
**D8**	*MC8*	How often did you empty and re-insert your menstrual cup during the heaviest day of your most recent period?	Once a day (within a 24hr period) ☐<br>Twice a day (within a 24hr period) ☐<br>Three times a day (within a 24hr period) ☐<br>More than three times a day (within a 24hr period) ☐

**D9**	*MC9*	At home, where did you most often empty and re-insert your menstrual cup when you needed to during your most recent period?	In a toilet inside the building ☐<br>In a toilet outside the building ☐<br>Outside (in the field/by the river) ☐<br>Other ☐<br>If you chose "Other", please explain: ___________
**D10**	*MC10*	At school/work, where did you most often empty and re-insert your menstrual cup when you needed to during your most recent period?	In a toilet inside the building ☐<br>In a toilet outside the building ☐<br>Outside (in the field/by the river) ☐<br>Not Applicable ☐<br>Other ☐<br>If you chose "Other", please explain: ___________
**D11**	*MC11*	Where did you rinse your menstrual cup during your last period?	In a private bathroom ☐<br>In a public bathroom ☐<br>At a private well or tap in the house ☐<br>At a public well or tap outside the house ☐<br>In a pond or river ☐<br>Did not rinse my menstrual cup ☐<br>Other ☐<br>If you chose "Other", please explain: ___________
**D12**	*MC12*	What did you rinse your menstrual cup with during your most recent period?	With water only ☐<br>With soap and water ☐<br>Did not rinse my menstrual cup ☐<br>Other ☐<br>If you chose "Other", please explain: ___________

**D13**	*MC13*	During your most recent period, how did you sterilise your menstrual cup?	Boiled cup for 3 min ☐<br>Washed with boiling water ☐<br>Soaked in vinegar and water solution ☐<br>Other ☐<br>If you chose "Other", please explain: ___________
**D14**	*MC14*	Did you store your menstrual cup in an aerated bag between periods?	Yes ☐ No☐
**D15**	*MC15*	During your most recent period, did you ever use a reusable or disposable pad along with the menstrual cup?	Yes☐ No☐
**PART V: QUESTIONS ABOUT YOUR WASHING PRACTICES**<br>**The following questions are about how you wash your body and your vagina during your last (most recent) period**			
**A50**	*WASH1*	What type of washing did you do when you were on your most recent period?	External genital wash only ☐<br>Full body wash ☐<br>I did not wash myself ☐<br>Other☐<br>If you chose "Other", please explain: ___________
**A51**	*WASH2*	On average, how many times did you wash your body during your most recent period?	Less than once a day ☐<br>Once a day ☐<br>Twice a day ☐<br>Three times a day or more ☐
**A52**	*WASH3*	What did you use to wash yourself during your most recent period?	Water only ☐<br>Soap and water ☐<br>Not Applicable ☐<br>Other☐<br>If you chose "Other", please explain: ___________
**PART VII: QUESTIONS ABOUT YOUR ATTITUDES ABOUT MENSTRUATION**<br>**These questions are about how feel about menstruation**			

**A53**	*ATTITUDE1*	I feel dirty or impure during my menstrual period	Strongly Disagree☐<br>Disagree ☐<br>Neutral☐<br>Agree☐<br>Strongly Agree☐
**A54**	*ATTITUDE2*	I feel like I can talk to friends about menstruation	Strongly Disagree☐<br>Disagree ☐<br>Neutral☐<br>Agree☐<br>Strongly Agree☐
**A55**	*ATTITUDE3*	I feel like I can talk to my family members about menstruation	Strongly Disagree☐<br>Disagree ☐<br>Neutral☐<br>Agree☐<br>Strongly Agree☐
**A56**	*ATTITUDE4*	I feel proud that I have my periods	Strongly Disagree☐<br>Disagree ☐<br>Neutral☐<br>Agree☐<br>Strongly Agree☐
**A57**		It is important that I keep my period a secret from everyone	Strongly Disagree☐<br>Disagree ☐<br>Neutral☐<br>Agree☐<br>Strongly Agree☐
**A58**	*ATTITUDE5*	I worry that boys will tease me about my period	Strongly Disagree☐<br>Disagree ☐<br>Neutral☐

			Agree ☐<br>Strongly Agree ☐
**A59**	*ATTITUDE6*	I am anxious about my next period	Strongly Disagree ☐<br>Disagree ☐<br>Neutral ☐<br>Agree ☐<br>Strongly Agree ☐
**PART VIII: PARTICIPATION**<br>**The following questions are about how your period may affect your everyday activities**			
**A60**	*PRTCPTN1*	In the last 3 months, have you missed work/school because of your period?	Yes ☐ No ☐<br>**If "No", skip next question**
**A61**	*PRTCPTN2*	Why did you miss work/school?	Please tick all that apply:<br>Pain ☐<br>Nothing To Use To Manage My Period ☐<br>No place to change or wash ☐<br>Ashamed or embarrassed ☐<br>Scared I will leak ☐<br>Told to go home/Not allowed ☐
**A62**	*PRTCPTN3*	In the last 3 months, have you missed social events or community/religious happenings because of your period?	Yes ☐ No ☐<br>**If "No", skip next question**
**A63**	*PRTCPTN4*	Why did you miss the social events or social/religious happenings?	Please tick all that apply:<br>Pain ☐<br>Nothing To Use To Manage My Period ☐<br>No place to change or wash ☐<br>Ashamed or embarrassed ☐

			Scared I will leak☐<br>Told to go home/Not allowed☐
**A64**	*PRTCPT N5*	In the last 3 months, what everyday activities have you missed out on because of your period?	Please tick all that apply:<br>Household activities (washing clothes, dishes, etc)☐<br>Sports (athletics, running, swimming, etc) ☐<br>Income-generating activities☐<br>Religious activities☐<br>Socialising with friends and family☐<br>School☐
**PART IX: FACTORS AT HOME**<br>**The following questions are about the surroundings in which you manage your period at home**			
At home during my last (most recent) period…			
**A65**	*FHOME 1*	I was able to change my menstrual products <u>when</u> I wanted to	Strongly Disagree☐<br>Disagree ☐<br>Agree☐<br>Strongly Agree☐
**A66**	*FHOME 2*	I was satisfied with the place I used to change my menstrual products	Strongly Disagree☐<br>Disagree ☐<br>Agree☐<br>Strongly Agree☐
**A67**	*FHOME 3*	I had a clean place to change my menstrual products	Strongly Disagree☐<br>Disagree ☐<br>Agree☐<br>Strongly Agree☐
**A68**	*FHOME 4*	I was worried I would not be able to change my menstrual products <u>when</u> I needed to	Strongly Disagree☐<br>Disagree ☐<br>Agree☐<br>Strongly Agree☐
**A69**	*FHOME 5*	I was worried someone would	Strongly Disagree☐

		see me while I was changing my menstrual products	Disagree ☐<br>Agree ☐<br>Strongly Agree ☐
**A70**	*FHOME 6*	I was worried someone would harm me while I was changing my menstrual products	Strongly Disagree ☐<br>Disagree ☐<br>Agree ☐<br>Strongly Agree ☐
**A71**	*FHOME 7*	I was worried something else would harm me while I was changing my menstrual products	Strongly Disagree ☐<br>Disagree ☐<br>Agree ☐<br>Strongly Agree ☐
**A72**	*FHOME 8*	Do you have access to a clean water source at your home	Yes ☐ No ☐<br>**If "No", skip next question**
**A73**	*FHOME 9*	Where is your clean water source located?	Please tick all that apply:<br>Inside the toilet ☐<br>Outside the toilet but inside the home ☐<br>Outside the house but inside the yard of my home ☐<br>In a public space ☐
**A74**	*FHOME 10*	Do you have access to a toilet at home	Yes ☐ No ☐<br>**If "No", skip next question**
**A75**	*FHOME 11*	Where is your toilet located?	Inside the house ☐<br>Outside the house but inside the yard ☐<br>In a public space ☐
**A76**	*FHOM12*	Does your toilet have a door and a working lock on the inside (for privacy)?	Yes ☐ No ☐
**PART IX: FACTORS AT SCHOOL/WORK**<br>**The following questions are about how you manage your period at school/work**			

At school/work during my last period…			
**A77**	*FSW1*	I was able to change my menstrual products when I wanted to	Strongly Disagree ☐<br>Disagree ☐<br>Agree ☐<br>Strongly Agree ☐<br>Not Applicable ☐
**A78**	*FSW2*	I was satisfied with the place I used to change my menstrual products	Strongly Disagree ☐<br>Disagree ☐<br>Agree ☐<br>Strongly Agree ☐<br>Not Applicable ☐
**A79**	*FSW3*	I had a clean place to change my menstrual materials	Strongly Disagree ☐<br>Disagree ☐<br>Agree ☐<br>Strongly Agree ☐<br>Not Applicable ☐
**A80**	*FSW4*	I was worried I would not be able to change my menstrual material when I needed to	Strongly Disagree ☐<br>Disagree ☐<br>Agree ☐<br>Strongly Agree ☐<br>Not Applicable ☐
**A81**	*FSW5*	I was worried someone would see me while I was changing my menstrual products	Strongly Disagree ☐<br>Disagree ☐<br>Agree ☐<br>Strongly Agree ☐<br>Not Applicable ☐
**A82**	*FSW6*	I was worried someone would	Strongly Disagree ☐

		harm me while I was changing my menstrual products	Disagree ☐<br>Agree ☐<br>Strongly Agree ☐<br>Not Applicable ☐
**A83**	*FSW7*	I was worried something else would harm me while I was changing my menstrual products	Strongly Disagree ☐<br>Disagree ☐<br>Agree ☐<br>Strongly Agree ☐<br>Not Applicable ☐
**A84**	*FSW8*	Do you have access to a toilet at school/work?	Yes ☐ No ☐<br>Not Applicable ☐<br>**If "No" or "Not Applicable", skip next 2 questions**
**A85**	*FSW9*	Where is your toilet located?	Inside the building ☐<br>Outside the building but inside the school/work yard ☐<br>In a public area, outside the school/work yard ☐
**A88**	*FSW12*	Does your toilet have a door and a working lock on the inside (for privacy)?	Yes ☐ No ☐
**A86**	*FSW10*	Do you have access to water at school/work?	Yes ☐ No ☐<br>Not Applicable ☐<br>**If "No" or "Not Applicable", skip next question**
**A87**	*FSW11*	Where is your water source located?	Please tick all that apply:<br>Inside the toilet ☐<br>Outside the toilet but inside the school/work building ☐<br>Outside the building but inside the school/work yard ☐<br>In a public area, outside the school/work yard ☐

THANK YOU FOR YOUR TIME AND YOUR ANSWERS!
