# Supplementary material for: A mixed-methods study measuring the effectiveness of a menstrual health intervention on menstrual health knowledge, perceptions and practices among young women in Zimbabwe
Source: BMJ Open. 2023 Mar 9;13(3):e067897. doi: 10.1136/bmjopen-2022-067897 (PMC10008401; doi:10.1136/bmjopen-2022-067897)
Supplement: Author's manuscript [file bmjopen-2022-067897.draft_revisions.pdf]

BMJ Open

BMJ Open is committed to open peer review. As part of this commitment we make the peer review history of every article we publish publicly available.

When an article is published we post the peer reviewers' comments and the authors' responses online. We also post the versions of the paper that were used during peer review. These are the versions that the peer review comments apply to.

The versions of the paper that follow are the versions that were submitted during the peer review process. They are not the versions of record or the final published versions. They should not be cited or distributed as the published version of this manuscript.

BMJ Open is an open access journal and the full, final, typeset and author-corrected version of record of the manuscript is available on our site with no access controls, subscription charges or pay-per-view fees (http://bmjopen.bmj.com).

If you have any questions on BMJ Open's open peer review process please email info.bmjopen@bmj.com

BMJ Open

Menstrual health intervention improves menstrual health knowledge, perceptions, and practices among young women in Zimbabwe

Journal:	*BMJ Open*
Manuscript ID	bmjopen-2022-067897
Article Type:	Original research
Date Submitted by the Author:	31-Aug-2022
Complete List of Authors:	Tembo, Mandikudza ; London School of Hygiene and Tropical Medicine Faculty of Public Health and Policy, Department of Global Health and Development; Biomedical Research and Training Institute<br>Weiss, Helen; London School of Hygiene and Tropical Medicine Faculty of Epidemiology and Population Health<br>Larsson, Leyla; Biomedical Research and Training Institute,<br>Bandason , Tsitsi; Biomedical Research and Training Institute<br>Redzo, Nicol; Biomedical Research and Training Institute<br>Dauya, E; Biomedical Research and Training Institute<br>Nzanza, Tafadzwa; Biomedical Research and Training Institute<br>Ishumael, Pauline; Biomedical Research and Training Institute<br>Gweshe, Nancy; Biomedical Research and Training Institute<br>Ndlovu, Precious; Biomedical Research and Training Institute<br>Dziva Chikwari, Chido; London School of Hygiene & Tropical Medicine<br>Mavodza, Constancia ; Biomedical Research and Training Institute; London School of Hygiene & Tropical Medicine, Department of Public Health, Environments and Society<br>Renju, Jenny; London School of Hygiene & Tropical Medicine<br>Francis, Suzanna ; London School of Hygiene & Tropical Medicine<br>Ferrand, Rashida; Biomedical Research and Training Institute; London School of Hygiene and Tropical Medicine Department of Clinical Research, Clinical Research Department<br>Mackworth-Young, Constance; Biomedical Research and Training Institute; London School of Hygiene and Tropical Medicine Department of Global Health and Development, Department of Global Health and Development
Keywords:	PUBLIC HEALTH, Public health < INFECTIOUS DISEASES, QUALITATIVE RESEARCH

SCHOLARONE™
Manuscripts

I, the Submitting Author has the right to grant and does grant on behalf of all authors of the Work (as defined in the below author licence), an exclusive licence and/or a non-exclusive licence for contributions from authors who are: i) UK Crown employees; ii) where BMJ has agreed a CC-BY licence shall apply, and/or iii) in accordance with the terms applicable for US Federal Government officers or employees acting as part of their official duties; on a worldwide, perpetual, irrevocable, royalty-free basis to BMJ Publishing Group Ltd ("BMJ") its licensees and where the relevant Journal is co-owned by BMJ to the co-owners of the Journal, to publish the Work in this journal and any other BMJ products and to exploit all rights, as set out in our licence.

The Submitting Author accepts and understands that any supply made under these terms is made by BMJ to the Submitting Author unless you are acting as an employee on behalf of your employer or a postgraduate student of an affiliated institution which is paying any applicable article publishing charge ("APC") for Open Access articles. Where the Submitting Author wishes to make the Work available on an Open Access basis (and intends to pay the relevant APC), the terms of reuse of such Open Access shall be governed by a Creative Commons licence – details of these licences and which Creative Commons licence will apply to this Work are set out in our licence referred to above.

Other than as permitted in any relevant BMJ Author's Self Archiving Policies, I confirm this Work has not been accepted for publication elsewhere, is not being considered for publication elsewhere and does not duplicate material already published. I confirm all authors consent to publication of this Work and authorise the granting of this licence.

For peer review only

Title:

Menstrual health intervention improves menstrual health knowledge, perceptions, and practices among young women in Zimbabwe

Mandikudza Tembo[1,2,5], Helen A Weiss[1], Leyla Larsson[2], Tsitsi Bandason[2], Nicol Redzo[2], Ethel Dauya[2], Tafadzwa Nzanza[2], Pauline Ishumael[2], Nancy Gweshe[2], Precious Ndlovu[2], Chido Dziva Chikwari[1,2], Constancia Vimbayi Mavodza[2,4], Jenny Renju[1], Suzanna C. Francis[1], Rashida A.Ferrand[2,3], Constance R.S. Mackworth-Young[2,5]

Affiliations:

1. *MRC International Statistics & Epidemiology Group, London School of Hygiene and Tropical Medicine, United Kingdom*
2. *Biomedical Research and Training Institute, Harare, Zimbabwe*
3. *Clinical Research Department, London School of Hygiene and Tropical Medicine, United Kingdom*
4. *Department of Public Health, Environments and Society, Faculty of Public Health and Policy, London School of Hygiene and Tropical Medicine, London, United Kingdom*
5. *Department of Global Health and Development, Faculty of Public Health and Policy, London School of Hygiene and Tropical Medicine, London, United Kingdom*

Email addresses of authors:

Helen Weiss: Helen.Weiss@lshtm.ac.uk

Leyla Larsson: llarsson3@outlook.com

Tsitsi Bandason: tbandason@brti.co.zw

Nicol Redzo: nredzo@brti.co.zw

For peer review only - http://bmjopen.bmj.com/site/about/guidelines.xhtml

Ethel Dauya: edauya@brti.co.zw

Tafadzwa Nzanza: nzanzatafadzwa@gmail.com

Pauline Ishumael: chidoishy@gmail.com

Nancy Gweshe: nuncii@gmail.com

Precious Ndlovu: meloosie@gmail.com

Chido Dziva Chikwari: Chido.DzivaChikwari@lshtm.ac.uk

Constancia Mavodza: Constancia-Vimbayi.Mavodza@lshtm.ac.uk

Jenny Renju: Jenny.Renju@lshtm.ac.uk

Suzanna Francis: Suzanna.Francis@lshtm.ac.uk

Rashida Ferrand: Rashida.Ferrand@lshtm.ac.uk

Constance Mackworth-Young: constance.mackworth-young1@lshtm.ac.uk

Keywords:

Menstrual health, knowledge, effectiveness, adolescent health, menstruation, practices

§ Corresponding Author:

Mandikudza M P Tembo

London School of Hygiene and Tropical Medicine,

Phone: +263772 676 417

Email: mandikudza.tembo@lshtm.ac.uk

ORCID: 0000-0002-4520-3317

For peer review only

Abstract

Objectives: While integral to women's physical and mental wellbeing, achieving good menstrual health (MH) remains a challenge for many women. This study investigated the effectiveness of a comprehensive MH intervention on menstrual knowledge, perceptions, and practices among women aged 16-24 years in Harare, Zimbabwe.

Design: Prospective cohort study with pre-post evaluation of an MH intervention.

Setting: Two intervention clusters in Harare, Zimbabwe.

Participants: Overall, 303 female participants were recruited, of whom 189 (62.4%) were seen at midline (median follow-up 7.0; IQR:5.8–7.7 months) and 184 (60.7%) were seen at endline (median follow-up 12.4; IQR:11.9-13.8 months). Cohort follow-up was greatly affected by COVID-19 pandemic and associated restrictions.

Intervention: The MH intervention provided MH education and support, analgesics, and a choice of menstrual products in a community-based setting to improve MH outcomes among young women in Zimbabwe

Primary and secondary outcomes: Effectiveness of a comprehensive MH intervention on improving MH knowledge, perceptions, and practices among young women over time. Quantitative questionnaire data was collected at baseline, midline, and endline. At endline, thematic analysis of four focus group discussions was used to further explore participants' menstrual product use and experiences of the intervention.

Results: At midline, more participants had correct/positive responses for MH knowledge (adjusted odds ratio (aOR)=12.14; 95%CI:6.8-21.8), perceptions (aOR=2.85; 95%CI:1.6-5.1), and practices for reusable pads (aOR=4.68; 95%CI:2.3-9.6) than at baseline. Results were similar comparing endline with baseline for all MH outcomes. Qualitative findings showed that sociocultural norms, stigma, and taboos around

For peer review only - http://bmjopen.bmj.com/site/about/guidelines.xhtml

menstruation, and environmental factors such as limited access to water, sanitation, and hygiene facilities affected the effect of the intervention on MH outcomes.

Conclusions: The intervention improved menstrual health knowledge, perceptions, and practices among young women in Zimbabwe, and the comprehensive nature of the intervention was key to this. MH interventions should address interpersonal, environmental, and societal factors.

Trial registration

Registry: Clinicaltrials.gov

Registration Number: NCT03719521

Registration Date: October 25, 2018

For peer review only

For peer review only - http://bmjopen.bmj.com/site/about/guidelines.xhtml

Strengths and limitations of the study

- This is one of the first menstrual health studies to assess the effectiveness of a multicomponent menstrual health intervention that provided both "hardware", through a choice of menstrual products and analgesics, and "software", through menstrual health education and support, for young women in a community-based setting
- The study used a validated measuring tool (MPNS-36) that has been used in other low- to middle-income countries to comprehensively assess the effect of the menstrual health intervention on key menstrual health outcomes, adding to a key evidence gap in robust quantitative research in menstrual health outcome measures
- The study used a mixed-methods approach that used both qualitative and quantitative analysis to comprehensively assess effects of the intervention on menstrual health outcomes over time, adding to the research supporting the implementation of comprehensive evidence-based menstrual health interventions to improve poor menstrual health indicators across the globe
- Qualitative data may have been prone to social desirability bias due to the sensitive nature of the discussions around menstruation, and recall bias due to being collected retrospectively
- The conclusions of this study may be limited due to the lack of a control group, therefore other unmeasured environmental or external factors, such as natural growth and maturity, may have influenced the observed outcomes

For peer review only - http://bmjopen.bmj.com/site/about/guidelines.xhtml

Introduction

Menstrual health (MH) encompasses a state of physical and psychosocial wellbeing that is informed by access to MH knowledge and products and supportive environment (1-4). MH is integral to women's health and overall wellbeing (5) but MH remains a challenge for many women, especially those in low- and middle-income countries (LMICs) (6, 7). Faced with the stigma and taboo surrounding menstruation, the lack of access to affordable menstrual products and inadequate quality MH education and support, MH remains a critically unaddressed issue (8-12). Many girls and women experience shame, anxiety, and/or untreated pain during their menstruation as they are forced to use ineffective or unhygienic alternatives such as old cloth or poor-quality disposable pads to manage their menstruation. In some instances, this lack of access to MH products and/or education on how to use menstrual products properly can lead to harmful reproductive tract infections or dangerous practices such as transactional sex in effort to access better menstrual products (13, 14).

Acceptable and effective MH interventions are integral to addressing and improving women's health outcomes (3, 15). Informed by a growing understanding of the challenges that women face in managing menstruation, interventions have been implemented in different contexts, especially in LMICs (7, 16). A systematic review of MH interventions and their effect on health outcomes among women in LMICs cautiously reported that MH interventions can inform some positive changes in MH-related outcomes such as MH knowledge levels and menstrual attitudes (7). The review highlighted several gaps and weaknesses in MH intervention research. Many MH intervention studies tend to focus on school-going girls (17) and exclusively address either access to MH "hardware" such as menstrual products and water, sanitation, and hygiene (WASH) facilities in which to manage menstruation, or access to MH "software" such as MH education and support targeted at demystifying and destigmatizing MH-related issues, but rarely both (7, 16, 18). Moreover, without validated and standardized tools and measures for MH

For peer review only - http://bmjopen.bmj.com/site/about/guidelines.xhtml

outcomes more broadly, most studies lack robust evaluations, making it difficult to reliably assess the effect of interventions on MH-related outcomes (7, 19).

Evidence-based MH interventions that address access to menstrual products, pain management, and the larger systematic factors that inform menstrual experiences are needed in Zimbabwe, as elsewhere (20). In 2019, 21.8% of women aged 15–19 years and 18.8% of women aged 20–24 years in Zimbabwe reported not participating in social activities, school, or work in the last 12 months due to menstruation (21). Many of these women face challenges in accessing safe and clean WASH facilities to attend to manage their menstruation at home, school, or work. Some are unable to participate in social activities or concentrate on their work or duties due to unaddressed menstrual pain or discomfort and fear of leaking in public as they are forced to use ineffective old rags, cotton wool, or tissue paper to manage their menstruation (22, 23).

The aim of this study was to address this evidence gap by investigating the effectiveness of a comprehensive MH intervention integrated within a community-based sexual and reproductive health (SRH) service on MH knowledge, perceptions, and practices among young women in Zimbabwe.

Methods

Study design and setting

We conducted a prospective cohort study from December 2019 to August 2021, using quantitative and qualitive methods to assess MH outcomes. The study was nested within a cluster randomised trial of community-based integrated HIV and SRH intervention for young people aged 16-24 years (CHIEDZA trial registration number in clinical trials.gov: NCT03719521) (24). CHIEDZA provided a package of free-of-charge SRH services that included HIV testing and treatment, condoms, risk reduction counselling, contraception and testing and treatment of sexually transmitted infections and an MH intervention (described below) in a youth-friendly environment. CHIEDZA was delivered in 12 intervention clusters in

For peer review only - http://bmjopen.bmj.com/site/about/guidelines.xhtml

three provinces across Zimbabwe (Harare, Bulawayo, and Mashonaland East) from April 2019 to March 2022. Within each province, the CHIEDZA services were delivered by a team of trained healthcare providers comprising two youth workers, one counsellor, two nurses, and four community health workers (CHWs). Participants in the cohort study were followed up every three months for a year.

At baseline and six-month and 12-month visits, participants completed a quantitative questionnaire. At the three-month visit, participants handed-in their completed period tracking diaries and at nine-months, participants were given another period-tracking diary to be completed and handed in at the 12-month visit.

The menstrual health intervention

The MH intervention was preceded by a pilot (from April-July 2019) in all four Harare intervention clusters (20). Results from the pilot were used to refine and inform the scale-up of the intervention across all 12 CHIEDZA intervention clusters. The final MH intervention available to all female CHIEDZA clients included comprehensive MH education and support, provision of a period-tracking diary, two pairs of normal underwear, a choice between either reusable pads (AFRIpads that can be used for up to two years) (www.afripads.com) or the menstrual cup (the Butterfly Cup that can used for up to ten years) (www.vivalily.com), as well as soap, pain management advice and pain medication (a choice between paracetamol or ibuprofen).

Cohort participants

A subset of CHIEDZA female clients who chose to take up any component of the MH intervention in two of the Harare intervention clusters (Budiriro and Hatcliffe) were recruited to the prospective cohort study by two research assistants (RAs) (NG and PN) between December 2019 to February 2020. Equal numbers were sought in two age-strata (16-19 and 20-24 years old) until a cohort size of 318 was reached. Clients were excluded if 1) they were currently pregnant or intended to get pregnant within the next year; 2) they

For peer review only - http://bmjopen.bmj.com/site/about/guidelines.xhtml

experienced irregular or absent bleeding within the last 3 months; or 3) had any condition that precluded informed consent or made study participation unsafe.

Those who consented to participate in the cohort study received two period tracking diaries and three different types of reusable menstrual products including: 1) reusable pads; 2) a menstrual cup; and 3) three pairs of period pants (VivaLily period pants that can be used for up to two years).

Overall, the MH intervention was from April to July 2019, scale-up of MH intervention across all 12 CHIEDZA intervention clusters commenced in July 2019, and recruitment of a subset of CHIEDZA clients for the MH cohort began in December 2019.

Sample size and COVID-19 disruptions

The planned sample size of 300 cohort participants provided 90% power to detect an increase in positive MH outcomes from 10% at baseline to 20% at six-month visit or 12-month visit. This calculation was based on results from a pre-post study of an MH intervention among school-girls in Uganda (25).

COVID-19 and Zimbabwe's response strategies impacted the overall CHIEDZA trial (26, 27). Unable to go to school or work and sustain their livelihoods, many people either sent their children to, or migrated to, the rural areas in search of a more affordable environment (28). Such migration had implications on cohort follow-up and overall exposure to the MH intervention.

Quantitative data collection and analysis

A quantitative questionnaire informed by findings from the pilot intervention (20) and Hennegan's Menstrual Practice Needs Scale (MPNS-36) (19), was used to collect data on: 1) sociodemographic information; 2) menstruation and puberty knowledge; 3) menstrual history; 4) pain management; 5) practices and perceptions during menstruation; 6) washing practices; and 7) attitudes about menstruation. All questions were translated into Shona and back-translated into English with input from MT, NG, and PN. The survey was piloted with a subset of 30 CHIEDZA female clients that took up the MH

For peer review only - http://bmjopen.bmj.com/site/about/guidelines.xhtml

intervention and iteratively revised prior to cohort recruitment (29). The full questionnaire can be found in the supplementary materials.

The self-administered questionnaire was completed at baseline, six-month visit, and 12-month visit. To maximise participation, we reminded participants of upcoming visits via phone. We contacted participants up to three times to reschedule if participants didn't attend their scheduled visit, and offered a home visit before considering the participant lost to follow-up (LTFU) if they did not attend within four weeks of their scheduled visit. If participants later visited CHIEDZA again, we opportunistically invited participants to complete the questionnaire.

For this paper, relevant questions were selected from the questionnaire prior to analysis to assess the effect of the intervention on MH knowledge, perceptions, and practices (for reusable pads and for the menstrual cup) over time (Table 1). The data were analysed using Stata version 16.1 (StatCorp, Texas, USA). Descriptive statistics were used to describe participants' self-reported sociodemographic characteristics, including age, highest level of education, religion, marital status, employment, school, source of income, household income, and household population at baseline, six-month visit, and 12-month visit. We investigated whether LTFU might potentially bias findings by comparing the sociodemographic characteristics of those present at each time-point.

Table 1 Questionnaire questions selected to measure menstrual health outcomes in the study

MH Outcome Measures	Questions	Binary Scale	
MH knowledge	Changes in the body during puberty happen because of hormones	0=No/Don't know	1=Yes
	Puberty continues throughout a girl's life	1=No	0=Yes/Don't know
	Menstruation in girls and women is normal	0=No/Don't know	1=Yes
	When a girl gets her first menstrual period, her body can carry a child	0=No/Don't know	1=Yes

For peer review only

	Menstrual blood is caused by the breakdown of the lining of the womb	0=No/Don't know	1=Yes
	It is normal to have irregular periods as a teenager	0=No/Don't know	1=Yes
	Menstruation continues throughout a girl's life	1=No/Don't know	0=Yes
	A period normally lasts 2 days or less	1=No/Don't know	0=Yes
	Period products that are inserted into the vagina (such as tampons and the menstrual cup) affect your virginity	1=No/Don't know	0=Yes
	**Highest possible score for MH knowledge**	**9**	
**MH perceptions**	I feel dirty or impure during my menstrual period	0=Neutral/Agree/Strongly Agree	1=Disagree/Strongly Disagree
	I feel like I can talk to friends about menstruation	1=Neutral/Agree/Strongly Agree	0=Disagree/Strongly Disagree
	I feel like I can talk to my family members about menstruation	1=Agree/Strongly Agree	0=Neutral/Disagree/Strongly Disagree
	It is important that I keep my period secret for everyone	0=Neutral/Agree/Strongly Agree	1=Disagree/Strongly Disagree
	I worry that boys will tease me about my period	0=Neutral/Agree/Strongly Agree	1=Disagree/Strongly Disagree
	I am anxious about my next period	0=Neutral/Agree/Strongly Agree	1=Disagree/Strongly Disagree
	**Highest possible score for MH perceptions**	**6**	
**MH practices (reusable pads)**	I had enough water to soak or wash my menstrual products	1=Agree/Strongly Agree	0=Neutral/Disagree/Strongly Disagree
	I was able to wash my menstrual products when I wanted to	1=Agree/Strongly Agree	0=Neutral/Disagree/Strongly Disagree
	I had enough soap to wash my menstrual products when I wanted to	1=Agree/Strongly Agree	0=Neutral/Disagree/Strongly Disagree
	I was able to dry my products when I wanted to	1=Agree/Strongly Agree	0=Neutral/Disagree/Strongly Disagree

For peer review only - http://bmjopen.bmj.com/site/about/guidelines.xhtml

	I was worried that someone would see me while I was washing my menstrual product	0=Neutral/ Agree/Strongly Agree	1=Disagree/Strongly Disagree
	I was worried that my menstrual products would not dry when I needed them	0=Neutral/ Agree/Strongly Agree	1=Disagree/Strongly Disagree
	I was worried that others would see my menstrual products while they were drying	0=Neutral/ Agree/Strongly Agree	1=Disagree/Strongly Disagree
	**Highest possible score for MH practices (reusable pads)**	**7**	
**MH practices (menstrual cups)**	I was able to sterilize my menstrual cup when I wanted after my period	1=Agree/Strongly Agree	0=Neutral/Disagree/Strongly Disagree
	I was able to rinse my menstrual cup when I wanted	1=Agree/Strongly Agree	0=Neutral/Disagree/Strongly Disagree
	I had enough water to rinse my menstrual cup	1=Agree/Strongly Agree	0=Neutral/Disagree/Strongly Disagree
	I was worried that someone would see me while I was rinsing my menstrual cup	0=Neutral/ Agree/Strongly Agree	1=Disagree/Strongly Disagree
	I was worried that someone would see me while I was sterilizing my menstrual cup	0=Neutral/ Agree/Strongly Agree	1=Disagree/Strongly Disagree
	**Highest possible score for MH practices (menstrual cups)**	**5**	

At each time-point, and for each of the MH-related outcomes: MH knowledge, perceptions, practices for reusable pad use, and practices for menstrual cup use, we calculated the average score of relevant items and the proportion of participants who answered all relevant items either "correctly" or "positively". We used repeated measures logistic regression to assess the pre-post change in the proportion of participants with correct/positive outcomes from baseline to six-moth visit, and baseline to 12-month visit respectively. Similarly, we used repeated measures linear regression to assess the pre-post change in scores for continuous variables from baseline to six-month visit, and baseline to 12-month respectively. We investigated potential confounders by assessing the association between each sociodemographic variable with exposure (visit time-point) and each binary MH outcome measure. The variable was

For peer review only

For peer review only - http://bmjopen.bmj.com/site/about/guidelines.xhtml

considered a potential confounder if association with both the outcome and exposure had a $p<0.05$. If the crude odds ratio or regression coefficient changed by more than 10% on adjusting for the potential confounder, the variable was considered a confounder and was retained in the model.

Qualitative data collection and analysis

Following the 12-month visits, after exposure to the intervention, four focus group discussions (FGDs) were conducted with a total of 48 cohort participants in two of the intervention clusters. FGD participants were purposively selected to include a range of ages and MH product choices. 12-15 young women participated in each of four FGDs. In each of the two clusters, one FGD was conducted with 16-19-year-olds, and one was conducted with 20–24-year-olds.

Semi-structured topic guides were informed by findings from the MH pilot study and the preliminary analysis of the quantitative data. The topic guide explored participants' perspectives on the MH intervention, what they had learned and/or gained from the intervention, which MH products participants chose, if and how their experiences of managing menstruation had changed over time, how they felt about MH in general, and how COVID-19 had affected their lives and menstrual practices. All FGDs were conducted face-to-face by experienced female qualitative researchers (MT, TN, and PI) independent from the implementation team. FGDs were conducted in either Shona or English (as preferred by the participants) and took 60-75 minutes. Written informed consent was obtained before the FGDs were initiated and pseudonyms were used throughout for confidentiality and maintain anonymity. FGDs were audio recorded, transcribed verbatim, and then translated into English where necessary.

Analysis was guided by Braun and Clarke's approach to thematic analysis (30), using a mix of deductive and inductive themes (31). Initial deductive codes were based on the preliminary findings from the quantitative questionnaire, and included menstrual product choice and use, MH knowledge, attitudes around MH, and MH practices were based on preliminary findings from the quantitative questionnaire. Additional inductive codes were generated through transcript familiarisation, initial manual coding, and

For peer review only - http://bmjopen.bmj.com/site/about/guidelines.xhtml

analytical discussions between MT and CMY, and adapted through the coding process. These inductive codes and included "sociocultural norms," "stigma and taboo," and "myths". Deductive and inductive codes were merged to develop a coding framework, which was used to code all transcripts (32). Verbatim quotes were captured.

Qualitative analysis explored explanations and description about topics from the quantitative analysis. All the data was used to an provide in-depth understanding of and explanation on the effect of the MH intervention on MH knowledge, perceptions, and practices among cohort participants.

Patient and public involvement

This study included public involvement in the development of the MH intervention. The details of the co-development of CHIEDZA and the MH intervention nested within it have been previously described (33, 34). Briefly, we used FGDs and participatory workshops with young women aged 16-24 years old and other relevant stakeholders in the community including CHWs, local community-based organizations, and the Ministry of Health and Child Care (MoHCC) to investigate MH gaps in the community, develop the MH intervention, and inform the research questions for this study.

To identify the most relevant MH gaps and needs in our intervention communities, we worked with key stakeholders to inform a Theory of Change (ToC) for the MH intervention. The ToC articulated the critical pathways to improve key MH outcomes among young women in Zimbabwe and guided the development of the MH intervention components. The intervention was then piloted from April to June 2019 and feedback from both healthcare providers and clients were used to refine and inform the scaled-up implementation of the MH intervention.

No patients were involved in the research process, design of the study, the recruitment and conduct of the study, or the dissemination of study results.

Results

For peer review only

For peer review only - http://bmjopen.bmj.com/site/about/guidelines.xhtml

Participant characteristics

318 female clients were screened at two of the CHIEDZA clusters (Hatcliffe and Budiriro) between December 2019 and February 2020. Of those screened, two were outside the eligible age range and 13 (4.1%) declined to participate due to time constraints. A total of 303 participants were therefore enrolled in the cohort study (Figure 1).

Sociodemographic characteristics of participants at each timepoint are presented in Table 2. Of the 303 participants at baseline, half (51.2%) were aged 16–19 years old and half (51.8%) had completed O-level education (equivalent to the UK GCSE). One-third (35.0%) of participants were in school and 87.8% did not have a job. Half (51.2%) obtained money or income from a relative and 28.2% obtained money from a partner. 61.1% of participants had never married and all came from low- or moderate-income households. Of the 303 participants that were seen at baseline, 189 (62.4%) were seen at six-month visits, and 184 (60.7%) at 12-month visits. Overall, cohort participant characteristics were broadly similar at baseline, six-month visits, and 12-month visits.

Table 2 Sociodemographic characteristics and menstrual health outcomes at each time point

Sociodemographic characteristics of MH Cohort Participants						
	BASELINE (N=303)		SIX-MONTH VISIT (N=189)		12-MONTH VISIT (N=184)	
**Age**	N	%	N	%	N	%
16 - 19	155	51.2%	84	54.3%	68	53.3%
20 -24	148	48.8%	105	45.7%	116	46.7%
**Highest level of education**						
Less than primary school or primary school completed	110	36.3%	66	34.9%	55	29.9%
O-levels completed	157	51.8%	90	47.6%	97	52.7%
More than O-levels completed	36	11.9%	33	17.5%	32	17.4%
**Currently in school**						
Yes	106	35.0%	72	38.1%	61	33.2%

For peer review only

No	197	65.0%	117	61.9%	123	66.8%
**Paying job**						
Yes	37	12.2%	20	10.6%	29	15.8%
No	266	87.8%	169	89.4%	155	84.2%
**Marital status**						
Never married	185	61.1%	126	66.7%	114	61.9%
Married, or living as if married	110	36.3%	61	32.3%	64	34.8%
Single (widowed or divorced)	8	2.6%	2	1.0%	6	3.3%
**Religion**						
Not religious	8	2.6%	4	2.1%	3	1.6%
Christian	251	82.8%	154	81.5%	157	85.3%
Other	44	14.5%	31	16.4%	24	13.1%
**Household population**						
Lives alone	10	3.3%	0	0.0%	3	1.6%
Lives with 1-3 other people	116	38.3%	58	30.7%	55	29.9%
Lives with 4-6 other people	147	48.5%	101	53.4%	93	50.5%
Lives with >6 other people	30	9.9%	30	15.9%	33	17.9%
**Total household income**						
Low-income	149	49.2%	93	49.2%	84	45.7%
Moderate-income	154	50.8%	96	50.8%	100	54.3%
**Source of income[1]**						
I don't get money from anyone	59	19.6%	28	15.0%	35	19.4%
I get money from a relative	154	51.2%	105	56.5%	91	50.6%
I get money from my partner	85	28.2%	52	28.0%	52	28.9%
I get money from somewhere else	3	1.0%	1	0.5%	2	1.1%

1 missing data for "Source of income" sociodemographic characteristics at baseline (N=303), six-month visit (N=189), and 12-month visit (N=184)

MH knowledge, perceptions, and practices

For peer review only - http://bmjopen.bmj.com/site/about/guidelines.xhtml

For MH knowledge, perceptions, and MH practices for reusable pads, the mean scores and the proportion of participants with all correct or positive responses increased from baseline to six-month visit and decreased from six-month visit to 12-month visit (Table 3). For MH practices for menstrual cups, the mean score and proportion with all positive responses increased from baseline to six-month visit and from six-month visit to 12-month visit. For MH knowledge and perceptions, there was strong evidence of an increase in mean scores from baseline to six-month visit with MH knowledge mean score reflecting the highest improvement (1.62; 95% CI:1.27-1.97) over time. There was also an increase in mean score from baseline to 12-month visit for MH perceptions.

Table 3: MH outcome mean scores (M) and odds ratios (ORs) for the proportion answering all items correctly over time for participants at six-month visit vs baseline, and 12-month visit vs baseline respectively

	BASELINE (N=303)		SIX-MONTH VISIT (N=189)					12-MONTH VISIT (N=186)				
**MH Outcome**	**M**	**% with all correct/positive responses**	**M**	**% with all correct/positive responses**	**Absolute increase in mean score (95% CI)**	**aOR (95% CI)**	**p-value**	**M**	**% with all correct/positive responses**	**Absolute increase in mean score (95% CI)**	**aOR (95% CI)**	**p-value**
**Knowledge**	5.81	5.3%	7.45	42.3%	1.62 (1.27 – 1.97)	12.14 (6.77 – 21.76)	<0.001	6.92	32.6%	1.08 (0.73 – 1.42)	7.80 (4.3 – 14.16)	<0.001
**Perceptions**	2.54	9.2%	3.03	21.2%	0.49 (0.23 – 0.75)	2.85 (1.59 – 5.09)	<0.001	2.90	19.0%	0.36 (0.10 – 0.62)	2.53 (1.40 – 4.55)	<0.005
**Practices (reusable pads)**	4.41	14.2%	5.58	40.6%	1.17 (0.82 – 1.52)	4.68 (2.28 – 9.58)	<0.001	5.53	34.4%	0.96 (0.60 – 1.30)	3.64 (1.77 – 7.48)	<0.001
**Practices (menstrual cups)**	3.08	21.1%	3.80	40.6%	0.55 (0.40 – 1.15)	39.9 (0.05 – 34638.94)	<0.3	4.00	48.5%	0.95 (0.36 – 1.53)	1200.42 (0.44 – 3297620.00)	<0.1

**MH knowledge and MH practices (reusable pads) ORs adjusted for age and household population, MH perceptions OR adjusted for level of education, and MH practices (menstrual cups) OR adjusted for age*

For peer review only

After adjusting for confounders (age, level of education, and household population), there was strong evidence of an increase in MH knowledge, perceptions, and practices over time (Table 3). At six-month visit, participants were more likely to respond correctly or positively for all items for MH knowledge adjusted odds ratio (aOR)=12.14; 95%CI:6.8-21.8), MH perceptions (aOR=2.85; 95%CI:1.6-5.1), and practices for reusable pads (aOR=4.68; 95%CI:2.3-9.6) than at baseline. Similarly, there was strong evidence of an improved response on MH knowledge (aOR=7.8; 95%CI:4.3–14.2), MH perceptions (aOR=2.53; 95%CI:1.4– 4.6), and MH practices (reusable pads) (aOR=3.64; 95%CI:1.8–7.5) from baseline to 12-month visit. However, there was a slight decrease in odds of improved responses between six-month and 12-month visits for MH knowledge, perceptions, and practices for reusable pads.

Qualitative findings provided details and explanations to the increases in knowledge, perceptions, and practices. They explained how an unmet need for MH education, support, and products facilitated uptake of the MH intervention:

> *"All I want to say is CHIEDZA has helped me so much. For me, I no longer have stress to include pads on my grocery list,"* (Hatcliffe, FGD, 20 – 24 years old).

MH knowledge

Participants reported acquiring MH knowledge through the intervention. When asked questions about menstruation in the FGDs after the intervention, menstrual pain management, and how to use, wash, dry, and store the reusable menstrual products given to them through the MH intervention, almost all of the participants responded confidently and accurately. For example, when asked about use of the menstrual cup and "virginity" they responded:

> *"They (the CHIEDZA healthcare providers) explained… They said that it's not bad and it does not affect someone's virginity. I believe that it doesn't affect virginity,"* (Budiriro, FGD, 16 – 19 years old).

For peer review only - http://bmjopen.bmj.com/site/about/guidelines.xhtml

Their responses evidenced that participants had both retained the MH education from the intervention and gained additional MH knowledge on "best practices" based on their experiences using the products. When asked about washing and drying the reusable pads, participants responded:

> *"Don't wear [the pads] when damp… That's when it smells bad. It must dry properly because it's made of cotton…"* (Budiriro, FGD, 16 – 19 years old).

While most of the conversation around MH knowledge revealed accurate information retention, some of the responses around menstrual cups and pain management highlighted how sociocultural norms and persistent myths overrode MH education in the intervention. For example, when asked about menstrual pain, one participant said:

> *"For me, when I was a girl, I didn't experience period pain. It came after birth. I think I got it from my hospital bed. I think the bed I used was used by someone who had period pain,"* (Budiriro, FGD, 20 – 24 years old).

MH perceptions

After the intervention, participants reported feeling more confident about knowing how to manage their menstruation using the reusable menstrual products. Many participants also reported feeling "*proud*" and more positive about their menstruation. They described the MH intervention as reducing the shame they felt about menstruation. Some participants stated that they were determined to defy sociocultural norms that framed menstruation as a shameful "personal secret" no one else should know about:

> *"As for me, at our homestead there are boys but it's that, I dry my things [reusable pads] outside on the washing line because those boys they know about menstruation. They will marry their wives and they will also go on their monthly periods, even their mothers who gave birth to them, they go on their periods, it's not something new…"* (Budiriro, FGD, 16 – 19 years old).

Despite the MH education and support, however, some still faced stigma and expressed feeling shame and anxiety around menstruation. Fear of "smelling like fish" or leaking menstrual blood persisted and

For peer review only

informed MH product choice and practices. This shame around menstruation prevented them from feeling comfortable and confident during menstruation and when using and/or washing their menstrual products of choice:

> *"I thought it better to dry it [my pads] inside the house because people would want to know, what that is. They would also know about my period, when it started… so I will not feel alright,"* (Hatcliffe, FGD, 20 – 24 years old).

For some, longstanding sociocultural norms around menstruation overrode the positive menstrual perceptions gained through the intervention, and impacted MH practices, despite participants' gained knowledge.

> *"I stay with my aunt, my husband's sister. So, when I got the cup, she asked me if she can use the cup... The cup is not meant for girls, girls should use pads,"* (Hatcliffe, FGD, 20 – 24 years old).

MH practices

Participants were excited to have the opportunity to choose and use different types of menstrual products through the intervention. Product choice and menstrual practice was informed both by the knowledge gained through the intervention, as well as external sociocultural factors. Sociocultural beliefs around the menstrual cup causing the loss of "virginity" prevented many participants from using the cup, leading to low cup uptake. This was despite the issues of cup use and virginity being discussed during MH education sessions embedded in the MH intervention.

> *"Youths, they fear, and I'm one of them. I never attempted to use it because of fear, I was afraid to lose virginity, but I heard that if you use it, it's alright and you don't feel anything, everything will be normal,"* (Budiriro, FGD, 16 – 19 years old).

Since products were used away from the CHIEDZA intervention site, at home, the influence of sociocultural factors was strong.

For peer review only

For peer review only - http://bmjopen.bmj.com/site/about/guidelines.xhtml

> *"For me, I remember when we got these products, I was excited. When I got home, I showed my mom and she just said, 'I don't like that thing [the cup]'. So, you even become afraid of a thing which your mom does not approve of,"* (Budiriro, FGD, 20 – 24 years old).

While most participants chose not to use the menstrual cup, those that did, reported positive user experiences that encouraged them to continue cup use over time:

> *"I was scared at first to use the cup but when I started using it [chuckles]... It is very rare to see my pads on the washing line,"* (Hatcliffe, FGD, 20 -24 years old).

Environmental factors such as location (at home versus at work or school) and access to soap, water, and/or facilities to safely wash and dry reusable products also informed MH practices. These factors linked to the aforementioned perceptions of shame and anxiety around menstrual blood, with cleanliness felt to be a priority.

> *"There is a difference, if I'm at home and school, they are two different things. I don't use period pants when I'm at school because it does not handle blood for long time and the thing is you will be coming in and out in classroom… My colleagues will become suspicious. So, if I'm home I use period pants and when I'm school I use reusable pads."* (Budiriro, FGD, 16 – 19 years old).

> *"When travelling I prefer disposable pads because you never know where you go, you might not have water to use, and these things they need privacy, because when washing or drying them everyone there would be curious to know about it."* (Budiriro, FGD, 20 -24 years old).

For some, menstrual flow also informed MH practices, including using more than one menstrual product at a time.

> *"I can say first three days will be using a combination period pants and reusable pad. But last day will just use period pant."* (Budiriro, FGD, 16 – 19 years old).

Additional quotes from the FGDs are highlighted in Table 4.

For peer review only

For peer review only - http://bmjopen.bmj.com/site/about/guidelines.xhtml

Table 4 Summary of quotes from focus group discussion participants

MAIN THEMES	QUOTES FROM THE FOCUS GROUP DISCUSSIONS
MH KNOWLEDGE	*"When I clean them [reusable pads], I fetch water in a bucket, go to the bathroom, wash using green bar soap, rinse and put them on the washing line to dry, then put them in a bag and then in my drawer,"* (Hatcliffe, FGD, 20 – 24).<br><br>*"If you give birth, period pain goes away,"* (Hatcliffe, FGD, 20 – 24).<br><br>*"Don't wash them using detergents like washing powder, you should use green bar the dry them in sunlight for it to dry then you can you it again,"* (Hatcliffe, FGD, 16 – 19 years old).<br><br>*"They (CHIEDZA staff) explained, they said that the cup is not bad, and it does not affect someone's virginity… I believe that it doesn't affect virginity,"* (Budiriro, FGD, 16 – 19 years old).<br><br>*"We were told to boil the cup. It was said to destroy germs plus I think it can soften the hardness of it,"* (Budiriro, FGD, 20 -24 years old).
MH PERCEPTIONS	*"When I hear about CHIEDZA, I feel happy because I think of the products they provide for the girls in my community,"* (Hatcliffe, FGD, 16 – 19 years old).<br><br>*"I have difficulties when drying, at our homestead there are boys, so I dry my products inside our house…"* (Budiriro, FGD, 16 – 19 years old).<br><br>*"As for my mom, she said 'don't use that thing… Just wash other pads that you have.' She didn't want too much [more than that],"* (Budiriro, FGD, 16 -19 years old).<br><br>*"The disadvantages are people are not liking the cup especially girls they say that it makes then lose their virginity and some when they wear it, they feel like they are having sexual intercourse when walking of the tail,"* (Hatcliffe, FGD, 20 -24 years old).<br><br>*"I stay with my aunt, my husband's sister. So, when I got the cup, she asked me if she can use the cup... The cup is not meant for girls, girls should use pads."* (Hatcliffe, FGD, 20 – 24 years old).
MH PRACTICES	*"I can say girls with 16 - 19years are the ones who like reusable pads whilst the 20-24 they need cup,"* (Budiriro, FGD, 16 – 19 years old).<br><br>*"On my first days I use reusables then on my last days I used period pants because the blood flow will be minimal…"* (Hatcliffe, FGD, 16 – 19 years old).<br><br>*"[I wear] period pant first day of period because I will be having a light flow but other days, I will be using pads,"* (Budiriro, FGD, 16 – 19 years old).

For peer review only - http://bmjopen.bmj.com/site/about/guidelines.xhtml

	"When travelling I prefer disposable pads because you never know where you go, you might not have water to use, and these things they need privacy, because when drying them everyone there would be curious to know about it," (Budiriro, FGD, 20 – 24 years old.<br><br>*"Whenever I feel period pain, I take a pill,"* (Budiriro, FGD, 20 – 24 years old).

Discussion

This study provides evidence of improvements in key MH outcomes of knowledge, perceptions, and practices, through an intervention providing young women with MH products, education, and support in a community-based setting in urban Zimbabwe. The MH intervention led to knowledge acquisition on menstruation, menstrual pain management, and menstrual product use; increased confidence in menstrual management; and improved menstrual health practices. Sociocultural and environmental factors, such as myths and taboos around menstruation, cultural beliefs, and access to WASH facilities at home, school, or work, also informed the MH outcomes, but were outside the intervention's scope, and may have attenuated the positive effects of the intervention over time.

Our baseline findings add to evidence highlighting poor MH knowledge, perceptions, and practices, among young women in LMICs (6, 21). With MH shrouded in secrecy and taboo, we supplement evidence that young women often reach menarche with limited information, and sometimes misinformation, compounded by a lack of access to MH pain relief and products of choice, leading to poor experiences of managing menstruation (10, 20, 35). We therefore emphasise the need for comprehensive MH interventions, and provide evidence of an intervention to improve key MH outcomes.

The MH outcomes – knowledge, perceptions, and practices – were closely connected, highlighting the importance of integrated interventions targeting all three. Increased MH knowledge led to better MH practices and perceptions and better MH practices and perceptions led to better MH experiences overall. The value of offering choices of MH product enhanced MH experiences as young women could choose the product that best suited their circumstance, and led to increased experiential knowledge on how to

For peer review only

For peer review only - http://bmjopen.bmj.com/site/about/guidelines.xhtml

use, wash, dry, and store different products in young women's own specific environments. When encouraged to, participants were able to share their positive menstrual experiences and transfer that gained knowledge to others: changes in MH practices and perceptions thereby enabled MH information exchange and support (36). This process of acquiring and transferring MH knowledge and sharing menstrual experiences is often absent in most communities, without much needed MH discussion, correct information dissemination, and MH-related healthcare seeking behaviours (37-39).

The study highlights the influence of both the internal intervention components as well as external community social norms and access to WASH facilities at work, home, or school, as they interacted to inform young women's menstrual experiences and MH outcome measures (19). This understanding of the multifaceted nature of MH is consistent with the growing understanding that MH plays a key role in the lived experiences of women across the globe. Comprehensive MH interventions must thus address biological, personal, interpersonal, environmental, and societal MH-related gaps and barriers (5).

The decrease in all three outcomes from six-month to 12-month visits albeit small, underscores the need for interventions that address social norms which can otherwise impede long-term MH improvements. Previous studies have similarly had limited impact on social norms around menstruation. In a study looking at the effect of a community-based MH intervention for adolescent girls in India (40), results showed a significant improvement in MH practices, but only a marginal decline in the social and religious restrictions that informed attitudes towards menstruation. While there may be additional factors that informed the 12-month visit dip in MH knowledge, perceptions and practices in our study, our results suggest the need for MH interventions that include community members and prioritise community sensitisation and education around MH-related issues if there are to be acceptable, effective, and sustainable over time.

This is one of the first MH studies to assess the effectiveness of a comprehensive community-based MH intervention that provided both MH "hardware", through menstrual products, and "software", through MH education, for young women in a community-based setting (16). Unlike most MH interventions, our

For peer review only

study offered participants a choice of MH products and analgesics and used a mixed-methods approach to assess the effectiveness of this comprehensive MH intervention and the factors that informed MH product choice and patterns of use over time. Unusually, our study looks comprehensively at knowledge, perceptions, and practices together, and uses a validated measurement tool (MPNS-36) that has been used in other LMICs such as Uganda (19, 41).

In terms of limitations, there was substantial LTFU which may have led to bias. However, a comparison of participant sociodemographic characteristics at each visit showed no difference between those retained in the study and those LTFU. Qualitative data may have been prone to recall bias due to being collected retrospectively, and social desirability bias, due to the sensitive nature of the discussions. While observed differences before and after the study were assumed to be due to the intervention, with the lack of a control group, other external factors, such as natural growth, ageing or maturity, or other environmental changes may have influenced MH outcomes (42). While this was further explored qualitatively, temporal bias may be present. The relatively small number of participants solely from low-income urban settings in Harare, Zimbabwe, may not be generalisable.

Conclusion

Overall, the study results showed that the comprehensive MH intervention was effective in improving MH knowledge levels, perceptions, and practices among young women. Provision of a combination of comprehensive MH education, analgesics, a choice of MH products, and support over time were key to intervention effectiveness and success. While exposure and access to the youth-friendly service provision of MH education and resources facilitated improvements in MH outcome measures, these were mediated by external and contextual factors such as sociocultural norms and environmental conditions. It is important that MH gaps and barriers are tackled using a holistic approach that frames MH as a health and human rights issue and engages with both individuals and the wider communities in which they operate.

For peer review only

List of Abbreviations

CHW	Community Health Worker
FGD	Focus Group Discussion
IDI	In-Depth Interview
LMIC	Low- and Middle-Income Countries
MH	Menstrual Health
MHH	Menstrual Health and Hygiene
MHM	Menstrual Hygiene Management
MoHCC	Ministry of Health and Child Care
NGO	Non-Governmental Organisation
OR	Odds Ratio
RA	Research assistant
SDGs	Sustainable Development Goals
SRH	Sexual and Reproductive Health
UNICEF	United Nations Children's Fund
WASH	Water, Sanitation, and Health
WHO	World Health Organization

For peer review only

Declarations

Ethics approval and consent to participate

This study was approved by the Medical Research Council of Zimbabwe [MRCZ/A/2387], the London School of Hygiene and Tropical Medicine ethics committee [16124/RR/11602] and the Biomedical Research and Training Institute Institutional Review Board (AP149/2018). Our study did not include participants under 16 years old and written informed consent was obtained from all participants above the age of 16 years old before the survey and FGDs were initiated. Pseudonyms were used during the FGDs and in quotations for confidentiality and anonymity. All recordings were downloaded on to a password-secured laptop for processing purposes and deleted once the data analyses were complete.

Consent for publication

Not applicable

Availability of data and materials

The datasets used and/or analysed during the current study are available from the corresponding author on request.

Authors' contributions

MT designed the MH intervention, and collected, analysed, and interpreted the quantitative and qualitative data, and drafted the manuscript. CMY assisted in analysing the qualitative data and was a major contributor in writing the manuscript. HW assisted in analysing the quantitative data and was a major contributor in writing the manuscript. LL assisted in analysing the quantitative data. NG, PN, TN and PI assisted in the collection of the qualitative and quantitative data. TB and NR assisted with quantitative data collection and management. RAF, CDC, CVM, and ED contributed to the coordination of the study

For peer review only

513 and critical revisions of the manuscript. SF and JR contributed to design of the MH intervention and the
514 analysis plan. RAF is the Principal Investigator of the CHIEDZA Trial. All authors read and approved the
515 final manuscript.

516 ***Competing interests***

517 No competing interests.

518 ***Acknowledgements***

519 The authors would like to thank all the participants in the study.

520 ***Funding***

521 Swiss Agency for Development and Cooperation funding, the Wellcome Trust through a Senior Fellowship
522 to RAF (206316/Z/17/Z), and the Fogarty International Center of the National Institutes of Health under
523 Award Number D43 TW009539.

524

For peer review only

References

1. UNICEF. Guidance on Menstrual Health and Hygiene. www.unicef.org/wash: UNICEF, WASH; 2019 March.
2. Hennegan J, Winkler IT, Bobel C, Keiser D, Hampton J, Larsson G, et al. Menstrual health: a definition for policy, practice, and research. Sexual and Reproductive Health Matters. 2021;29(1):1911618.
3. Wilson LC, Rademacher KH, Rosenbaum J, Callahan RL, Nanda G, Fry S, et al. Seeking synergies: understanding the evidence that links menstrual health and sexual and reproductive health and rights. Sexual and Reproductive Health Matters. 2021;29(1):1882791.
4. Sommer M, Torondel B, Hennegan J, Phillips-Howard PA, Mahon T, Motivans A, et al. How addressing menstrual health and hygiene may enable progress across the Sustainable Development Goals. Global Health Action. 2021;14(1):1920315.
5. Hennegan J, Winkler IT, Bobel C, Keiser D, Hampton J, Larsson G, et al. Menstrual health: a definition for policy, practice, and research. Sexual and Reproductive Health Matters. 2021;29(1):31-8.
6. Hennegan J, Shannon AK, Rubli J, Schwab KJ, Melendez-Torres GJ. Women's and girls' experiences of menstruation in low- and middle-income countries: A systematic review and qualitative metasynthesis. PLoS Med. 2019;16(5):e1002803.
7. Hennegan J, Montgomery P. Do Menstrual Hygiene Management Interventions Improve Education and Psychosocial Outcomes for Women and Girls in Low and Middle Income Countries? A Systematic Review. PloS one. 2016;11(2):e0146985.
8. Sommer M, Caruso BA, Sahin M, Calderon T, Cavill S, Mahon T, et al. A Time for Global Action: Addressing Girls' Menstrual Hygiene Management Needs in Schools. PLoS Med. 2016;13(2):e1001962.
9. Sommer M, Schmitt M, Gruer C, Herbert A, Phillips-Howard P. Neglect of menarche and menstruation in the USA. Lancet. 2019;393(10188):2302.
10. Chandra-Mouli V, Patel SV. Mapping the knowledge and understanding of menarche, menstrual hygiene and menstrual health among adolescent girls in low- and middle-income countries. Reproductive Health. 2017;14(1):30.
11. Plesons M, Patkar A, Babb J, Balapitiya A, Carson F, Caruso BA, et al. The state of adolescent menstrual health in low- and middle-income countries and suggestions for future action and research. Reproductive Health. 2021;18(1):31.
12. VanLeeuwen C, Torondel B. Improving menstrual hygiene management in emergency contexts: literature review of current perspectives. Int J Womens Health. 2018;10:169-86.
13. Torondel B, Sinha S, Mohanty JR, Swain T, Sahoo P, Panda B, et al. Association between unhygienic menstrual management practices and prevalence of lower reproductive tract infections: a hospital-based cross-sectional study in Odisha, India. BMC infectious diseases. 2018;18(1):473-.
14. Phillips-Howard PA, Otieno G, Burmen B, Otieno F, Odongo F, Odour C, et al. Menstrual Needs and Associations with Sexual and Reproductive Risks in Rural Kenyan Females: A Cross-Sectional Behavioral Survey Linked with HIV Prevalence. J Womens Health (Larchmt). 2015;24(10):801-11.
15. Punzi MC. Technical Brief.
16. Hennegan J. Interventions to Improve Menstrual Health in Low- and Middle-Income Countries: Do We Know What Works? In: Bobel C, Winkler IT, Fahs B, Hasson KA, Kissling EA, Roberts T-A, editors. The Palgrave Handbook of Critical Menstruation Studies. Singapore: Springer Singapore; 2020. p. 637-52.
17. Evans RL, Harris B, Onuegbu C, Griffiths F. Systematic review of educational interventions to improve the menstrual health of young adolescent girls. BMJ Open. 2022;12(6):e057204.
18. Kuhlmann AS, Henry K, Wall LL. Menstrual Hygiene Management in Resource-Poor Countries. Obstetrical & Gynecological Survey. 2017;72(6):356-76.

For peer review only

For peer review only - http://bmjopen.bmj.com/site/about/guidelines.xhtml

19. Hennegan J, Nansubuga A, Smith C, Redshaw M, Akullo A, Schwab KJ. Measuring menstrual hygiene experience: development and validation of the Menstrual Practice Needs Scale (MPNS-36) in Soroti, Uganda. BMJ Open. 2020;10(2):e034461.
20. Tembo M, Renju J, Weiss HA, Dauya E, Bandason T, Dziva-Chikwari C, et al. Menstrual product choice and uptake among young women in Zimbabwe: a pilot study. Pilot Feasibility Stud. 2020;6(1):182.
21. UNICEF/Zimstats. Zimbabwe 2019 MICS Suvery Findings Report. 2019.
22. Ssewanyana D, Bitanihirwe BKY. Menstrual hygiene management among adolescent girls in sub-Saharan Africa. Glob Health Promot. 2019;26(1):105-8.
23. Ndlovu E, Bhala E. Menstrual hygiene - A salient hazard in rural schools: A case of Masvingo district of Zimbabwe. Jamba. 2016;8(2):204.
24. Dziva Chikwari C, Dauya E, Bandason T, Tembo M, Mavodza C, Simms V, et al. The impact of community-based integrated HIV and sexual and reproductive health services for youth on population-level HIV viral load and sexually transmitted infections in Zimbabwe: protocol for the CHIEDZA cluster-randomised trial [version 1; peer review: awaiting peer review]. Wellcome Open Research. 2022;7(54).
25. Kansiime C, Hytti L, Nalugya R, Nakuya K, Namirembe P, Nakalema S, et al. Menstrual health intervention and school attendance in Uganda (MENISCUS-2): a pilot intervention study. BMJ Open. 2020;10(2):e031182.
26. Endler M, Al-Haidari T, Benedetto C, Chowdhury S, Christilaw J, El Kak F, et al. How the coronavirus disease 2019 pandemic is impacting sexual and reproductive health and rights and response: Results from a global survey of providers, researchers, and policy-makers. Acta Obstetricia et Gynecologica Scandinavica.n/a(n/a).
27. Mackworth-Young CR, Chingono R, Mavodza C, McHugh G, Tembo M, Chikwari CD, et al. Community perspectives on the COVID-19 response, Zimbabwe. Bull World Health Organ. 2021;99(2):85-91.
28. Dzawanda B, Matsa M, Nicolau M. Poverty on the rise: The impact of the COVID-19 lockdown on the informal sector of Gweru, Zimbabwe. Int Soc Sci J. 2021:10.1111/issj.12285.
29. Boynton PM, Greenhalgh T. Selecting, designing, and developing your questionnaire. BMJ. 2004;328(7451):1312-5.
30. Braun V, Clarke V. Using thematic analysis in psychology. Qualitative Research in Psychology. 2006;3(2):77-101.
31. Fereday J, Muir-Cochrane E. Demonstrating Rigor Using Thematic Analysis: A Hybrid Approach of Inductive and Deductive Coding and Theme Development. International Journal of Qualitative Methods. 2006;5(1):80-92.
32. King N. Using templates in the thematic analysis of text. 2004 2021/04/08. In: Essential Guide to Qualitative Methods in Organizational Research [Internet]. London: SAGE Publications Ltd; [256-70]. Available from: http://sk.sagepub.com/books/essential-guide-to-qualitative-methods-in-organizational-research.
33. Mackworth-Young C, Dringus S, Dauya E, Chikwari C, Mavodza C, Tembo M, et al. Putting youth at the centre: co-design of a community-based intervention to improve HIV outcomes among youth in Zimbabwe. Wellcome Open Research. 2022;7:53.
34. Tembo M, Renju J, Weiss HA, Dauya E, Bandason T, Dziva-Chikwari C, et al. Menstrual product choice and uptake among young women in Zimbabwe: a pilot study. Pilot and Feasibility Studies. 2020;6(1):182.
35. Shanbhag D, Shilpa R, D'Souza N, Josephine P, Singh J, Goud B. Perceptions regarding menstruation and practices during menstrual cycles among high school going adolescent girls in resource limited settings around Bangalore city, Karnataka, India. International Journal of Collaborative Research on Internal Medicine & Public Health. 2012;4(7):1353.

For peer review only

For peer review only - http://bmjopen.bmj.com/site/about/guidelines.xhtml

36. Hennegan J, Dolan C, Steinfield L, Montgomery P. A qualitative understanding of the effects of reusable sanitary pads and puberty education: implications for future research and practice. Reprod Health. 2017;14(1):78.
37. McMahon SA, Winch PJ, Caruso BA, Obure AF, Ogutu EA, Ochari IA, et al. 'The girl with her period is the one to hang her head' Reflections on menstrual management among schoolgirls in rural Kenya. BMC Int Health Hum Rights. 2011;11:7.
38. Mahajan T. Imperfect Information in Menstrual Health and the Role of Informed Choice. Indian Journal of Gender Studies. 2019;26(1-2):59-78.
39. Chiou M-H, Wang H-H, Yang Y-H. Effect of Systematic Menstrual Health Education on Dysmenorrheic Female Adolescents' Knowledge, Attitudes, and Self-Care Behavior. The Kaohsiung journal of medical sciences. 2007;23:183-90.
40. A.R. Dongre PRD, Garg BS. The Effect of Community-Based Health Education Intervention on Management of Menstrual Hygiene among Rural Indian Adolescent Girls. World Health & Population. 2007;9(3):48-54.
41. Hennegan J, Brooks DJ, Schwab KJ, Melendez-Torres GJ. Measurement in the study of menstrual health and hygiene: A systematic review and audit. PloS one. 2020;15(6):e0232935.
42. Ho AMH, Phelan R, Mizubuti GB, Murdoch JAC, Wickett S, Ho AK, et al. Bias in Before–After Studies: Narrative Overview for Anesthesiologists. Anesthesia & Analgesia. 2018;126(5):1755-62.

For peer review only

Tables and Figures

Figure 1: Screening, recruitment, and follow-up flowchart for MH cohort study

Table 1: Questionnaire questions selected to measure menstrual health outcomes in the study

Table 2 : Sociodemographic characteristics and menstrual health outcomes at each time point

Table 3: MH outcome mean scores (M) and odds ratios (ORs) over time for participants at six-month visit vs baseline, and 12-month visit vs baseline respectively

Table 4: Summary of quotes from FGD participants

Additional Files

File Name: Appendix A

Format:.pdf

Title: Quantitative Questionnaire

Description: Complete quantitative questionnaire used in the MH cohort study

For peer review only

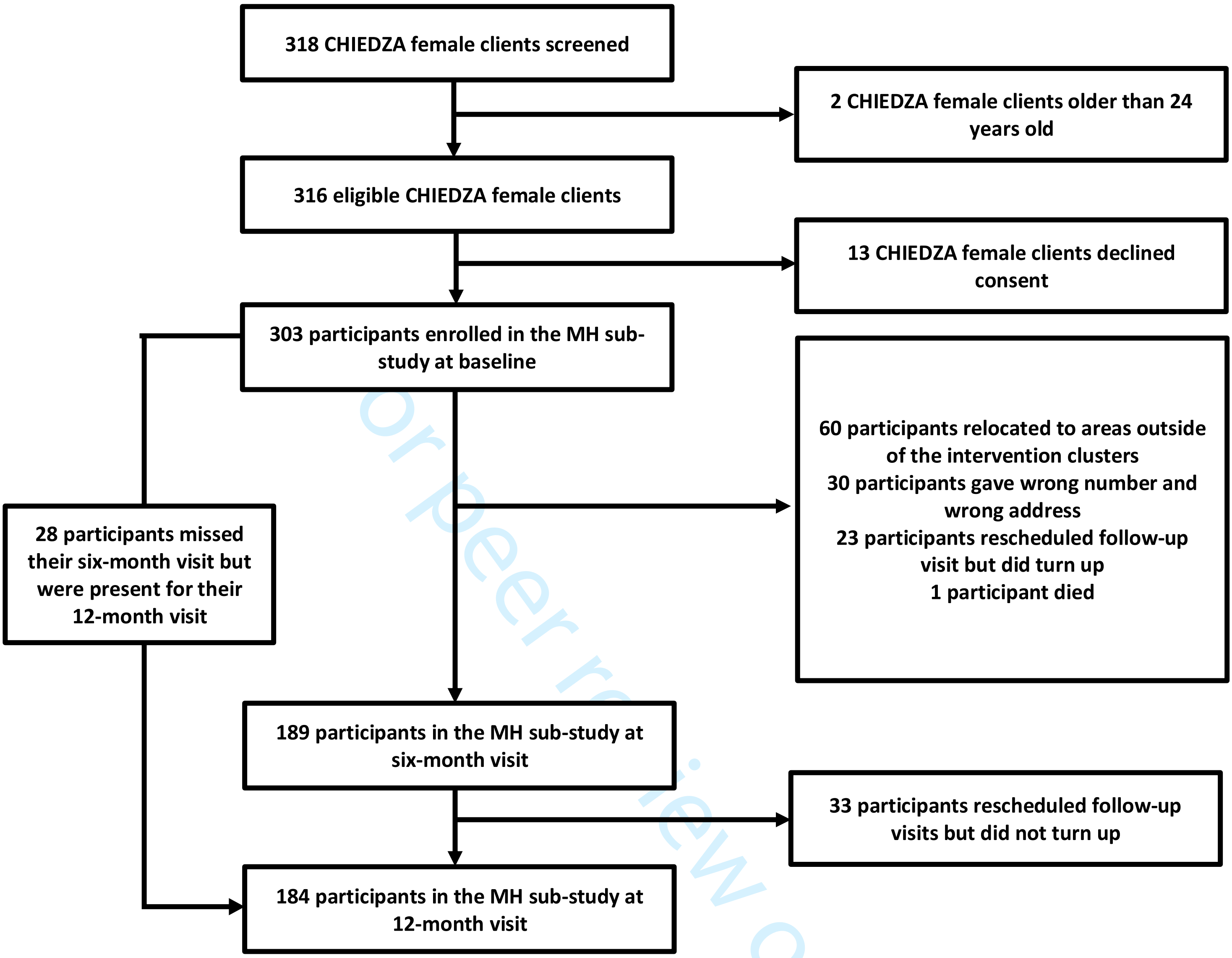

Figure 1 Screening, recruitment, and follow-up flow chart for MH cohort sub-study

For peer review only

MH STUDY ADMINISTRATION<br>This section is to be filled out by the attending staff member			
**A00**	*STAFFNO*	Staff Initials	☐☐☐
**A01**	*STUDYNO*	Study Participant Cohort Number	☐☐☐☐☐☐
**A02**	*DOB*	Date of Birth (dd/mm/yyyy)	☐☐/☐☐/☐☐☐☐
**A03**	*AGE*		☐☐
**A04**	*DATE*	Date of visit (dd/mm/yyyy)	☐☐/☐☐/20☐☐
**A05**	*VISIT*	Type of visit	Initial ☐ 6m☐ 12m☐

This questionnaire is to be filled out by participants in the menstrual health study

The purpose of this questionnaire is to collect information about you and how you feel and behave during your period.

There are 9 parts to this questionnaire. It should not take more than 30 minutes of your time. Please read the questions carefully and fill in the questionnaire as honestly as possible.

Thank you for your participation.

PART I: SOCIODEMOGRAPHIC INFORMATION

These questions are to help understand more about your life

**A06**	*EDU*	Highest Level of Education	Please tick your highest level of education:<br>No Formal School Education ☐<br>Incomplete Primary School ☐<br>Complete Primary School ☐<br>Incomplete High School Education ☐<br>Complete High School Education (O'Levels) ☐<br>Complete High School Education (A'Levels) ☐<br>Incomplete Tertiary Education ☐

			Complete University ☐
**A07**	*JOB*	Do you have a paying job?	Yes ☐ No ☐
**A08**	*MONEY*	If you do not work, where do you get your money	I do not get money from anyone ☐<br>I get money from a relative in the household (mother/ father/brother/sister etc.) ☐<br>I get money from a relative outside the household ☐<br>I get money from my partner ☐<br>Other ☐<br>If you chose "Other", please list here: ___________
**A09**	*MSTAT*	Marital Status	Single (Never Married) ☐<br>Married, or living as if you are married ☐<br>Widowed ☐<br>Divorced/Separated ☐
**A10**	*RES*	Residence	Urban ☐<br>Peri-Urban ☐<br>Rural ☐
**A11**	*RELGN*	Religion	Not Religious ☐<br>Traditional ☐<br>Christian (Pentecostal) ☐<br>Christian (Protestant) ☐<br>Roman Catholic ☐<br>Other ☐

For peer review only

			If you chose "Other", please list here: __________
**A12**	*HH*	Please tick any items your household has:	Please tick all that apply:<br>Television ☐<br>Radio ☐<br>Mobile Phone ☐<br>Electricity ☐<br>Water source inside the house ☐<br>Latrine inside the house ☐
**A13**	*INC*	How many people live in your household with you:	None (I live alone) ☐<br>1 – 3 other people ☐<br>4 – 6 other people ☐<br>More than 6 people ☐
**PART II: GENERAL QUESTIONS ABOUT YOURSELF**<br>**These questions are about you and why you came to the CHIEDZA site**			
**A14**	*CHIEDZA 1*	What brought you to CHIEDZA today?	Please tick all that apply:<br>STI Treatment ☐<br>Menstrual Hygiene Management ☐<br>HIV Testing ☐<br>HIV Treatment ☐<br>Condoms ☐<br>Family Planning ☐<br>Counselling ☐

For peer review only

			Other ☐<br>If you chose "Other", please list here: ___________
**A15**	*CHIEDZA 2*	Would you come to CHIEDZA if you could not receive MH products and information?	Yes ☐ No ☐
**PART III: PERIOD and PUBERTY KNOWLEDGE**<br>**These questions are about your understanding of puberty and periods**			
**QUESTIONS ABOUT PUBERTY AND MENSTRUATION**			
**A16**	*KNW1*	Changes in the body during puberty happen because of hormones	Yes ☐ No ☐ I don't know ☐
**A17**	*KNW2*	Puberty continues throughout a girl's life	Yes ☐ No ☐ I don't know ☐
**A18**	*KNW2*	Menstruation in girls and women is normal	Yes ☐ No ☐ I don't know ☐
**A19**	*KNW3*	When a girl gets her first menstrual period, her body is able to carry a child	Yes ☐ No ☐ I don't know ☐
**A20**	*KNW4*	Menstrual blood is caused by the breakdown of the lining of the womb	Yes ☐ No ☐ I don't know ☐
**A21**	*KNW5*	It is normal to have irregular periods as a teenager	Yes ☐ No ☐ I don't know ☐
**A22**	*KNW6*	Menstruation continues throughout a girl's life	Yes ☐ No ☐ I don't know ☐
**A23**	*KNW7*	A period normally lasts 2 days or less	Yes ☐ No ☐ I don't know ☐
**A24**	*KNW8*	Period products that are inserted into the vagina	Yes ☐ No ☐ I don't know ☐

For peer review only

		(such as tampons and the menstrual cup) affect your virginity	
**PART IV: QUESTIONS ABOUT YOUR PERIOD HISTORY**<br>**The following questions are about you and your menstrual periods**			
The following questions are about your period history			
**A25**	*MENARCHE*	How old were you when you first started your periods?	Age ☐☐<br>I don't remember ☐
**A26**	*TAUGHT*	Who first told you or taught you about periods	No one told me about periods ☐<br>My mother ☐<br>My father ☐<br>My female/male friend ☐<br>My teacher ☐<br>Other ☐<br>If you chose "Other", please list here: ___________
**A27**	*INFO1*	Do you feel you had enough information and education to manage your period when you got your first period?	Yes ☐<br>Somewhat ☐<br>Not Really ☐<br>Definitely No ☐
**A28**	*INFO2*	What did you think was happening when you got your first period?	Curse ☐<br>Disease ☐<br>Injury ☐<br>Normal function for young women ☐<br>I did not know what was happening ☐

For peer review only

			Other ☐<br>If you chose "Other", please list here: ___________
**A29**	*DAYS*	On average, how many days do you usually bleed for during your periods?	1 – 3 days ☐<br>4 – 7 days ☐<br>More than 7 days ☐
**A30**	*RCNTD AYS*	How many days did you bleed during your most recent period?	☐☐<br>I Do Not Remember ☐
**PART V: QUESTIONS ABOUT PAIN MANAGEMENT**<br>**The following questions are about menstruation-related pain**			
**A31**	*PAINK NW1*	Painkillers can be used to relieve menstrual pain	Yes ☐ No ☐ I don't know ☐
**A32**	*PAINK NW2*	Painkillers can cause problems having children (barrenness)	Yes ☐ No ☐ I don't know ☐
**A33**	*PAINK NW3*	Exercising, stretching, or putting a warm towel or bottle on your stomach can relieve menstrual pain	Yes ☐ No ☐ I don't know ☐
During my last (most recent) menstrual period…			
**A34**	*PAIN1*	I experienced period-related pain in my lower back and/or abdomen	Yes ☐ No ☐
**A35**	*PAIN2*	I missed work or school due to period-related pain	Yes ☐ No ☐
**PART VI:**<br>**QUESTIONS ABOUT PRACTICES AND PERCEPTIONS DURING MENSTRUATION**<br>**The following questions are about how you manage your menstruation**			
The following questions are about your last (most recent) period			

For peer review only

**A36**	*PRDCT*	During your most recent period, what material(s)did you mostly use to collect or absorb your period blood?	Please tick all that apply:<br>Cloth/Rags/Fabric ☐<br>Reusable Pads ☐<br>Disposable Pads ☐<br>Menstrual Cup ☐<br>Period Pants ☐<br>Other ☐<br>Not Applicable ☐<br>If you chose "Other", please list here: ___________
**A37**	*HPRDCT*	During your most recent period, what material(s)did you mostly use to collect or absorb your period blood when at home?	Please tick all that apply:<br>Cloth/Rags/Fabric ☐<br>Reusable Pads ☐<br>Disposable Pads ☐<br>Menstrual Cup ☐<br>Period Pants ☐<br>Other ☐<br>Not Applicable ☐<br>If you chose "Other", please list here: ___________
**A38**	*SWPRDCT*	During your most recent period, what material(s)did you mostly use to collect or absorb your period blood when at school/work?	Please tick all that apply:<br>Cloth/Rags/Fabric ☐<br>Reusable Pads ☐<br>Disposable Pads ☐<br>Menstrual Cup ☐<br>Period Pants ☐

For peer review only

			Other ☐<br>Not Applicable ☐<br>If you chose "Other", please list here: ___________
During my last (most recent) menstrual period…			
**A39**	*PRDCT 1*	My menstrual products were comfortable	Strongly Disagree ☐<br>Disagree ☐<br>Agree ☐<br>Strongly Agree ☐
**A40**	*PRDCT 2*	I had enough of my menstrual products to change them as often as I wanted to	Strongly Disagree ☐<br>Disagree ☐<br>Agree ☐<br>Strongly Agree ☐
**A41**	*PRDCT 3*	Before I used them, I was satisfied with the cleanliness of my menstrual products	Strongly Disagree ☐<br>Disagree ☐<br>Agree ☐<br>Strongly Agree ☐
**A42**	*PRDCT 4*	I could get more of my menstrual materials when I needed to	Strongly Disagree ☐<br>Disagree ☐<br>Agree ☐<br>Strongly Agree ☐
**A43**	*PRDCT 5*	I was worried that my menstrual products would allow blood to pass through my outer garments	Strongly Disagree ☐<br>Disagree ☐<br>Agree ☐<br>Strongly Agree ☐
**A44**	*PRDCT 6*	I was worried that my menstrual products would	Strongly Disagree ☐<br>Disagree ☐

For peer review only

		move from place while I was wearing them	Agree ☐<br>Strongly Agree ☐
**A45**	*PRDCT 7*	I worried about how I would get more of my menstrual material if I ran out	Strongly Disagree ☐<br>Disagree ☐<br>Agree ☐<br>Strongly Agree ☐
**A46**	*PRDCT 8*	I felt comfortable carrying spare menstrual products with me outside my home	Strongly Disagree ☐<br>Disagree ☐<br>Agree ☐<br>Strongly Agree ☐
**A47**	*PRDCT 9*	I felt comfortable carrying menstrual products to the place where I changed them	Strongly Disagree ☐<br>Disagree ☐<br>Agree ☐<br>Strongly Agree ☐
**A48**	*PRDCT 10*	I felt comfortable storing my menstrual products until my next period	Strongly Disagree ☐<br>Disagree ☐<br>Agree ☐<br>Strongly Agree ☐
**A49**	*PRDCT 11*	I was able to wash my hands when I wanted to	Strongly Disagree ☐<br>Disagree ☐<br>Agree ☐<br>Strongly Agree ☐
**If participant ticks "Disposable Pads" or "Cloth/Rags/Fabric"**			
During my last (most recent) period…			
**B1**	*DIS1*	I was able to immediately dispose of my used menstrual material	Strongly Disagree ☐<br>Disagree ☐<br>Agree ☐

For peer review only

			Strongly Agree ☐
**B2**	*DIS2*	I was able to dispose of my used menstrual material in the way I wanted to	Strongly Disagree ☐<br>Disagree ☐<br>Agree ☐<br>Strongly Agree ☐
**B3**	*DIS3*	I worried about where to dispose of my used menstrual materials	Strongly Disagree ☐<br>Disagree ☐<br>Agree ☐<br>Strongly Agree ☐
**B4**	*DIS4*	I was concerned that others would see my menstrual materials in the place I disposed them	Strongly Disagree ☐<br>Disagree ☐<br>Agree ☐<br>Strongly Agree ☐
**If participant ticks "Cloth/Rags/Fabric", "Period Pants" and/or "Reusable Pads":**			
During my last (most recent) period…			
**C1**	*REUS1*	I had enough water to soak or wash my menstrual products	Strongly Disagree ☐<br>Disagree ☐<br>Agree ☐<br>Strongly Agree ☐
**C2**	*REUS2*	I had access to a basin or dish to soak or wash my menstrual products whenever I needed	Strongly Disagree ☐<br>Disagree ☐<br>Agree ☐<br>Strongly Agree ☐
**C3**	*REUS3*	I was able to wash my menstrual products when I wanted to	Strongly Disagree ☐<br>Disagree ☐<br>Agree ☐<br>Strongly Agree ☐

For peer review only

**C4**	*REUS4*	I had enough soap to wash my menstrual products	Strongly Disagree ☐<br>Disagree ☐<br>Agree ☐<br>Strongly Agree ☐
**C5**	*REUS5*	I was able to dry my products <u>when</u> I wanted to	Strongly Disagree ☐<br>Disagree ☐<br>Agree ☐<br>Strongly Agree ☐
**C6**	*REUS6*	I was worried that someone would see me while I was washing my menstrual products	Strongly Disagree ☐<br>Disagree ☐<br>Agree ☐<br>Strongly Agree ☐
**C7**	*REUS7*	I was worried that my menstrual products would not be dry when I needed them	Strongly Disagree ☐<br>Disagree ☐<br>Agree ☐<br>Strongly Agree ☐
**C8**	*REUS8*	I was worried that others would see my menstrual products while they were drying	Strongly Disagree ☐<br>Disagree ☐<br>Agree ☐<br>Strongly Agree ☐
**C9**	*REUS9*	How often did you change your menstrual products during the heaviest day of your most recent period?	Once a day (within a 24hr period) ☐<br>Twice a day (within a 24hr period) ☐<br>Three times a day (within a 24hr period) ☐<br>More than three times a day (within a 24hr period) ☐
**C10**	*REUS10*	At home, where did you most often change your menstrual products during your most recent period?	In a toilet inside the building ☐<br>In a toilet outside the building ☐<br>In a private room ☐<br>Outside (in the field/by the river) ☐

For peer review only

			Other ☐<br>If you chose "Other", please explain: ___________
**C11**	*REUS11*	At school/work, where did you most often change your menstrual products during your most recent period?	In a toilet inside the building ☐<br>In a toilet inside the building ☐<br>In a toilet outside the building ☐<br>In a private room ☐<br>Outside (in the field/by the river) ☐<br>Other ☐<br>Not applicable ☐<br>If you chose "Other", please explain: ___________
**C12**	*REUS12*	Where did you wash your menstrual products during your last period?	In the bathroom ☐<br>At a private well or tap in the house ☐<br>At a public well or tap outside the house ☐<br>In a pond or river ☐<br>Did not wash your menstrual products ☐<br>Other ☐<br>If you chose "Other", please explain: ___________
**C13**	*REUS13*	What did you use to wash your products with during your most recent period?	With water only ☐<br>With soap and water ☐<br>Did not wash my menstrual products ☐<br>Other ☐<br>If you chose "Other", please explain: ___________
**C14**	*REUS14*	Where did you dry your menstrual products during your most recent period?	Outside in the sun ☐<br>Outside in the sun, under a cloth ☐<br>Inside in the house in an open space ☐

For peer review only

			Inside the house in a closed space ☐<br>Did not dry my reusable pad, period pants, or cloth/rags/fabric ☐<br>Other ☐<br>If you chose "Other", please explain: ___________
**C15**	*REUS15*	Did you ever wear your menstrual product when it was damp (not yet dry) during your most recent period?	Yes ☐ (No ☐<br>Do Not Remember ☐
**C16**	*REUS16*	Where did you store your menstrual products after your most recent period?	In a cupboard ☐<br>In the toilet ☐<br>In an open space ☐<br>In a hidden place ☐<br>Did not store ☐<br>Other ☐<br>If you chose "Other", please explain: ____________
**If participant ticks "Menstrual Cup":**			
During my last (most recent) period…			
**D1**	*MC1*	I was able to sterilize my menstrual cup <u>when</u> I wanted after my period	Strongly Disagree ☐<br>Disagree ☐<br>Agree ☐<br>Strongly Agree ☐
**D2**	*MC2*	I was able to rinse my menstrual cup <u>when</u> I wanted	Strongly Disagree ☐<br>Disagree ☐<br>Agree ☐<br>Strongly Agree ☐

For peer review only

**D3**	*MC3*	I had enough water to rinse my menstrual cup	Strongly Disagree ☐<br>Disagree ☐<br>Agree ☐<br>Strongly Agree ☐
**D4**	*MC4*	I was worried that someone would see me while I was rinsing my menstrual cup	Strongly Disagree ☐<br>Disagree ☐<br>Agree ☐<br>Strongly Agree ☐
**D5**	*MC5*	I was worried that someone would see me while I was sterilizing my menstrual cup	Strongly Disagree ☐<br>Disagree ☐<br>Agree ☐<br>Strongly Agree ☐
**D6**	*MC6*	How long did it take you to get comfortable and relaxed using your menstrual cup?	1 Period ☐<br>2 Periods ☐<br>3 Periods ☐<br>4 or More Periods ☐<br>Still Not Comfortable or Relaxed ☐
**D7**	*MC7*	What is difficult about using your menstrual cup?	Tick All that Apply:<br>Could not get the cup in ☐<br>Too much pain and discomfort ☐<br>Difficult to clean ☐<br>Difficult to remove ☐<br>Other ☐<br>If you chose "Other", please explain: ___________
**D8**	*MC8*	How often did you empty and re-insert your menstrual cup during the heaviest day of your most recent period?	Once a day (within a 24hr period) ☐<br>Twice a day (within a 24hr period) ☐<br>Three times a day (within a 24hr period) ☐<br>More than three times a day (within a 24hr period) ☐

For peer review only

**D9**	*MC9*	At home, where did you most often empty and re-insert your menstrual cup when you needed to during your most recent period?	In a toilet inside the building ☐<br>In a toilet outside the building ☐<br>Outside (in the field/by the river) ☐<br>Other ☐<br>If you chose "Other", please explain: ___________
**D10**	*MC10*	At school/work, where did you most often empty and re-insert your menstrual cup when you needed to during your most recent period?	In a toilet inside the building ☐<br>In a toilet outside the building ☐<br>Outside (in the field/by the river) ☐<br>Not Applicable ☐<br>Other ☐<br>If you chose "Other", please explain: ___________
**D11**	*MC11*	Where did you rinse your menstrual cup during your last period?	In a private bathroom ☐<br>In a public bathroom ☐<br>At a private well or tap in the house ☐<br>At a public well or tap outside the house ☐<br>In a pond or river ☐<br>Did not rinse my menstrual cup ☐<br>Other ☐<br>If you chose "Other", please explain: ___________
**D12**	*MC12*	What did you rinse your menstrual cup with during your most recent period?	With water only ☐<br>With soap and water ☐<br>Did not rinse my menstrual cup ☐<br>Other ☐<br>If you chose "Other", please explain: ___________

For peer review only

**D13**	*MC13*	During your most recent period, how did you sterilise your menstrual cup?	Boiled cup for 3 min ☐<br>Washed with boiling water ☐<br>Soaked in vinegar and water solution ☐<br>Other ☐<br>If you chose "Other", please explain: ___________
**D14**	*MC14*	Did you store your menstrual cup in an aerated bag between periods?	Yes ☐ No ☐
**D15**	*MC15*	During your most recent period, did you ever use a reusable or disposable pad along with the menstrual cup?	Yes ☐ No ☐
**PART V: QUESTIONS ABOUT YOUR WASHING PRACTICES**<br>**The following questions are about how you wash your body and your vagina during your last (most recent) period**			
**A50**	*WASH1*	What type of washing did you do when you were on your most recent period?	External genital wash only ☐<br>Full body wash ☐<br>I did not wash myself ☐<br>Other ☐<br>If you chose "Other", please explain: ___________
**A51**	*WASH2*	On average, how many times did you wash your body during your most recent period?	Less than once a day ☐<br>Once a day ☐<br>Twice a day ☐<br>Three times a day or more ☐
**A52**	*WASH3*	What did you use to wash yourself during your most recent period?	Water only ☐<br>Soap and water ☐<br>Not Applicable ☐<br>Other ☐<br>If you chose "Other", please explain: ___________
**PART VII: QUESTIONS ABOUT YOUR ATTITUDES ABOUT MENSTRUATION**<br>**These questions are about how feel about menstruation**			

For peer review only

**A53**	*ATTITUDE1*	I feel dirty or impure during my menstrual period	Strongly Disagree ☐<br>Disagree ☐<br>Neutral ☐<br>Agree ☐<br>Strongly Agree ☐
**A54**	*ATTITUDE2*	I feel like I can talk to friends about menstruation	Strongly Disagree ☐<br>Disagree ☐<br>Neutral ☐<br>Agree ☐<br>Strongly Agree ☐
**A55**	*ATTITUDE3*	I feel like I can talk to my family members about menstruation	Strongly Disagree ☐<br>Disagree ☐<br>Neutral ☐<br>Agree ☐<br>Strongly Agree ☐
**A56**	*ATTITUDE4*	I feel proud that I have my periods	Strongly Disagree ☐<br>Disagree ☐<br>Neutral ☐<br>Agree ☐<br>Strongly Agree ☐
**A57**		It is important that I keep my period a secret from everyone	Strongly Disagree ☐<br>Disagree ☐<br>Neutral ☐<br>Agree ☐<br>Strongly Agree ☐
**A58**	*ATTITUDE5*	I worry that boys will tease me about my period	Strongly Disagree ☐<br>Disagree ☐<br>Neutral ☐

For peer review only

			Agree ☐<br>Strongly Agree ☐
**A59**	*ATTITUDE6*	I am anxious about my next period	Strongly Disagree ☐<br>Disagree ☐<br>Neutral ☐<br>Agree ☐<br>Strongly Agree ☐
**PART VIII: PARTICIPATION**<br>**The following questions are about how your period may affect your everyday activities**			
**A60**	*PRTCPTN1*	In the last 3 months, have you missed work/school because of your period?	Yes ☐ No ☐<br>**If "No", skip next question**
**A61**	*PRTCPTN2*	Why did you miss work/school?	Please tick all that apply:<br>Pain ☐<br>Nothing To Use To Manage My Period ☐<br>No place to change or wash ☐<br>Ashamed or embarrassed ☐<br>Scared I will leak ☐<br>Told to go home/Not allowed ☐
**A62**	*PRTCPTN3*	In the last 3 months, have you missed social events or community/religious happenings because of your period?	Yes ☐ No ☐<br>**If "No", skip next question**
**A63**	*PRTCPTN4*	Why did you miss the social events or social/religious happenings?	Please tick all that apply:<br>Pain ☐<br>Nothing To Use To Manage My Period ☐<br>No place to change or wash ☐<br>Ashamed or embarrassed ☐

For peer review only

			Scared I will leak ☐<br>Told to go home/Not allowed ☐
**A64**	*PRTCPT N5*	In the last 3 months, what everyday activities have you missed out on because of your period?	Please tick all that apply:<br>Household activities (washing clothes, dishes, etc) ☐<br>Sports (athletics, running, swimming, etc) ☐<br>Income-generating activities ☐<br>Religious activities ☐<br>Socialising with friends and family ☐<br>School ☐
**PART IX: FACTORS AT HOME**<br>**The following questions are about the surroundings in which you manage your period at home**			
At home during my last (most recent) period…			
**A65**	*FHOME 1*	I was able to change my menstrual products <u>when</u> I wanted to	Strongly Disagree ☐<br>Disagree ☐<br>Agree ☐<br>Strongly Agree ☐
**A66**	*FHOME 2*	I was satisfied with the place I used to change my menstrual products	Strongly Disagree ☐<br>Disagree ☐<br>Agree ☐<br>Strongly Agree ☐
**A67**	*FHOME 3*	I had a clean place to change my menstrual products	Strongly Disagree ☐<br>Disagree ☐<br>Agree ☐<br>Strongly Agree ☐
**A68**	*FHOME 4*	I was worried I would not be able to change my menstrual products <u>when</u> I needed to	Strongly Disagree ☐<br>Disagree ☐<br>Agree ☐<br>Strongly Agree ☐
**A69**	*FHOME 5*	I was worried someone would	Strongly Disagree ☐

For peer review only

		see me while I was changing my menstrual products	Disagree ☐<br>Agree ☐<br>Strongly Agree ☐
**A70**	*FHOME 6*	I was worried someone would harm me while I was changing my menstrual products	Strongly Disagree ☐<br>Disagree ☐<br>Agree ☐<br>Strongly Agree ☐
**A71**	*FHOME 7*	I was worried something else would harm me while I was changing my menstrual products	Strongly Disagree ☐<br>Disagree ☐<br>Agree ☐<br>Strongly Agree ☐
**A72**	*FHOME 8*	Do you have access to a clean water source at your home	Yes ☐ No ☐<br>**If "No", skip next question**
**A73**	*FHOME 9*	Where is your clean water source located?	Please tick all that apply:<br>Inside the toilet ☐<br>Outside the toilet but inside the home ☐<br>Outside the house but inside the yard of my home ☐<br>In a public space ☐
**A74**	*FHOME 10*	Do you have access to a toilet at home	Yes ☐ No ☐<br>**If "No", skip next question**
**A75**	*FHOME 11*	Where is your toilet located?	Inside the house ☐<br>Outside the house but inside the yard ☐<br>In a public space ☐
**A76**	*FHOM12*	Does your toilet have a door and a working lock on the inside (for privacy)?	Yes ☐ No ☐
**PART IX: FACTORS AT SCHOOL/WORK**<br>**The following questions are about how you manage your period at school/work**			

For peer review only

At school/work during my last period…			
**A77**	*FSW1*	I was able to change my menstrual products when I wanted to	Strongly Disagree ☐<br>Disagree ☐<br>Agree ☐<br>Strongly Agree ☐<br>Not Applicable ☐
**A78**	*FSW2*	I was satisfied with the place I used to change my menstrual products	Strongly Disagree ☐<br>Disagree ☐<br>Agree ☐<br>Strongly Agree ☐<br>Not Applicable ☐
**A79**	*FSW3*	I had a clean place to change my menstrual materials	Strongly Disagree ☐<br>Disagree ☐<br>Agree ☐<br>Strongly Agree ☐<br>Not Applicable ☐
**A80**	*FSW4*	I was worried I would not be able to change my menstrual material when I needed to	Strongly Disagree ☐<br>Disagree ☐<br>Agree ☐<br>Strongly Agree ☐<br>Not Applicable ☐
**A81**	*FSW5*	I was worried someone would see me while I was changing my menstrual products	Strongly Disagree ☐<br>Disagree ☐<br>Agree ☐<br>Strongly Agree ☐<br>Not Applicable ☐
**A82**	*FSW6*	I was worried someone would	Strongly Disagree ☐

For peer review only

		harm me while I was changing my menstrual products	Disagree ☐<br>Agree ☐<br>Strongly Agree ☐<br>Not Applicable ☐
**A83**	*FSW7*	I was worried something else would harm me while I was changing my menstrual products	Strongly Disagree ☐<br>Disagree ☐<br>Agree ☐<br>Strongly Agree ☐<br>Not Applicable ☐
**A84**	*FSW8*	Do you have access to a toilet at school/work?	Yes ☐ No ☐<br>Not Applicable ☐<br>**If "No" or "Not Applicable", skip next 2 questions**
**A85**	*FSW9*	Where is your toilet located?	Inside the building ☐<br>Outside the building but inside the school/work yard ☐<br>In a public area, outside the school/work yard ☐
**A88**	*FSW12*	Does your toilet have a door and a working lock on the inside (for privacy)?	Yes ☐ No ☐
**A86**	*FSW10*	Do you have access to water at school/work?	Yes ☐ No ☐<br>Not Applicable ☐<br>**If "No" or "Not Applicable", skip next question**
**A87**	*FSW11*	Where is your water source located?	Please tick all that apply:<br>Inside the toilet ☐<br>Outside the toilet but inside the school/work building ☐<br>Outside the building but inside the school/work yard ☐<br>In a public area, outside the school/work yard ☐

THANK YOU FOR YOUR TIME AND YOUR ANSWERS!

For peer review only

For peer review only

Reporting checklist for cohort study.

Based on the STROBE cohort guidelines.

Instructions to authors

Complete this checklist by entering the page numbers from your manuscript where readers will find each of the items listed below.

Your article may not currently address all the items on the checklist. Please modify your text to include the missing information. If you are certain that an item does not apply, please write "n/a" and provide a short explanation.

Upload your completed checklist as an extra file when you submit to a journal.

In your methods section, say that you used the STROBE cohortreporting guidelines, and cite them as:

von Elm E, Altman DG, Egger M, Pocock SJ, Gotzsche PC, Vandenbroucke JP. The Strengthening the Reporting of Observational Studies in Epidemiology (STROBE) Statement: guidelines for reporting observational studies.

		Reporting Item	Page Number
**Title and abstract**			
Title	#1a	Indicate the study's design with a commonly used term in the title or the abstract	3
Abstract	#1b	Provide in the abstract an informative and balanced summary of what was done and what was found	3-4
**Introduction**			
Background / rationale	#2	Explain the scientific background and rationale for the investigation being reported	6-7
Objectives	#3	State specific objectives, including any prespecified hypotheses	7
**Methods**			
Study design	#4	Present key elements of study design early in the paper	7

For peer review only

Setting	#5	Describe the setting, locations, and relevant dates, including periods of recruitment, exposure, follow-up, and data collection	7-8
Eligibility criteria	#6a	Give the eligibility criteria, and the sources and methods of selection of participants. Describe methods of follow-up.	8-9
Eligibility criteria	#6b	For matched studies, give matching criteria and number of exposed and unexposed	N/A<br>Not a matched study
Variables	#7	Clearly define all outcomes, exposures, predictors, potential confounders, and effect modifiers. Give diagnostic criteria, if applicable	10-12
Data sources / measurement	#8	For each variable of interest give sources of data and details of methods of assessment (measurement). Describe comparability of assessment methods if there is more than one group. Give information separately for for exposed and unexposed groups if applicable.	10-12
Bias	#9	Describe any efforts to address potential sources of bias	10
Study size	#10	Explain how the study size was arrived at	9
Quantitative variables	#11	Explain how quantitative variables were handled in the analyses. If applicable, describe which groupings were chosen, and why	9
Statistical methods	#12a	Describe all statistical methods, including those used to control for confounding	
9-10			
Statistical methods	#12b	Describe any methods used to examine subgroups and interactions	9-10
Statistical methods	#12c	Explain how missing data were addressed	9-10
Statistical methods	#12d	If applicable, explain how loss to follow-up was addressed	9-10
Statistical methods	#12e	Describe any sensitivity analyses	

For peer review only

N/A

Results

Participants	#13a	Report numbers of individuals at each stage of study—eg numbers potentially eligible, examined for eligibility, confirmed eligible, included in the study, completing follow-up, and analysed. Give information separately for for exposed and unexposed groups if applicable.	14
Participants	#13b	Give reasons for non-participation at each stage	14
Participants	#13c	Consider use of a flow diagram	

14

Descriptive data	#14a	Give characteristics of study participants (eg demographic, clinical, social) and information on exposures and potential confounders. Give information separately for exposed and unexposed groups if applicable.	15
Descriptive data	#14b	Indicate number of participants with missing data for each variable of interest	

14

Descriptive data	#14c	Summarise follow-up time (eg, average and total amount)	

14-15

Outcome data	#15	Report numbers of outcome events or summary measures over time. Give information separately for exposed and unexposed groups if applicable.	

16-17

Main results	#16a	Give unadjusted estimates and, if applicable, confounder-adjusted estimates and their precision (eg, 95% confidence interval). Make clear which confounders were adjusted for and why they were included	17
Main results	#16b	Report category boundaries when continuous variables were categorized	16-17
Main results	#16c	If relevant, consider translating estimates of relative risk into absolute risk for a meaningful time period	

For peer review only

N/A			
Other analyses	#17	Report other analyses done—eg analyses of subgroups and interactions, and sensitivity analyses	17-22
**Discussion**			
Key results	#18	Summarise key results with reference to study objectives	23
Limitations	#19	Discuss limitations of the study, taking into account sources of potential bias or imprecision. Discuss both direction and magnitude of any potential bias.	24
Interpretation	#20	Give a cautious overall interpretation considering objectives, limitations, multiplicity of analyses, results from similar studies, and other relevant evidence.	24
Generalisability	#21	Discuss the generalisability (external validity) of the study results	24-25
**Other Information**			
Funding	#22	Give the source of funding and the role of the funders for the present study and, if applicable, for the original study on which the present article is based	28

The STROBE checklist is distributed under the terms of the Creative Commons Attribution License CC-BY. This checklist was completed on 23. August 2022 using https://www.goodreports.org/, a tool made by the EQUATOR Network in collaboration with Penelope.ai

For peer review only

BMJ Open

A mixed-methods study measuring the effectiveness of a menstrual health intervention on menstrual health knowledge, perceptions, and practices among young women in Zimbabwe

Journal:	*BMJ Open*
Manuscript ID	bmjopen-2022-067897.R1
Article Type:	Original research
Date Submitted by the Author:	02-Jan-2023

Complete List of Authors:	Tembo, Mandikudza ; London School of Hygiene and Tropical Medicine Faculty of Public Health and Policy, Department of Global Health and Development; Biomedical Research and Training Institute<br>Weiss, Helen; London School of Hygiene and Tropical Medicine Faculty of Epidemiology and Population Health<br>Larsson, Leyla; Biomedical Research and Training Institute,<br>Bandason , Tsitsi; Biomedical Research and Training Institute<br>Redzo, Nicol; Biomedical Research and Training Institute<br>Dauya, E; Biomedical Research and Training Institute<br>Nzanza, Tafadzwa; Biomedical Research and Training Institute<br>Ishumael, Pauline; Biomedical Research and Training Institute<br>Gweshe, Nancy; Biomedical Research and Training Institute<br>Ndlovu, Precious; Biomedical Research and Training Institute<br>Dziva Chikwari, Chido; London School of Hygiene & Tropical Medicine<br>Mavodza, Constancia ; Biomedical Research and Training Institute; London School of Hygiene & Tropical Medicine, Department of Public Health, Environments and Society<br>Renju, Jenny; London School of Hygiene & Tropical Medicine<br>Francis, Suzanna ; London School of Hygiene & Tropical Medicine<br>Ferrand, Rashida; Biomedical Research and Training Institute; London School of Hygiene and Tropical Medicine Department of Clinical Research, Clinical Research Department<br>Mackworth-Young, Constance; Biomedical Research and Training Institute; London School of Hygiene and Tropical Medicine Department of Global Health and Development, Department of Global Health and Development

**Primary Subject Heading**:	Public health
Secondary Subject Heading:	Health services research, Qualitative research, Sexual health
Keywords:	PUBLIC HEALTH, Public health < INFECTIOUS DISEASES, QUALITATIVE RESEARCH

SCHOLARONE™
Manuscripts

I, the Submitting Author has the right to grant and does grant on behalf of all authors of the Work (as defined in the below author licence), an exclusive licence and/or a non-exclusive licence for contributions from authors who are: i) UK Crown employees; ii) where BMJ has agreed a CC-BY licence shall apply, and/or iii) in accordance with the terms applicable for US Federal Government officers or employees acting as part of their official duties; on a worldwide, perpetual, irrevocable, royalty-free basis to BMJ Publishing Group Ltd ("BMJ") its licensees and where the relevant Journal is co-owned by BMJ to the co-owners of the Journal, to publish the Work in this journal and any other BMJ products and to exploit all rights, as set out in our licence.

The Submitting Author accepts and understands that any supply made under these terms is made by BMJ to the Submitting Author unless you are acting as an employee on behalf of your employer or a postgraduate student of an affiliated institution which is paying any applicable article publishing charge ("APC") for Open Access articles. Where the Submitting Author wishes to make the Work available on an Open Access basis (and intends to pay the relevant APC), the terms of reuse of such Open Access shall be governed by a Creative Commons licence – details of these licences and which Creative Commons licence will apply to this Work are set out in our licence referred to above.

Other than as permitted in any relevant BMJ Author's Self Archiving Policies, I confirm this Work has not been accepted for publication elsewhere, is not being considered for publication elsewhere and does not duplicate material already published. I confirm all authors consent to publication of this Work and authorise the granting of this licence.

For peer review only

Title:

A mixed-methods study measuring the effectiveness of a menstrual health intervention on menstrual health knowledge, perceptions, and practices among young women in Zimbabwe

Mandikudza Tembo[1,2,5], Helen A Weiss[1], Leyla Larsson[2], Tsitsi Bandason[2], Nicol Redzo[2], Ethel Dauya[2], Tafadzwa Nzanza[2], Pauline Ishumael[2], Nancy Gweshe[2], Precious Ndlovu[2], Chido Dziva Chikwari[1,2], Constancia Vimbayi Mavodza[2,4], Jenny Renju[1], Suzanna C. Francis[1], Rashida A.Ferrand[2,3] , Constance R.S. Mackworth-Young[2,5]

Affiliations:

1. *MRC International Statistics & Epidemiology Group, London School of Hygiene and Tropical Medicine, United Kingdom*
2. *Biomedical Research and Training Institute, Harare, Zimbabwe*
3. *Clinical Research Department, London School of Hygiene and Tropical Medicine, United Kingdom*
4. *Department of Public Health, Environments and Society, Faculty of Public Health and Policy, London School of Hygiene and Tropical Medicine, London, United Kingdom*
5. *Department of Global Health and Development, Faculty of Public Health and Policy, London School of Hygiene and Tropical Medicine, London, United Kingdom*

Email addresses of authors:

Helen Weiss: Helen.Weiss@lshtm.ac.uk

Leyla Larsson: llarsson3@outlook.com

Tsitsi Bandason: tbandason@brti.co.zw

Nicol Redzo: nredzo@brti.co.zw

For peer review only

Ethel Dauya: edauya@brti.co.zw

Tafadzwa Nzanza: nzanzatafadzwa@gmail.com

Pauline Ishumael: chidoishy@gmail.com

Nancy Gweshe: nuncii@gmail.com

Precious Ndlovu: meloosie@gmail.com

Chido Dziva Chikwari: Chido.DzivaChikwari@lshtm.ac.uk

Constancia Mavodza: Constancia-Vimbayi.Mavodza@lshtm.ac.uk

Jenny Renju: Jenny.Renju@lshtm.ac.uk

Suzanna Francis: Suzanna.Francis@lshtm.ac.uk

Rashida Ferrand: Rashida.Ferrand@lshtm.ac.uk

Constance Mackworth-Young: constance.mackworth-young1@lshtm.ac.uk

Keywords:

Menstrual health, knowledge, effectiveness, adolescent health, menstruation, practices

§ Corresponding Author:

Mandikudza M P Tembo

London School of Hygiene and Tropical Medicine,

Phone: +263772 676 417

Email: mandikudza.tembo@lshtm.ac.uk

ORCID: 0000-0002-4520-3317

For peer review only

Abstract

Objectives: While integral to women's physical and mental wellbeing, achieving good menstrual health (MH) remains a challenge for many women. This study investigated the effectiveness of a comprehensive MH intervention on menstrual knowledge, perceptions, and practices among women aged 16-24 years in Harare, Zimbabwe.

Design: A mixed-methods prospective cohort study with pre-post evaluation of an MH intervention.

Setting: Two intervention clusters in Harare, Zimbabwe.

Participants: Overall, 303 female participants were recruited, of whom 189 (62.4%) were seen at midline (median follow-up 7.0; IQR:5.8–7.7 months) and 184 (60.7%) were seen at endline (median follow-up 12.4; IQR:11.9-13.8 months). Cohort follow-up was greatly affected by COVID-19 pandemic and associated restrictions.

Intervention: The MH intervention provided MH education and support, analgesics, and a choice of menstrual products in a community-based setting to improve MH outcomes among young women in Zimbabwe

Primary and secondary outcomes: Effectiveness of a comprehensive MH intervention on improving MH knowledge, perceptions, and practices among young women over time. Quantitative questionnaire data was collected at baseline, midline, and endline. At endline, thematic analysis of four focus group discussions was used to further explore participants' menstrual product use and experiences of the intervention.

Results: At midline, more participants had correct/positive responses for MH knowledge (adjusted odds ratio (aOR)=12.14; 95%CI:6.8-21.8), perceptions (aOR=2.85; 95%CI:1.6-5.1), and practices for reusable pads (aOR=4.68; 95%CI:2.3-9.6) than at baseline. Results were similar comparing endline with baseline for all MH outcomes. Qualitative findings showed that sociocultural norms, stigma, and taboos around

For peer review only - http://bmjopen.bmj.com/site/about/guidelines.xhtml

menstruation, and environmental factors such as limited access to water, sanitation, and hygiene facilities affected the effect of the intervention on MH outcomes.

Conclusions: The intervention improved menstrual health knowledge, perceptions, and practices among young women in Zimbabwe, and the comprehensive nature of the intervention was key to this. MH interventions should address interpersonal, environmental, and societal factors.

Trial registration

Registry: Clinicaltrials.gov

Registration Number: NCT03719521

Registration Date: October 25, 2018

For peer review only

Strengths and limitations of the study

- This is one of the first menstrual health studies to assess the effectiveness of a multicomponent menstrual health intervention that provided both “hardware”, through a choice of menstrual products and analgesics, and “software”, through menstrual health education and support, for young women in a community-based setting
- The study used a validated measuring tool (MPNS-36) that has been used in other low- to middle-income countries to comprehensively assess the effect of the menstrual health intervention on key menstrual health outcomes, adding to a key evidence gap in robust quantitative research in menstrual health outcome measures
- The study used a mixed-methods approach that used both qualitative and quantitative analysis to comprehensively assess effects of the intervention on menstrual health outcomes over time, adding to the research supporting the implementation of comprehensive evidence-based menstrual health interventions to improve poor menstrual health indicators across the globe
- Qualitative data may have been prone to social desirability bias due to the sensitive nature of the discussions around menstruation, and recall bias due to being collected retrospectively
- The conclusions of this study may be limited due to the lack of a control group, therefore other unmeasured environmental or external factors, such as natural growth and maturity, may have influenced the observed outcomes

For peer review only - http://bmjopen.bmj.com/site/about/guidelines.xhtml

Introduction

Menstrual health (MH) encompasses a state of physical and psychosocial wellbeing that is informed by access to MH knowledge and products and supportive environment (1-4). MH is integral to women's health and overall wellbeing (5) but MH remains a challenge for many women, especially those in low- and middle-income countries (LMICs) (6, 7). Faced with the stigma and taboo surrounding menstruation, the lack of access to affordable menstrual products and inadequate quality MH education and support, MH remains a critically unaddressed issue (8-12). Many girls and women experience shame, anxiety, and/or untreated pain during their menstruation as they are forced to use ineffective or unhygienic alternatives such as old cloth or poor-quality disposable pads to manage their menstruation. In some instances, this lack of access to MH products and/or education on how to use menstrual products properly can lead to harmful reproductive tract infections or dangerous practices such as transactional sex in effort to access better menstrual products (13, 14).

Acceptable and effective MH interventions are integral to addressing and improving women's health outcomes (3, 15). Informed by a growing understanding of the challenges that women face in managing menstruation, interventions have been implemented in different contexts, especially in LMICs (7, 16). A systematic review of MH interventions and their effect on health outcomes among women in LMICs cautiously reported that MH interventions can inform some positive changes in MH-related outcomes such as MH knowledge levels and menstrual attitudes (7). The review highlighted several gaps and weaknesses in MH intervention research. Many MH intervention studies tend to focus on school-going girls (17) and exclusively address either access to MH "hardware" such as menstrual products and water, sanitation, and hygiene (WASH) facilities in which to manage menstruation, or access to MH "software" such as MH education and support targeted at demystifying and destigmatizing MH-related issues, but rarely both (7, 16, 18). Moreover, without validated and standardized tools and measures for MH

For peer review only - http://bmjopen.bmj.com/site/about/guidelines.xhtml

outcomes more broadly, most studies lack robust evaluations, making it difficult to reliably assess the effect of interventions on MH-related outcomes (7, 19).

Evidence-based MH interventions that address access to menstrual products, pain management, and the larger systematic factors that inform menstrual experiences are needed in Zimbabwe, as elsewhere (20). In 2019, 21.8% of women aged 15–19 years and 18.8% of women aged 20–24 years in Zimbabwe reported not participating in social activities, school, or work in the last 12 months due to menstruation (21). Another study, by Stitching Netherlandse Vrijwilligers (SNV), conducted in 203 schools in Masvingo, Zimbabwe reported that 72% of girls in rural Zimbabwe had never used sanitary pads (22). Many girls and women in low-income areas throughout Zimbabwe face challenges in accessing comfortable and effective MH products and have to improvise with old cloth or newspaper (23). Many also face challenges in accessing safe and clean WASH facilities to manage their menstruation at home, school, or work. Some are unable to participate in social activities or concentrate on their work or duties due to unaddressed menstrual pain or discomfort and fear of leaking in public as they are forced to use ineffective old rags, cotton wool, or tissue paper to manage their menstruation (24, 25).

The aim of this study was to address this evidence gap by investigating the effectiveness of a comprehensive MH intervention integrated within a community-based sexual and reproductive health (SRH) service on MH knowledge, perceptions, and practices among young women in Zimbabwe.

Methods

Study design and setting

We conducted a prospective cohort study from December 2019 to August 2021, using quantitative and qualitive methods to assess MH outcomes. The study was nested within a cluster randomised trial of community-based integrated HIV and SRH intervention for young people aged 16-24 years (CHIEDZA trial registration number in clinical trials.gov: NCT03719521) (26). CHIEDZA provided a package of free-of-

For peer review only

For peer review only - http://bmjopen.bmj.com/site/about/guidelines.xhtml

153 charge SRH services that included HIV testing and treatment, condoms, risk reduction counselling,
154 contraception and testing and treatment of sexually transmitted infections and an MH intervention
155 (described below) in a youth-friendly environment. CHIEDZA was delivered in 12 intervention clusters in
156 three provinces across Zimbabwe (Harare, Bulawayo, and Mashonaland East) from April 2019 to March
157 2022. Within each province, the CHIEDZA services were delivered by a team of trained healthcare
158 providers comprising two youth workers, one counsellor, two nurses, and four community health workers
159 (CHWs). Participants in the cohort study were followed up every three months for a year.
160 At baseline and six-month and 12-month visits, participants completed a quantitative questionnaire. At
161 the three-month visit, participants handed-in their completed period tracking diaries and at nine-months,
162 participants were given another period-tracking diary to be completed and handed in at the 12-month
163 visit.

164 ***The menstrual health intervention***

165 The MH intervention was preceded by a pilot (from April-July 2019) in all four Harare intervention clusters
166 (20). Results from the pilot were used to refine and inform the scale-up of the intervention across all 12
167 CHIEDZA intervention clusters. The final MH intervention available to all female CHIEDZA clients, from
168 April 2019 to March 2022, included comprehensive MH education and support, provision of a period-
169 tracking diary, two pairs of normal underwear, a choice between either reusable pads (AFRIpads that can
170 be used for up to two years) (www.afripads.com) or the menstrual cup (the Butterfly Cup that can used
171 for up to ten years) (www.vivalily.com), as well as soap, pain management advice and monthly pain
172 medication (a choice between paracetamol or ibuprofen). Reusable pads and menstrual cups were used
173 in the MH intervention as these sustainable MH products were both cost-effective and environmentally-
174 friendly.

175 ***Cohort participants***

For peer review only - http://bmjopen.bmj.com/site/about/guidelines.xhtml

176 A subset of CHIEDZA female clients from the Harare intervention clusters (Budiriro and Hatcliffe) were recruited to the prospective cohort study by two research assistants (RAs) (NG and PN) between December 2019 to February 2020. Equal numbers were sought in two age-strata (16-19 and 20-24 years old) until a cohort size of 318 was reached. Clients were excluded if 1) they were currently pregnant or intended to get pregnant within the next year; 2) they experienced irregular or absent bleeding within the last 3 months; or 3) had any condition that precluded informed consent or made study participation unsafe. Written informed consent was obtained from all participants in the cohort study. No parental consent was needed to uptake the MH intervention as national guidelines allow for those aged 16 years and older to give independent consent to accessing SRH services.

185 Those who consented to participate in the cohort study received two period tracking diaries and three different types of reusable menstrual products including: 1) reusable pads; 2) a menstrual cup; and 3) three pairs of period pants (VivaLily period pants that can be used for up to two years).

188 Overall, the MH intervention pilot was from April to July 2019, scale-up of MH intervention across all 12 CHIEDZA intervention clusters commenced in July 2019, and recruitment of a subset of CHIEDZA clients for the MH cohort began in December 2019.

191 ***Sample size and COVID-19 disruptions***

192 The planned sample size of 300 cohort participants provided 90% power to detect an increase in the proportion of participants answering all MH knowledge questions correctly from 10% at baseline to 20% at six-month visit or 12-month visit (assuming p=0.05). This calculation was based on results from a pre-post study of an MH intervention among school-girls in Uganda (27).

196 COVID-19 and Zimbabwe's response strategies impacted the overall CHIEDZA trial (28, 29). Unable to go to school or work and sustain their livelihoods, many people either sent their children to, or migrated to, the rural areas in search of a more affordable environment (30). Such migration had implications on cohort follow-up and overall exposure to the MH intervention.

For peer review only - http://bmjopen.bmj.com/site/about/guidelines.xhtml

Quantitative data collection and analysis

A quantitative questionnaire informed by findings from the pilot intervention (20) and Hennegan's Menstrual Practice Needs Scale (MPNS-36) (19), was used to collect data on: 1) sociodemographic information; 2) menstruation and puberty knowledge; 3) menstrual history; 4) pain management; 5) practices and perceptions during menstruation; 6) washing practices; and 7) attitudes about menstruation. All questions were translated into Shona and back-translated into English with input from MT, NG, and PN. The survey was piloted with a subset of 30 CHIEDZA female clients that took up the MH intervention and iteratively revised prior to cohort recruitment (31). The full questionnaire can be found in the supplementary materials.

The self-administered questionnaire was completed at baseline, six-month visit, and 12-month visit. To maximise participation, we reminded participants of upcoming visits via phone. We contacted participants up to three times to reschedule if participants didn't attend their scheduled visit, and offered a home visit before considering the participant lost to follow-up (LTFU) if they did not attend within four weeks of their scheduled visit. If participants later visited CHIEDZA again, we opportunistically invited participants to complete the questionnaire.

For this paper, relevant questions were selected from the questionnaire prior to analysis to assess the effect of the intervention on MH knowledge, perceptions, and practices (for reusable pads and for the menstrual cup) over time (Table 1). The data were analysed using Stata version 16.1 (StatCorp, Texas, USA). Descriptive statistics were used to describe participants' self-reported sociodemographic characteristics, including age, highest level of education, religion, marital status, employment, school, source of income, household income, and household population at baseline, six-month visit, and 12-month visit. We investigated whether LTFU might potentially bias findings by comparing the sociodemographic characteristics of those present at each time-point.

Table 1 Questionnaire questions selected to measure menstrual health outcomes in the study

For peer review only - http://bmjopen.bmj.com/site/about/guidelines.xhtml

MH Outcome Measures	Questions	Binary Scale	
MH knowledge	Changes in the body during puberty happen because of hormones	0=No/Don't know	1=Yes
	Puberty continues throughout a girl's life	1=No	0=Yes/Don't know
	Menstruation in girls and women is normal	0=No/Don't know	1=Yes
	When a girl gets her first menstrual period, her body can carry a child	0=No/Don't know	1=Yes
	Menstrual blood is caused by the breakdown of the lining of the womb	0=No/Don't know	1=Yes
	It is normal to have irregular periods as a teenager	0=No/Don't know	1=Yes
	Menstruation continues throughout a girl's life	1=No	0=Yes/Don't know
	A period normally lasts 2 days or less	1=No	0=Yes/Don't know
	Period products that are inserted into the vagina (such as tampons and the menstrual cup) affect your virginity	1=No	0=Yes/Don't know
	**Highest possible score for MH knowledge**	**9**	
MH perceptions	I feel dirty or impure during my menstrual period	0=Neutral/Agree/Strongly Agree	1=Disagree/Strongly Disagree
	I feel like I can talk to friends about menstruation	1=Agree/Strongly Agree	0=Neutral/Disagree/Strongly Disagree
	I feel like I can talk to my family members about menstruation	1=Agree/Strongly Agree	0=Neutral/Disagree/Strongly Disagree
	It is important that I keep my period secret for everyone	0=Neutral/Agree/Strongly Agree	1=Disagree/Strongly Disagree
	I worry that boys will tease me about my period	0=Neutral/Agree/Strongly Agree	1=Disagree/Strongly Disagree
	I am anxious about my next period	0=Neutral/Agree/Strongly Agree	1=Disagree/Strongly Disagree
	**Highest possible score for MH perceptions**	**6**	

For peer review only

MH practices (reusable pads)	I had enough water to soak or wash my menstrual products	1=Agree/Strongly Agree	0=Neutral/Disagree/Strongly Disagree
	I was able to wash my menstrual products when I wanted to	1=Agree/Strongly Agree	0=Neutral/Disagree/Strongly Disagree
	I had enough soap to wash my menstrual products when I wanted to	1=Agree/Strongly Agree	0=Neutral/Disagree/Strongly Disagree
	I was able to dry my products when I wanted to	1=Agree/Strongly Agree	0=Neutral/Disagree/Strongly Disagree
	I was worried that someone would see me while I was washing my menstrual product	0=Neutral/Agree/Strongly Agree	1=Disagree/Strongly Disagree
	I was worried that my menstrual products would not dry when I needed them	0=Neutral/Agree/Strongly Agree	1=Disagree/Strongly Disagree
	I was worried that others would see my menstrual products while they were drying	0=Neutral/Agree/Strongly Agree	1=Disagree/Strongly Disagree
	**Highest possible score for MH practices (reusable pads)**	**7**	
MH practices (menstrual cups)	I was able to sterilize my menstrual cup when I wanted after my period	1=Agree/Strongly Agree	0=Neutral/Disagree/Strongly Disagree
	I was able to rinse my menstrual cup when I wanted	1=Agree/Strongly Agree	0=Neutral/Disagree/Strongly Disagree
	I had enough water to rinse my menstrual cup	1=Agree/Strongly Agree	0=Neutral/Disagree/Strongly Disagree
	I was worried that someone would see me while I was rinsing my menstrual cup	0=Neutral/Agree/Strongly Agree	1=Disagree/Strongly Disagree
	I was worried that someone would see me while I was sterilizing my menstrual cup	0=Neutral/Agree/Strongly Agree	1=Disagree/Strongly Disagree
	**Highest possible score for MH practices (menstrual cups)**	**5**	

At each time-point, and for each of the MH-related outcomes: MH knowledge, perceptions, practices for reusable pad use, and practices for menstrual cup use, we calculated the average score of relevant items and the proportion of participants who answered all relevant items either “correctly” or “positively”. We

For peer review only - http://bmjopen.bmj.com/site/about/guidelines.xhtml

used repeated measures logistic regression to assess the pre-post change in the proportion of participants with correct/positive outcomes from baseline to six-moth visit, and baseline to 12-month visit respectively. Similarly, we used repeated measures linear regression to assess the pre-post change in scores for continuous variables from baseline to six-month visit, and baseline to 12-month respectively. We investigated potential confounders by assessing the association between each sociodemographic variable with exposure (visit time-point) and each binary MH outcome measure. The variable was considered a potential confounder if association with both the outcome and exposure had a p<0.05. If the crude odds ratio or regression coefficient changed by more than 10% on adjusting for the potential confounder, the variable was considered a confounder and was retained in the model.

Qualitative data collection and analysis

Following the 12-month visits, after exposure to the intervention, four focus group discussions (FGDs) were conducted with a total of 48 cohort participants in two of the intervention clusters. FGD participants were purposively selected to include a range of ages and MH product choices. 12-15 young women participated in each of four FGDs. In each of the two clusters, one FGD was conducted with 16-19-year-olds, and one was conducted with 20–24-year-olds.

Semi-structured topic guides were informed by findings from the MH pilot study and the preliminary analysis of the quantitative data. The topic guide explored participants' perspectives on the MH intervention, what they had learned and/or gained from the intervention, which MH products participants chose, if and how their experiences of managing menstruation had changed over time, how they felt about MH in general, and how COVID-19 had affected their lives and menstrual practices. All FGDs were conducted face-to-face by experienced female qualitative researchers (MT, TN, and PI) independent from the implementation team. FGDs were conducted in either Shona or English (as preferred by the participants) and took 60-75 minutes. Written informed consent was obtained before the FGDs were initiated and pseudonyms were used throughout for confidentiality and maintain anonymity. National

For peer review only

guidelines indicate that parental consent is required for those under 18 years old to participate in research but due to the risk of desirability bias affecting responses and the minimal risk associated with participation in FGDs and IDIs, a waiver for parental consent for participants 16–18 years old was obtained from the ethics review bodies. FGDs were audio recorded, transcribed verbatim, and then translated into English where necessary.

Analysis was guided by Braun and Clarke's approach to thematic analysis (32), using a mix of deductive and inductive themes (33). All transcripts were read for familiarisation and manually coded before importing them to NVivo 12. Initial deductive codes were based on the preliminary findings from the quantitative questionnaire, and included menstrual product choice and use, MH knowledge, attitudes around MH, and MH practices were based on preliminary findings from the quantitative questionnaire. Additional inductive codes were generated through transcript familiarisation, initial manual coding, and analytical discussions between MT and CMY, and adapted through the coding process. These inductive codes and included "sociocultural norms," "stigma and taboo," and "myths". Deductive and inductive codes were merged to develop a coding framework, which was used to code all transcripts (34). Verbatim quotes were captured.

Qualitative analysis explored explanations and description about topics from the quantitative analysis. All the data was used to an provide in-depth understanding of and explanation on the effect of the MH intervention on MH knowledge, perceptions, and practices among cohort participants.

Patient and public involvement

This study included public involvement in the development of the MH intervention. The details of the co-development of CHIEDZA and the MH intervention nested within it have been previously described (35, 36). Briefly, we used FGDs and participatory workshops with young women aged 16-24 years old and other relevant stakeholders in the community including CHWs, local community-based

For peer review only

For peer review only - http://bmjopen.bmj.com/site/about/guidelines.xhtml

organizations, and the Ministry of Health and Child Care (MoHCC) to investigate MH gaps in the community, develop the MH intervention, and inform the research questions for this study.

To identify the most relevant MH gaps and needs in our intervention communities, we worked with key stakeholders to inform a Theory of Change (ToC) for the MH intervention. The ToC articulated the critical pathways to improve key MH outcomes among young women in Zimbabwe and guided the development of the MH intervention components. The intervention was then piloted from April to June 2019 and feedback from both healthcare providers and clients were used to refine and inform the scaled-up implementation of the MH intervention.

No patients were involved in the research process, design of the study, the recruitment and conduct of the study, or the dissemination of study results.

Results

Participant characteristics

318 female clients were screened at two of the CHIEDZA clusters (Hatcliffe and Budiriro) between December 2019 and February 2020. Of those screened, two were outside the eligible age range and 13 (4.1%) declined to participate due to time constraints. A total of 303 participants were therefore enrolled in the cohort study (Figure 1).

Sociodemographic characteristics of participants at each timepoint are presented in Table 2. Of the 303 participants at baseline, half (51.2%) were aged 16–19 years old and half (51.8%) had completed O-level education (equivalent to the UK GCSE). One-third (35.0%) of participants were in school and 87.8% did not have a job. Half (51.2%) obtained money or income from a relative and 28.2% obtained money from a partner. 61.1% of participants had never married and all came from low- or moderate-income households. Of the 303 participants that were seen at baseline, 189 (62.4%) were seen at six-month visits, and 184 (60.7%) at 12-month visits. Overall, cohort participant characteristics were broadly similar at baseline, six-month visits, and 12-month visits.

For peer review only

299 **Table 2 Sociodemographic characteristics and menstrual health outcomes at each time point**

Sociodemographic characteristics of MH Cohort Participants								
	BASELINE (N=303)		SIX-MONTH VISIT (N=189)			12-MONTH VISIT (N=184)		
	N	%	N	%	p-value	N	%	p-value
**Age**								
16 - 19	155	51.2%	84	54.3%	0.169	68	53.3%	0.002
20 -24	148	48.8%	105	45.7%		116	46.7%	
**Highest level of education**								
Less than primary school or primary school completed	110	36.3%	66	34.9%	0.001	55	29.9%	0.178
O-levels completed	157	51.8%	90	47.6%		97	52.7%	
More than O-levels completed	36	11.9%	33	17.5%		32	17.4%	
**Currently in school**								
Yes	106	35.0%	72	38.1%	0.485	61	33.2%	0.680
No	197	65.0%	117	61.9%		123	66.8%	
**Paying job**								
Yes	37	12.2%	20	10.6%	0.583	29	15.8%	0.267
No	266	87.8%	169	89.4%		155	84.2%	
**Marital status**								
Never married	185	61.1%	126	66.7%	0.271	114	61.9%	0.971
Married, or living as if married	110	36.3%	61	32.3%		64	34.8%	
Single (widowed or divorced)	8	2.6%	2	1.00%		6	3.3%	
**Religion**								
Not religious	8	2.6%	4	2.10%	0.808	3	1.6%	0.677

For peer review only - http://bmjopen.bmj.com/site/about/guidelines.xhtml

Christian	251	82.8%	154	81.5%		157	85.3%	
Other	44	14.5%	31	16.4%		24	13.1%	
**Household population**								
Lives alone	10	3.3%	0	0.0%	0.007	3	1.6%	0.026
Lives with 1-3 other people	116	38.3%	58	30.7%		55	29.9%	
Lives with 4-6 other people	147	48.5%	101	53.4%		93	50.5%	
Lives with >6 other people	30	9.9%	30	15.9%		33	17.9%	
**Total household income**								
Low-income	149	49.2%	93	49.2%	0.995	84	45.7%	0.451
Moderate-income	154	50.8%	96	50.8%		100	54.3%	
**Source of income[1]**								
I don't get money from anyone	59	19.6%	28	15.0%	0.525	35	19.4%	0.998
I get money from a relative	154	51.2%	105	56.5%		91	50.6%	
I get money from my partner	85	28.2%	52	28.0%		52	28.9%	
I get money from somewhere else	3	1.0%	1	0.5%		2	1.1%	

1 missing data for "Source of income" sociodemographic characteristics at baseline (N=303), six-month visit (N=189), and 12-month visit (N=184)

MH knowledge, perceptions, and practices

For MH knowledge, perceptions, and MH practices for reusable pads, the mean scores, and the proportion of participants with all correct or positive responses increased from baseline to six-month visit and decreased from six-month visit to 12-month visit (Table 3). For MH practices for menstrual cups, the mean score and proportion with all positive responses increased from baseline to six-month visit and from six-

For peer review only - http://bmjopen.bmj.com/site/about/guidelines.xhtml

month visit to 12-month visit. For MH knowledge and perceptions, there was strong evidence of an increase in mean scores from baseline to six-month visit with MH knowledge mean score reflecting the highest improvement (1.62; 95% CI:1.27-1.97) over time. There was also an increase in mean score from baseline to 12-month visit for MH perceptions.

Table 3: MH outcome mean scores (M) and odds ratios (ORs) for the proportion answering all items correctly over time for participants at six-month visit vs baseline, and 12-month visit vs baseline

	BASELINE (N=303)		SIX-MONTH VISIT (N=189)					12-MONTH VISIT (N=186)				
**MH Outcome**	**M**	**% with all correct/positive responses**	**M**	**% with all correct/positive responses**	**Absolute increase in mean score (95% CI)**	**aOR (95% CI)**	**p-value**	**M**	**% with all correct/positive responses**	**Absolute increase in mean score (95% CI)**	**aOR (95% CI)**	**p-value**
**Knowledge**	5.81	5.3%	7.45	42.3%	1.62 (1.27 – 1.97)	12.14 (6.77 – 21.76)	<0.001	6.92	32.6%	1.08 (0.73 – 1.42)	7.80 (4.3 – 14.16)	<0.001
**Perceptions**	2.54	9.2%	3.03	21.2%	0.49 (0.23 – 0.75)	2.85 (1.59 – 5.09)	<0.001	2.90	19.0%	0.36 (0.10 – 0.62)	2.53 (1.40 – 4.55)	<0.005
**Practices (reusable pads)**	4.41	14.2%	5.58	40.6%	1.17 (0.82 – 1.52)	4.68 (2.28 – 9.58)	<0.001	5.53	34.4%	0.96 (0.60 – 1.30)	3.64 (1.77 – 7.48)	<0.001
**Practices (menstrual cups)**	3.08	21.1%	3.80	40.6%	0.55 (0.40 – 1.15)	39.9 (0.05 – 34638.94)	<0.3	4.00	48.5%	0.95 (0.36 – 1.53)	1200.42 (0.44 – 3297620.00)	<0.1

respectively

**MH knowledge and MH practices (reusable pads) ORs adjusted for age and household population, MH perceptions OR adjusted for level of education, and MH practices (menstrual cups) OR adjusted for age*

After adjusting for confounders (age, level of education, and household population), there was strong evidence of an increase in MH knowledge, perceptions, and practices over time (Table 3). At six-month visit, participants were more likely to respond correctly or positively for all items for MH knowledge

For peer review only

For peer review only - http://bmjopen.bmj.com/site/about/guidelines.xhtml

adjusted odds ratio (aOR)=12.14; 95%CI:6.8-21.8), MH perceptions (aOR=2.85; 95%CI:1.6-5.1), and practices for reusable pads (aOR=4.68; 95%CI:2.3-9.6) than at baseline. Similarly, there was strong evidence of an improved response on MH knowledge (aOR=7.8; 95%CI:4.3–14.2), MH perceptions (aOR=2.53; 95%CI:1.4– 4.6), and MH practices (reusable pads) (aOR=3.64; 95%CI:1.8–7.5) from baseline to 12-month visit. However, there was a slight decrease in odds of improved responses between six-month and 12-month visits for MH knowledge, perceptions, and practices for reusable pads.

Qualitative findings provided details and explanations to the increases in knowledge, perceptions, and practices. They explained how an unmet need for MH education, support, and products facilitated uptake of the MH intervention:

> *"All I want to say is CHIEDZA has helped me so much. For me, I no longer have stress to include pads on my grocery list,"* (Hatcliffe, FGD, 20 – 24 years old).

MH knowledge

Participants reported acquiring MH knowledge through the intervention. When asked questions about menstruation in the FGDs after the intervention, menstrual pain management, and how to use, wash, dry, and store the reusable menstrual products given to them through the MH intervention, almost all of the participants responded confidently and accurately. For example, when asked about use of the menstrual cup and "virginity" they responded:

> *"They (the CHIEDZA healthcare providers) explained… They said that it's not bad and it does not affect someone's virginity. I believe that it doesn't affect virginity,"* (Budiriro, FGD, 16 – 19 years old).

Their responses evidenced that participants had both retained the MH education from the intervention and gained additional MH knowledge on "best practices" based on their experiences using the products. When asked about washing and drying the reusable pads, participants responded:

For peer review only

> *"Don't wear [the pads] when damp… That's when it smells bad. It must dry properly because it's made of cotton…"* (Budiriro, FGD, 16 – 19 years old).

While most of the conversation around MH knowledge revealed accurate information retention, some of the responses around menstrual cups and pain management highlighted how sociocultural norms and persistent myths overrode MH education in the intervention. For example, when asked about menstrual pain, one participant said:

> *"For me, when I was a girl, I didn't experience period pain. It came after birth. I think I got it from my hospital bed. I think the bed I used was used by someone who had period pain,"* (Budiriro, FGD, 20 – 24 years old).

MH perceptions

After the intervention, participants reported feeling more confident about knowing how to manage their menstruation using the reusable menstrual products. Many participants also reported feeling "*proud*" and more positive about their menstruation. They described the MH intervention as reducing the shame they felt about menstruation. Some participants stated that they were determined to defy sociocultural norms that framed menstruation as a shameful "personal secret" no one else should know about:

> *"As for me, at our homestead there are boys but it's that, I dry my things [reusable pads] outside on the washing line because those boys they know about menstruation. They will marry their wives and they will also go on their monthly periods, even their mothers who gave birth to them, they go on their periods, it's not something new…"* (Budiriro, FGD, 16 – 19 years old).

Despite the MH education and support, however, some still faced stigma and expressed feeling shame and anxiety around menstruation. Fear of "smelling like fish" or leaking menstrual blood persisted and informed MH product choice and practices. This shame around menstruation prevented them from feeling comfortable and confident during menstruation and when using and/or washing their menstrual products of choice:

For peer review only

For peer review only - http://bmjopen.bmj.com/site/about/guidelines.xhtml

"I thought it better to dry it [my pads] inside the house because people would want to know, what that is. They would also know about my period, when it started… so I will not feel alright," (Hatcliffe, FGD, 20 – 24 years old).

For some, longstanding sociocultural norms around menstruation overrode the positive menstrual perceptions gained through the intervention, and impacted MH practices, despite participants' gained knowledge.

"I stay with my aunt, my husband's sister. So, when I got the cup, she asked me if she can use the cup... The cup is not meant for girls, girls should use pads," (Hatcliffe, FGD, 20 – 24 years old).

MH practices

Participants were excited to have the opportunity to choose and use different types of menstrual products through the intervention. Product choice and menstrual practice was informed both by the knowledge gained through the intervention, as well as external sociocultural factors. Sociocultural beliefs around the menstrual cup causing the loss of "virginity" prevented many participants from using the cup, leading to low cup uptake. This was despite the issues of cup use and virginity being discussed during MH education sessions embedded in the MH intervention.

"Youths, they fear, and I'm one of them. I never attempted to use it because of fear, I was afraid to lose virginity, but I heard that if you use it, it's alright and you don't feel anything, everything will be normal," (Budiriro, FGD, 16 – 19 years old).

Since products were used away from the CHIEDZA intervention site, at home, the influence of sociocultural factors was strong.

"For me, I remember when we got these products, I was excited. When I got home, I showed my mom and she just said, 'I don't like that thing [the cup]'. So, you even become afraid of a thing which your mom does not approve of," (Budiriro, FGD, 20 – 24 years old).

For peer review only

While most participants chose not to use the menstrual cup, those that did, reported positive user experiences that encouraged them to continue cup use over time:

> *"I was scared at first to use the cup but when I started using it [chuckles]... It is very rare to see my pads on the washing line,"* (Hatcliffe, FGD, 20 -24 years old).

Environmental factors such as location (at home versus at work or school) and access to soap, water, and/or facilities to safely wash and dry reusable products also informed MH practices. These factors linked to the aforementioned perceptions of shame and anxiety around menstrual blood, with cleanliness felt to be a priority.

> *"There is a difference, if I'm at home and school, they are two different things. I don't use period pants when I'm at school because it does not handle blood for long time and the thing is you will be coming in and out in classroom… My colleagues will become suspicious. So, if I'm home I use period pants and when I'm school I use reusable pads."* (Budiriro, FGD, 16 – 19 years old).

> *"When travelling I prefer disposable pads because you never know where you go, you might not have water to use, and these things they need privacy, because when washing or drying them everyone there would be curious to know about it."* (Budiriro, FGD, 20 -24 years old).

For some, menstrual flow also informed MH practices, including using more than one menstrual product at a time.

> *"I can say first three days will be using a combination period pants and reusable pad. But last day will just use period pant."* (Budiriro, FGD, 16 – 19 years old).

Additional quotes from the FGDs are highlighted in Table 4.

Table 4 Summary of quotes from focus group discussion participants

MAIN THEMES	QUOTES FROM THE FOCUS GROUP DISCUSSIONS
MH KNOWLEDGE	*"When I clean them [reusable pads], I fetch water in a bucket, go to the bathroom, wash using green bar soap, rinse and put them on the washing line to dry, then put them in a bag and then in my drawer,"* (Hatcliffe, FGD, 20 – 24).

For peer review only - http://bmjopen.bmj.com/site/about/guidelines.xhtml

For peer review only

	"If you give birth, period pain goes away," (Hatcliffe, FGD, 20 – 24).<br><br>*"Don't wash them using detergents like washing powder, you should use green bar the dry them in sunlight for it to dry then you can you it again,"* (Hatcliffe, FGD, 16 – 19 years old).<br><br>*"We were told to boil the cup. It was said to destroy germs plus I think it can soften the hardness of it,"* (Budiriro, FGD, 20 -24 years old).
MH PERCEPTIONS	*"When I hear about CHIEDZA, I feel happy because I think of the products they provide for the girls in my community,"* (Hatcliffe, FGD, 16 – 19 years old).<br><br>*"I have difficulties when drying, at our homestead there are boys, so I dry my products inside our house…"* (Budiriro, FGD, 16 – 19 years old).<br><br>*"As for my mom, she said 'don't use that thing… Just wash other pads that you have.' She didn't want too much [more than that],"* (Budiriro, FGD, 16 -19 years old).<br><br>*"The disadvantages are people are not liking the cup especially girls they say that it makes then lose their virginity and some when they wear it, they feel like they are having sexual intercourse when walking of the tail,"* (Hatcliffe, FGD, 20 -24 years old).
MH PRACTICES	*"I can say girls with 16 - 19years are the ones who like reusable pads whilst the 20-24 they need cup,"* (Budiriro, FGD, 16 – 19 years old).<br><br>*"On my first days I use reusables then on my last days I used period pants because the blood flow will be minimal…"* (Hatcliffe, FGD, 16 – 19 years old).<br><br>*"[I wear] period pant first day of period because I will be having a light flow but other days, I will be using pads,"* (Budiriro, FGD, 16 – 19 years old).<br><br>*"When travelling I prefer disposable pads because you never know where you go, you might not have water to use, and these things they need privacy, because when drying them everyone there would be curious to know about it,"* (Budiriro, FGD, 20 – 24 years old.<br><br>*"Whenever I feel period pain, I take a pill,"* (Budiriro, FGD, 20 – 24 years old).

Discussion

This study provides evidence of improvements in key MH outcomes of knowledge, perceptions, and practices, through an intervention providing young women with MH products, education, and support in a community-based setting in urban Zimbabwe. The MH intervention led to knowledge acquisition on

For peer review only - http://bmjopen.bmj.com/site/about/guidelines.xhtml

menstruation, menstrual pain management, and menstrual product use; increased confidence in menstrual management; and improved menstrual health practices. Sociocultural and environmental factors, such as myths and taboos around menstruation, cultural beliefs, and access to WASH facilities at home, school, or work, also informed the MH outcomes, but were outside the intervention's scope, and may have attenuated the positive effects of the intervention over time.

Our baseline findings add to evidence highlighting poor MH knowledge, perceptions, and practices, among young women in LMICs (6, 21). With MH shrouded in secrecy and taboo, we supplement evidence that young women often reach menarche with limited information, and sometimes misinformation, compounded by a lack of access to MH pain relief and products of choice, leading to poor experiences of managing menstruation (10, 20, 37). We therefore emphasise the need for comprehensive MH interventions and provide evidence of an intervention to improve key MH outcomes.

The MH outcomes – knowledge, perceptions, and practices – were closely connected, highlighting the importance of integrated interventions targeting all three. Increased MH knowledge led to better MH practices and perceptions and better MH practices and perceptions led to better MH experiences overall. The value of offering choices of MH product enhanced MH experiences as young women could choose the product that best suited their circumstance and led to increased experiential knowledge on how to use, wash, dry, and store different products in young women's own specific environments. When encouraged to, participants were able to share their positive menstrual experiences and transfer that gained knowledge to others: changes in MH practices and perceptions thereby enabled MH information exchange and support (38). This process of acquiring and transferring MH knowledge and sharing menstrual experiences is often absent in most communities, without much needed MH discussion, correct information dissemination, and MH-related healthcare seeking behaviours (39-41).

The study highlights the influence of both the internal intervention components as well as external community social norms and access to WASH facilities at work, home, or school, as they interacted to

For peer review only - http://bmjopen.bmj.com/site/about/guidelines.xhtml

inform young women's menstrual experiences and MH outcome measures (19). This understanding of the multifaceted nature of MH is consistent with the growing understanding that MH plays a key role in the lived experiences of women across the globe. Comprehensive MH interventions must thus address biological, personal, interpersonal, environmental, and societal MH-related gaps and barriers (5).

The decrease in all three outcomes from six-month to 12-month visits albeit small, underscores the need for interventions that address social norms which can otherwise impede long-term MH improvements. Previous studies have similarly had limited impact on social norms around menstruation. In a study looking at the effect of a community-based MH intervention for adolescent girls in India (42), results showed a significant improvement in MH practices, but only a marginal decline in the social and religious restrictions that informed attitudes towards menstruation. While there may be additional factors that informed the 12-month visit dip in MH knowledge, perceptions, and practices in our study, such as participants' levels of engagement with MH activities within CHIEDZA, our results suggest the need for MH interventions that include community members and prioritise community sensitisation and education around MH-related issues if there are to be acceptable, effective, and sustainable over time.

This is one of the first MH studies to assess the effectiveness of a comprehensive community-based MH intervention that provided both MH "hardware", through menstrual products, and "software", through MH education, for young women in a community-based setting (16). Unlike most MH interventions, our study offered participants a choice of MH products and analgesics and used a mixed-methods approach to assess the effectiveness of this comprehensive MH intervention and the factors that informed MH product choice and patterns of use over time. Unusually, our study looks comprehensively at knowledge, perceptions, and practices together, and uses a validated measurement tool (MPNS-36) that has been used in other LMICs such as Uganda (19, 43).

In terms of limitations, there was substantial LTFU which may have led to bias. However, a comparison of participant sociodemographic characteristics at each visit showed no difference between those retained

For peer review only - http://bmjopen.bmj.com/site/about/guidelines.xhtml

in the study and those LTFU. Qualitative data may have been prone to recall bias due to being collected retrospectively, and social desirability bias, due to the sensitive nature of the discussions. While observed differences before and after the study were assumed to be due to the intervention, with the lack of a control group, other external factors, such as natural growth, ageing or maturity, or other environmental changes may have influenced MH outcomes (44). While this was further explored qualitatively, temporal bias may be present. The cohort participants were recruited in two randomly selected CHIEDZA intervention clusters (Hatcliffe and Budiriro) within one province (Harare) of Zimbabwe. Therefore, the relatively small number of participants in the cohort came from similar backgrounds and have similar characteristics making the study results less generalizable.

Conclusion

Overall, the study results showed that the comprehensive MH intervention was effective in improving MH knowledge levels, perceptions, and practices among young women. Provision of a combination of comprehensive MH education, analgesics, a choice of MH products, and support over time were key to intervention effectiveness and success. While exposure and access to the youth-friendly service provision of MH education and resources facilitated improvements in MH outcome measures, these were mediated by external and contextual factors such as sociocultural norms and environmental conditions. It is important that MH gaps and barriers are tackled using a holistic approach that frames MH as a health and human rights issue and engages with both individuals and the wider communities in which they operate.

For peer review only - http://bmjopen.bmj.com/site/about/guidelines.xhtml

List of Abbreviations

CHW	Community Health Worker
FGD	Focus Group Discussion
IDI	In-Depth Interview
LMIC	Low- and Middle-Income Countries
MH	Menstrual Health
MHH	Menstrual Health and Hygiene
MHM	Menstrual Hygiene Management
MoHCC	Ministry of Health and Child Care
NGO	Non-Governmental Organisation
OR	Odds Ratio
RA	Research assistant
SDGs	Sustainable Development Goals
SRH	Sexual and Reproductive Health
UNICEF	United Nations Children's Fund
WASH	Water, Sanitation, and Health
WHO	World Health Organization

For peer review only

Declarations

Ethics approval and consent to participate

This study was approved by the Medical Research Council of Zimbabwe [MRCZ/A/2387], the London School of Hygiene and Tropical Medicine ethics committee [16124/RR/11602] and the Biomedical Research and Training Institute Institutional Review Board (AP149/2018). Our study did not include participants under 16 years old and written informed consent was obtained from all participants above the age of 16 years old before the survey and FGDs were initiated. Pseudonyms were used during the FGDs and in quotations for confidentiality and anonymity. All recordings were downloaded on to a password-secured laptop for processing purposes and deleted once the data analyses were complete.

Consent for publication

Not applicable

Availability of data and materials

The datasets used and/or analysed during the current study are available from the corresponding author on request.

For peer review only

Authors' contributions

MT designed the menstrual health intervention, and collected, analysed, and interpreted the quantitative and qualitative data, and drafted the manuscript. CMY assisted in analysing the qualitative data and was a major contributor in writing the manuscript. HW assisted in analysing the quantitative data and was a major contributor in writing the manuscript. LL assisted in analysing the quantitative data. NG, PN, TN and PI assisted in the collection of the qualitative and quantitative data. TB and NR assisted with quantitative data collection and management. RAF, CDC, CVM, and ED contributed to the coordination of the study

and critical revisions of the manuscript. SF and JR contributed to design of the menstrual health intervention and the analysis plan. RAF is the Principal Investigator of the CHIEDZA Trial. All authors read and approved the final manuscript.

Competing interests

No competing interests.

Acknowledgements

The authors would like to thank all the participants in the study.

Funding

Swiss Agency for Development and Cooperation funding, the Wellcome Trust through a Senior Fellowship to RAF (206316/Z/17/Z), and the Fogarty International Center of the National Institutes of Health under Award Number D43 TW009539.

For peer review only

For peer review only - http://bmjopen.bmj.com/site/about/guidelines.xhtml

References

1. UNICEF. Guidance on Menstrual Health and Hygiene. www.unicef.org/wash: UNICEF, WASH; 2019 March.
2. Hennegan J, Winkler IT, Bobel C, Keiser D, Hampton J, Larsson G, et al. Menstrual health: a definition for policy, practice, and research. Sexual and Reproductive Health Matters. 2021;29(1):1911618.
3. Wilson LC, Rademacher KH, Rosenbaum J, Callahan RL, Nanda G, Fry S, et al. Seeking synergies: understanding the evidence that links menstrual health and sexual and reproductive health and rights. Sexual and Reproductive Health Matters. 2021;29(1):1882791.
4. Sommer M, Torondel B, Hennegan J, Phillips-Howard PA, Mahon T, Motivans A, et al. How addressing menstrual health and hygiene may enable progress across the Sustainable Development Goals. Global Health Action. 2021;14(1):1920315.
5. Hennegan J, Winkler IT, Bobel C, Keiser D, Hampton J, Larsson G, et al. Menstrual health: a definition for policy, practice, and research. Sexual and Reproductive Health Matters. 2021;29(1):31-8.
6. Hennegan J, Shannon AK, Rubli J, Schwab KJ, Melendez-Torres GJ. Women's and girls' experiences of menstruation in low- and middle-income countries: A systematic review and qualitative metasynthesis. PLoS Med. 2019;16(5):e1002803.
7. Hennegan J, Montgomery P. Do Menstrual Hygiene Management Interventions Improve Education and Psychosocial Outcomes for Women and Girls in Low and Middle Income Countries? A Systematic Review. PloS one. 2016;11(2):e0146985.
8. Sommer M, Caruso BA, Sahin M, Calderon T, Cavill S, Mahon T, et al. A Time for Global Action: Addressing Girls' Menstrual Hygiene Management Needs in Schools. PLoS Med. 2016;13(2):e1001962.
9. Sommer M, Schmitt M, Gruer C, Herbert A, Phillips-Howard P. Neglect of menarche and menstruation in the USA. Lancet. 2019;393(10188):2302.
10. Chandra-Mouli V, Patel SV. Mapping the knowledge and understanding of menarche, menstrual hygiene and menstrual health among adolescent girls in low- and middle-income countries. Reproductive Health. 2017;14(1):30.
11. Plesons M, Patkar A, Babb J, Balapitiya A, Carson F, Caruso BA, et al. The state of adolescent menstrual health in low- and middle-income countries and suggestions for future action and research. Reproductive Health. 2021;18(1):31.
12. VanLeeuwen C, Torondel B. Improving menstrual hygiene management in emergency contexts: literature review of current perspectives. Int J Womens Health. 2018;10:169-86.
13. Torondel B, Sinha S, Mohanty JR, Swain T, Sahoo P, Panda B, et al. Association between unhygienic menstrual management practices and prevalence of lower reproductive tract infections: a hospital-based cross-sectional study in Odisha, India. BMC infectious diseases. 2018;18(1):473-.
14. Phillips-Howard PA, Otieno G, Burmen B, Otieno F, Odongo F, Odour C, et al. Menstrual Needs and Associations with Sexual and Reproductive Risks in Rural Kenyan Females: A Cross-Sectional Behavioral Survey Linked with HIV Prevalence. J Womens Health (Larchmt). 2015;24(10):801-11.
15. Punzi MC. Technical Brief.
16. Hennegan J. Interventions to Improve Menstrual Health in Low- and Middle-Income Countries: Do We Know What Works? In: Bobel C, Winkler IT, Fahs B, Hasson KA, Kissling EA, Roberts T-A, editors. The Palgrave Handbook of Critical Menstruation Studies. Singapore: Springer Singapore; 2020. p. 637-52.
17. Evans RL, Harris B, Onuegbu C, Griffiths F. Systematic review of educational interventions to improve the menstrual health of young adolescent girls. BMJ Open. 2022;12(6):e057204.
18. Kuhlmann AS, Henry K, Wall LL. Menstrual Hygiene Management in Resource-Poor Countries. Obstetrical & Gynecological Survey. 2017;72(6):356-76.

For peer review only

19. Hennegan J, Nansubuga A, Smith C, Redshaw M, Akullo A, Schwab KJ. Measuring menstrual hygiene experience: development and validation of the Menstrual Practice Needs Scale (MPNS-36) in Soroti, Uganda. BMJ Open. 2020;10(2):e034461.

20. Tembo M, Renju J, Weiss HA, Dauya E, Bandason T, Dziva-Chikwari C, et al. Menstrual product choice and uptake among young women in Zimbabwe: a pilot study. Pilot Feasibility Stud. 2020;6(1):182.

21. UNICEF/Zimstats. Zimbabwe 2019 MICS Suvery Findings Report. 2019.

22. Organisation SND. Girls in control: Compiled findings from studies on menstrual hygiene management of school girls. Ethopia, South Sudan, Tanzania, Uganda, and Zimbabwe. SNV Netherlands Development Organisation: The Hague; 2015.

23. Tamiru S, Mamo K, Acidria P, Mushi R, Satya Ali C, Ndebele L. Towards a sustainable solution for school menstrual hygiene management: cases of Ethiopia, Uganda, South-Sudan, Tanzania, and Zimbabwe. Waterlines. 2015;34(1):92-102.

24. Ssewanyana D, Bitanihirwe BKY. Menstrual hygiene management among adolescent girls in sub-Saharan Africa. Glob Health Promot. 2019;26(1):105-8.

25. Ndlovu E, Bhala E. Menstrual hygiene - A salient hazard in rural schools: A case of Masvingo district of Zimbabwe. Jamba. 2016;8(2):204.

26. Dziva Chikwari C, Dauya E, Bandason T, Tembo M, Mavodza C, Simms V, et al. The impact of community-based integrated HIV and sexual and reproductive health services for youth on population-level HIV viral load and sexually transmitted infections in Zimbabwe: protocol for the CHIEDZA cluster-randomised trial [version 1; peer review: awaiting peer review]. Wellcome Open Research. 2022;7(54).

27. Kansiime C, Hytti L, Nalugya R, Nakuya K, Namirembe P, Nakalema S, et al. Menstrual health intervention and school attendance in Uganda (MENISCUS-2): a pilot intervention study. BMJ Open. 2020;10(2):e031182.

28. Endler M, Al-Haidari T, Benedetto C, Chowdhury S, Christilaw J, El Kak F, et al. How the coronavirus disease 2019 pandemic is impacting sexual and reproductive health and rights and response: Results from a global survey of providers, researchers, and policy-makers. Acta Obstetricia et Gynecologica Scandinavica.n/a(n/a).

29. Mackworth-Young CR, Chingono R, Mavodza C, McHugh G, Tembo M, Chikwari CD, et al. Community perspectives on the COVID-19 response, Zimbabwe. Bull World Health Organ. 2021;99(2):85-91.

30. Dzawanda B, Matsa M, Nicolau M. Poverty on the rise: The impact of the COVID-19 lockdown on the informal sector of Gweru, Zimbabwe. Int Soc Sci J. 2021:10.1111/issj.12285.

31. Boynton PM, Greenhalgh T. Selecting, designing, and developing your questionnaire. BMJ. 2004;328(7451):1312-5.

32. Braun V, Clarke V. Using thematic analysis in psychology. Qualitative Research in Psychology. 2006;3(2):77-101.

33. Fereday J, Muir-Cochrane E. Demonstrating Rigor Using Thematic Analysis: A Hybrid Approach of Inductive and Deductive Coding and Theme Development. International Journal of Qualitative Methods. 2006;5(1):80-92.

34. King N. Using templates in the thematic analysis of text. 2004 2021/04/08. In: Essential Guide to Qualitative Methods in Organizational Research [Internet]. London: SAGE Publications Ltd; [256-70]. Available from: http://sk.sagepub.com/books/essential-guide-to-qualitative-methods-in-organizational-research.

35. Mackworth-Young C, Dringus S, Dauya E, Chikwari C, Mavodza C, Tembo M, et al. Putting youth at the centre: co-design of a community-based intervention to improve HIV outcomes among youth in Zimbabwe. Wellcome Open Research. 2022;7:53.

For peer review only - http://bmjopen.bmj.com/site/about/guidelines.xhtml

36. Tembo M, Renju J, Weiss HA, Dauya E, Bandason T, Dziva-Chikwari C, et al. Menstrual product choice and uptake among young women in Zimbabwe: a pilot study. Pilot and Feasibility Studies. 2020;6(1):182.

37. Shanbhag D, Shilpa R, D'Souza N, Josephine P, Singh J, Goud B. Perceptions regarding menstruation and practices during menstrual cycles among high school going adolescent girls in resource limited settings around Bangalore city, Karnataka, India. International Journal of Collaborative Research on Internal Medicine & Public Health. 2012;4(7):1353.

38. Hennegan J, Dolan C, Steinfield L, Montgomery P. A qualitative understanding of the effects of reusable sanitary pads and puberty education: implications for future research and practice. Reprod Health. 2017;14(1):78.

39. McMahon SA, Winch PJ, Caruso BA, Obure AF, Ogutu EA, Ochari IA, et al. 'The girl with her period is the one to hang her head' Reflections on menstrual management among schoolgirls in rural Kenya. BMC Int Health Hum Rights. 2011;11:7.

40. Mahajan T. Imperfect Information in Menstrual Health and the Role of Informed Choice. Indian Journal of Gender Studies. 2019;26(1-2):59-78.

41. Chiou M-H, Wang H-H, Yang Y-H. Effect of Systematic Menstrual Health Education on Dysmenorrheic Female Adolescents' Knowledge, Attitudes, and Self-Care Behavior. The Kaohsiung journal of medical sciences. 2007;23:183-90.

42. A.R. Dongre PRD, Garg BS. The Effect of Community-Based Health Education Intervention on Management of Menstrual Hygiene among Rural Indian Adolescent Girls. World Health & Population. 2007;9(3):48-54.

43. Hennegan J, Brooks DJ, Schwab KJ, Melendez-Torres GJ. Measurement in the study of menstrual health and hygiene: A systematic review and audit. PloS one. 2020;15(6):e0232935.

44. Ho AMH, Phelan R, Mizubuti GB, Murdoch JAC, Wickett S, Ho AK, et al. Bias in Before–After Studies: Narrative Overview for Anesthesiologists. Anesthesia & Analgesia. 2018;126(5):1755-62.

For peer review only

Tables and Figures

Figure 1: Screening, recruitment, and follow-up flowchart for MH cohort study

Table 1: Questionnaire questions selected to measure menstrual health outcomes in the study

Table 2 : Sociodemographic characteristics and menstrual health outcomes at each time point

Table 3: MH outcome mean scores (M) and odds ratios (ORs) over time for participants at six-month visit vs baseline, and 12-month visit vs baseline respectively

Table 4: Summary of quotes from FGD participants

Additional Files

File Name: Appendix A

Format:.pdf

Title: Quantitative Questionnaire

Description: Complete quantitative questionnaire used in the MH cohort study

For peer review only

For peer review only - http://bmjopen.bmj.com/site/about/guidelines.xhtml

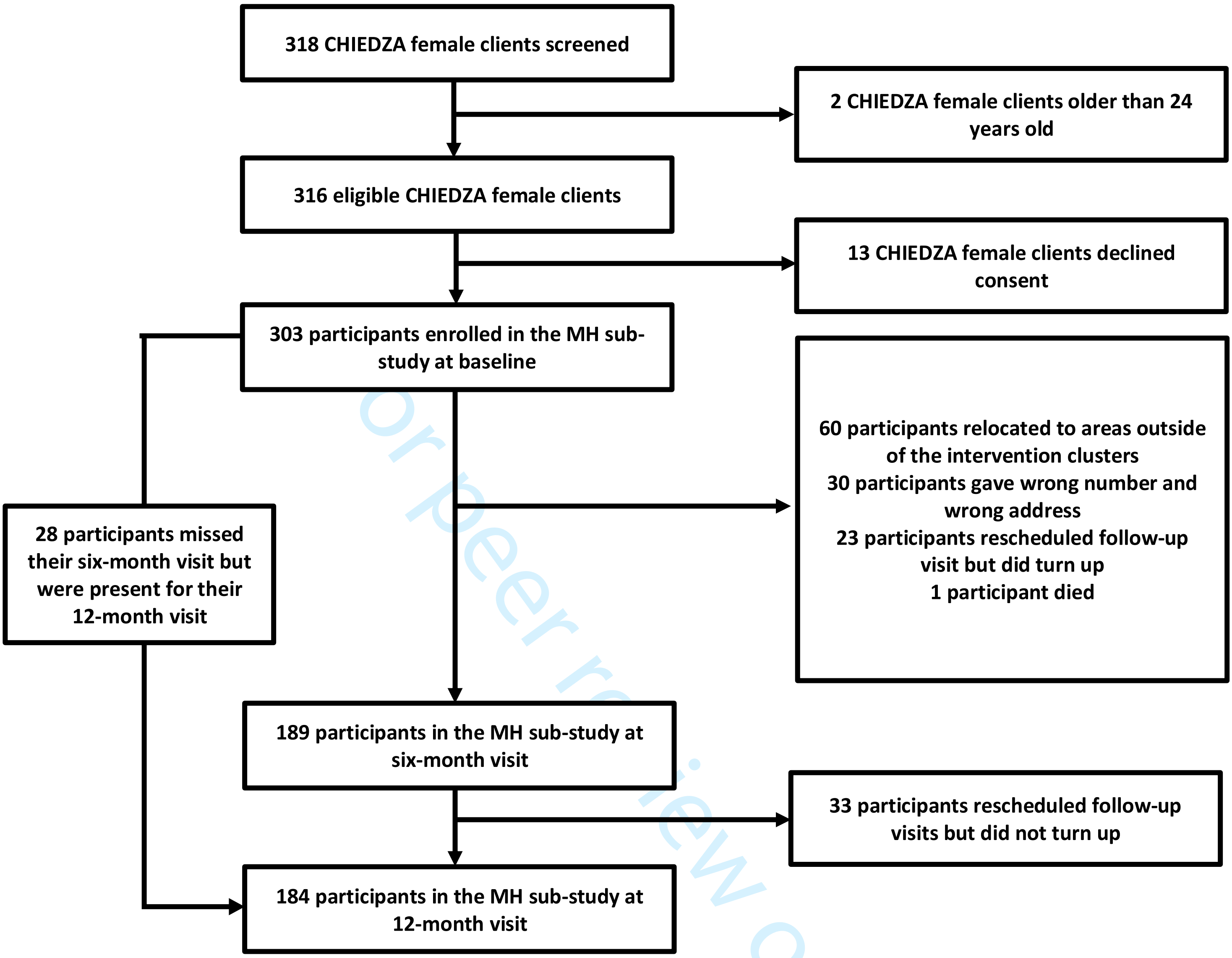

Figure 1 Screening, recruitment, and follow-up flow chart for MH cohort sub-study

For peer review only

**MH STUDY ADMINISTRATION**<br>**This section is to be filled out by the attending staff member**			
**A00**	*STAFFNO*	Staff Initials	☐☐☐
**A01**	*STUDYNO*	Study Participant Cohort Number	☐☐☐☐☐☐
**A02**	*DOB*	Date of Birth (dd/mm/yyyy)	☐☐/☐☐/☐☐☐☐
**A03**	*AGE*		☐☐
**A04**	*DATE*	Date of visit (dd/mm/yyyy)	☐☐/☐☐/20☐☐
**A05**	*VISIT*	Type of visit	Initial ☐ 6m☐ 12m☐

This questionnaire is to be filled out by participants in the menstrual health study

The purpose of this questionnaire is to collect information about you and how you feel and behave during your period.

There are 9 parts to this questionnaire. It should not take more than 30 minutes of your time. Please read the questions carefully and fill in the questionnaire as honestly as possible.

Thank you for your participation.

PART I: SOCIODEMOGRAPHIC INFORMATION

These questions are to help understand more about your life

**A06**	*EDU*	Highest Level of Education	Please tick your highest level of education:<br>No Formal School Education ☐<br>Incomplete Primary School ☐<br>Complete Primary School ☐<br>Incomplete High School Education ☐<br>Complete High School Education (O'Levels) ☐<br>Complete High School Education (A'Levels) ☐<br>Incomplete Tertiary Education ☐

For peer review only

			Complete University ☐
**A07**	*JOB*	Do you have a paying job?	Yes ☐ No ☐
**A08**	*MONEY*	If you do not work, where do you get your money	I do not get money from anyone ☐<br>I get money from a relative in the household (mother/ father/brother/sister etc.) ☐<br>I get money from a relative outside the household ☐<br>I get money from my partner ☐<br>Other ☐<br>If you chose "Other", please list here: ___________
**A09**	*MSTAT*	Marital Status	Single (Never Married) ☐<br>Married, or living as if you are married ☐<br>Widowed ☐<br>Divorced/Separated ☐
**A10**	*RES*	Residence	Urban ☐<br>Peri-Urban ☐<br>Rural ☐
**A11**	*RELGN*	Religion	Not Religious ☐<br>Traditional ☐<br>Christian (Pentecostal) ☐<br>Christian (Protestant) ☐<br>Roman Catholic ☐<br>Other ☐

For peer review only

			If you chose "Other", please list here: ___________
**A12**	*HH*	Please tick any items your household has:	Please tick all that apply:<br>Television ☐<br>Radio ☐<br>Mobile Phone ☐<br>Electricity ☐<br>Water source inside the house ☐<br>Latrine inside the house ☐
**A13**	*INC*	How many people live in your household with you:	None (I live alone) ☐<br>1 – 3 other people ☐<br>4 – 6 other people ☐<br>More than 6 people ☐
**PART II: GENERAL QUESTIONS ABOUT YOURSELF**<br>**These questions are about you and why you came to the CHIEDZA site**			
**A14**	*CHIEDZA 1*	What brought you to CHIEDZA today?	Please tick all that apply:<br>STI Treatment ☐<br>Menstrual Hygiene Management ☐<br>HIV Testing ☐<br>HIV Treatment ☐<br>Condoms ☐<br>Family Planning ☐<br>Counselling ☐

For peer review only

			Other ☐<br>If you chose "Other", please list here: ___________
**A15**	*CHIEDZA 2*	Would you come to CHIEDZA if you could not receive MH products and information?	Yes ☐ No ☐
**PART III: PERIOD and PUBERTY KNOWLEDGE**<br>**These questions are about your understanding of puberty and periods**			
**QUESTIONS ABOUT PUBERTY AND MENSTRUATION**			
**A16**	*KNW1*	Changes in the body during puberty happen because of hormones	Yes ☐ No ☐ I don't know ☐
**A17**	*KNW2*	Puberty continues throughout a girl's life	Yes ☐ No ☐ I don't know ☐
**A18**	*KNW2*	Menstruation in girls and women is normal	Yes ☐ No ☐ I don't know ☐
**A19**	*KNW3*	When a girl gets her first menstrual period, her body is able to carry a child	Yes ☐ No ☐ I don't know ☐
**A20**	*KNW4*	Menstrual blood is caused by the breakdown of the lining of the womb	Yes ☐ No ☐ I don't know ☐
**A21**	*KNW5*	It is normal to have irregular periods as a teenager	Yes ☐ No ☐ I don't know ☐
**A22**	*KNW6*	Menstruation continues throughout a girl's life	Yes ☐ No ☐ I don't know ☐
**A23**	*KNW7*	A period normally lasts 2 days or less	Yes ☐ No ☐ I don't know ☐
**A24**	*KNW8*	Period products that are inserted into the vagina	Yes ☐ No ☐ I don't know ☐

For peer review only

		(such as tampons and the menstrual cup) affect your virginity	
**PART IV: QUESTIONS ABOUT YOUR PERIOD HISTORY**<br>**The following questions are about you and your menstrual periods**			
The following questions are about your period history			
**A25**	*MENARCHE*	How old were you when you first started your periods?	Age ☐☐<br>I don't remember ☐
**A26**	*TAUGHT*	Who first told you or taught you about periods	No one told me about periods ☐<br>My mother ☐<br>My father ☐<br>My female/male friend ☐<br>My teacher ☐<br>Other ☐<br>If you chose "Other", please list here: ___________
**A27**	*INFO1*	Do you feel you had enough information and education to manage your period when you got your first period?	Yes ☐<br>Somewhat ☐<br>Not Really ☐<br>Definitely No ☐
**A28**	*INFO2*	What did you think was happening when you got your first period?	Curse ☐<br>Disease ☐<br>Injury ☐<br>Normal function for young women ☐<br>I did not know what was happening ☐

For peer review only

			Other ☐<br>If you chose "Other", please list here: ___________
**A29**	*DAYS*	On average, how many days do you usually bleed for during your periods?	1 – 3 days ☐<br>4 – 7 days ☐<br>More than 7 days ☐
**A30**	*RCNTD AYS*	How many days did you bleed during your most recent period?	☐☐<br>I Do Not Remember ☐
**PART V: QUESTIONS ABOUT PAIN MANAGEMENT**<br>**The following questions are about menstruation-related pain**			
**A31**	*PAINK NW1*	Painkillers can be used to relieve menstrual pain	Yes ☐ No ☐ I don't know ☐
**A32**	*PAINK NW2*	Painkillers can cause problems having children (barrenness)	Yes ☐ No ☐ I don't know ☐
**A33**	*PAINK NW3*	Exercising, stretching, or putting a warm towel or bottle on your stomach can relieve menstrual pain	Yes ☐ No ☐ I don't know ☐
During my last (most recent) menstrual period…			
**A34**	*PAIN1*	I experienced period-related pain in my lower back and/or abdomen	Yes ☐ No ☐
**A35**	*PAIN2*	I missed work or school due to period-related pain	Yes ☐ No ☐
**PART VI:**<br>**QUESTIONS ABOUT PRACTICES AND PERCEPTIONS DURING MENSTRUATION**<br>**The following questions are about how you manage your menstruation**			
The following questions are about your last (most recent) period			

For peer review only

**A36**	*PRDCT*	During your most recent period, what material(s)did you mostly use to collect or absorb your period blood?	Please tick all that apply:<br>Cloth/Rags/Fabric ☐<br>Reusable Pads ☐<br>Disposable Pads ☐<br>Menstrual Cup ☐<br>Period Pants ☐<br>Other ☐<br>Not Applicable ☐<br>If you chose "Other", please list here: ___________
**A37**	*HPRDCT*	During your most recent period, what material(s)did you mostly use to collect or absorb your period blood when at home?	Please tick all that apply:<br>Cloth/Rags/Fabric ☐<br>Reusable Pads ☐<br>Disposable Pads ☐<br>Menstrual Cup ☐<br>Period Pants ☐<br>Other ☐<br>Not Applicable ☐<br>If you chose "Other", please list here: ___________
**A38**	*SWPRDCT*	During your most recent period, what material(s)did you mostly use to collect or absorb your period blood when at school/work?	Please tick all that apply:<br>Cloth/Rags/Fabric ☐<br>Reusable Pads ☐<br>Disposable Pads ☐<br>Menstrual Cup ☐<br>Period Pants ☐

For peer review only

			Other ☐<br>Not Applicable ☐<br>If you chose "Other", please list here: ___________
During my last (most recent) menstrual period…			
**A39**	*PRDCT 1*	My menstrual products were comfortable	Strongly Disagree ☐<br>Disagree ☐<br>Agree ☐<br>Strongly Agree ☐
**A40**	*PRDCT 2*	I had enough of my menstrual products to change them as often as I wanted to	Strongly Disagree ☐<br>Disagree ☐<br>Agree ☐<br>Strongly Agree ☐
**A41**	*PRDCT 3*	Before I used them, I was satisfied with the cleanliness of my menstrual products	Strongly Disagree ☐<br>Disagree ☐<br>Agree ☐<br>Strongly Agree ☐
**A42**	*PRDCT 4*	I could get more of my menstrual materials when I needed to	Strongly Disagree ☐<br>Disagree ☐<br>Agree ☐<br>Strongly Agree ☐
**A43**	*PRDCT 5*	I was worried that my menstrual products would allow blood to pass through my outer garments	Strongly Disagree ☐<br>Disagree ☐<br>Agree ☐<br>Strongly Agree ☐
**A44**	*PRDCT 6*	I was worried that my menstrual products would	Strongly Disagree ☐<br>Disagree ☐

For peer review only

		move from place while I was wearing them	Agree ☐<br>Strongly Agree ☐
**A45**	*PRDCT 7*	I worried about how I would get more of my menstrual material if I ran out	Strongly Disagree ☐<br>Disagree ☐<br>Agree ☐<br>Strongly Agree ☐
**A46**	*PRDCT 8*	I felt comfortable carrying spare menstrual products with me outside my home	Strongly Disagree ☐<br>Disagree ☐<br>Agree ☐<br>Strongly Agree ☐
**A47**	*PRDCT 9*	I felt comfortable carrying menstrual products to the place where I changed them	Strongly Disagree ☐<br>Disagree ☐<br>Agree ☐<br>Strongly Agree ☐
**A48**	*PRDCT 10*	I felt comfortable storing my menstrual products until my next period	Strongly Disagree ☐<br>Disagree ☐<br>Agree ☐<br>Strongly Agree ☐
**A49**	*PRDCT 11*	I was able to wash my hands when I wanted to	Strongly Disagree ☐<br>Disagree ☐<br>Agree ☐<br>Strongly Agree ☐
**If participant ticks "Disposable Pads" or "Cloth/Rags/Fabric"**			
During my last (most recent) period…			
**B1**	*DIS1*	I was able to immediately dispose of my used menstrual material	Strongly Disagree ☐<br>Disagree ☐<br>Agree ☐

For peer review only

			Strongly Agree ☐
**B2**	*DIS2*	I was able to dispose of my used menstrual material in the way I wanted to	Strongly Disagree ☐<br>Disagree ☐<br>Agree ☐<br>Strongly Agree ☐
**B3**	*DIS3*	I worried about where to dispose of my used menstrual materials	Strongly Disagree ☐<br>Disagree ☐<br>Agree ☐<br>Strongly Agree ☐
**B4**	*DIS4*	I was concerned that others would see my menstrual materials in the place I disposed them	Strongly Disagree ☐<br>Disagree ☐<br>Agree ☐<br>Strongly Agree ☐
**If participant ticks "Cloth/Rags/Fabric", "Period Pants" and/or "Reusable Pads":**			
During my last (most recent) period…			
**C1**	*REUS1*	I had enough water to soak or wash my menstrual products	Strongly Disagree ☐<br>Disagree ☐<br>Agree ☐<br>Strongly Agree ☐
**C2**	*REUS2*	I had access to a basin or dish to soak or wash my menstrual products whenever I needed	Strongly Disagree ☐<br>Disagree ☐<br>Agree ☐<br>Strongly Agree ☐
**C3**	*REUS3*	I was able to wash my menstrual products when I wanted to	Strongly Disagree ☐<br>Disagree ☐<br>Agree ☐<br>Strongly Agree ☐

For peer review only

**C4**	*REUS4*	I had enough soap to wash my menstrual products	Strongly Disagree ☐<br>Disagree ☐<br>Agree ☐<br>Strongly Agree ☐
**C5**	*REUS5*	I was able to dry my products when I wanted to	Strongly Disagree ☐<br>Disagree ☐<br>Agree ☐<br>Strongly Agree ☐
**C6**	*REUS6*	I was worried that someone would see me while I was washing my menstrual products	Strongly Disagree ☐<br>Disagree ☐<br>Agree ☐<br>Strongly Agree ☐
**C7**	*REUS7*	I was worried that my menstrual products would not be dry when I needed them	Strongly Disagree ☐<br>Disagree ☐<br>Agree ☐<br>Strongly Agree ☐
**C8**	*REUS8*	I was worried that others would see my menstrual products while they were drying	Strongly Disagree ☐<br>Disagree ☐<br>Agree ☐<br>Strongly Agree ☐
**C9**	*REUS9*	How often did you change your menstrual products during the heaviest day of your most recent period?	Once a day (within a 24hr period) ☐<br>Twice a day (within a 24hr period) ☐<br>Three times a day (within a 24hr period) ☐<br>More than three times a day (within a 24hr period) ☐
**C10**	*REUS10*	At home, where did you most often change your menstrual products during your most recent period?	In a toilet inside the building ☐<br>In a toilet outside the building ☐<br>In a private room ☐<br>Outside (in the field/by the river) ☐

For peer review only

			Other ☐<br>If you chose "Other", please explain: ___________
**C11**	*REUS11*	At school/work, where did you most often change your menstrual products during your most recent period?	In a toilet inside the building ☐<br>In a toilet inside the building ☐<br>In a toilet outside the building ☐<br>In a private room ☐<br>Outside (in the field/by the river) ☐<br>Other ☐<br>Not applicable ☐<br>If you chose "Other", please explain: ___________
**C12**	*REUS12*	Where did you wash your menstrual products during your last period?	In the bathroom ☐<br>At a private well or tap in the house ☐<br>At a public well or tap outside the house ☐<br>In a pond or river ☐<br>Did not wash your menstrual products ☐<br>Other ☐<br>If you chose "Other", please explain: ___________
**C13**	*REUS13*	What did you use to wash your products with during your most recent period?	With water only ☐<br>With soap and water ☐<br>Did not wash my menstrual products ☐<br>Other ☐<br>If you chose "Other", please explain: ___________
**C14**	*REUS14*	Where did you dry your menstrual products during your most recent period?	Outside in the sun ☐<br>Outside in the sun, under a cloth ☐<br>Inside in the house in an open space ☐

For peer review only

			Inside the house in a closed space ☐<br>Did not dry my reusable pad, period pants, or cloth/rags/fabric ☐<br>Other ☐<br>If you chose "Other", please explain: ___________
**C15**	*REUS15*	Did you ever wear your menstrual product when it was damp (not yet dry) during your most recent period?	Yes ☐ (No ☐<br>Do Not Remember ☐
**C16**	*REUS16*	Where did you store your menstrual products after your most recent period?	In a cupboard ☐<br>In the toilet ☐<br>In an open space ☐<br>In a hidden place ☐<br>Did not store ☐<br>Other ☐<br>If you chose "Other", please explain: ___________
**If participant ticks "Menstrual Cup":**			
During my last (most recent) period…			
**D1**	*MC1*	I was able to sterilize my menstrual cup <u>when</u> I wanted after my period	Strongly Disagree ☐<br>Disagree ☐<br>Agree ☐<br>Strongly Agree ☐
**D2**	*MC2*	I was able to rinse my menstrual cup <u>when</u> I wanted	Strongly Disagree ☐<br>Disagree ☐<br>Agree ☐<br>Strongly Agree ☐

For peer review only

**D3**	*MC3*	I had enough water to rinse my menstrual cup	Strongly Disagree ☐<br>Disagree ☐<br>Agree ☐<br>Strongly Agree ☐
**D4**	*MC4*	I was worried that someone would see me while I was rinsing my menstrual cup	Strongly Disagree ☐<br>Disagree ☐<br>Agree ☐<br>Strongly Agree ☐
**D5**	*MC5*	I was worried that someone would see me while I was sterilizing my menstrual cup	Strongly Disagree ☐<br>Disagree ☐<br>Agree ☐<br>Strongly Agree ☐
**D6**	*MC6*	How long did it take you to get comfortable and relaxed using your menstrual cup?	1 Period ☐<br>2 Periods ☐<br>3 Periods ☐<br>4 or More Periods ☐<br>Still Not Comfortable or Relaxed ☐
**D7**	*MC7*	What is difficult about using your menstrual cup?	Tick All that Apply:<br>Could not get the cup in ☐<br>Too much pain and discomfort ☐<br>Difficult to clean ☐<br>Difficult to remove ☐<br>Other ☐<br>If you chose "Other", please explain: ___________
**D8**	*MC8*	How often did you empty and re-insert your menstrual cup during the heaviest day of your most recent period?	Once a day (within a 24hr period) ☐<br>Twice a day (within a 24hr period) ☐<br>Three times a day (within a 24hr period) ☐<br>More than three times a day (within a 24hr period) ☐

For peer review only

**D9**	*MC9*	At home, where did you most often empty and re-insert your menstrual cup when you needed to during your most recent period?	In a toilet inside the building ☐<br>In a toilet outside the building ☐<br>Outside (in the field/by the river) ☐<br>Other ☐<br>If you chose "Other", please explain: ___________
**D10**	*MC10*	At school/work, where did you most often empty and re-insert your menstrual cup when you needed to during your most recent period?	In a toilet inside the building ☐<br>In a toilet outside the building ☐<br>Outside (in the field/by the river) ☐<br>Not Applicable ☐<br>Other ☐<br>If you chose "Other", please explain: ___________
**D11**	*MC11*	Where did you rinse your menstrual cup during your last period?	In a private bathroom ☐<br>In a public bathroom ☐<br>At a private well or tap in the house ☐<br>At a public well or tap outside the house ☐<br>In a pond or river ☐<br>Did not rinse my menstrual cup ☐<br>Other ☐<br>If you chose "Other", please explain: ___________
**D12**	*MC12*	What did you rinse your menstrual cup with during your most recent period?	With water only ☐<br>With soap and water ☐<br>Did not rinse my menstrual cup ☐<br>Other ☐<br>If you chose "Other", please explain: ___________

For peer review only

**D13**	*MC13*	During your most recent period, how did you sterilise your menstrual cup?	Boiled cup for 3 min ☐<br>Washed with boiling water ☐<br>Soaked in vinegar and water solution ☐<br>Other ☐<br>If you chose "Other", please explain: ___________
**D14**	*MC14*	Did you store your menstrual cup in an aerated bag between periods?	Yes ☐ No ☐
**D15**	*MC15*	During your most recent period, did you ever use a reusable or disposable pad along with the menstrual cup?	Yes ☐ No ☐
**PART V: QUESTIONS ABOUT YOUR WASHING PRACTICES**<br>**The following questions are about how you wash your body and your vagina during your last (most recent) period**			
**A50**	*WASH1*	What type of washing did you do when you were on your most recent period?	External genital wash only ☐<br>Full body wash ☐<br>I did not wash myself ☐<br>Other ☐<br>If you chose "Other", please explain: ___________
**A51**	*WASH2*	On average, how many times did you wash your body during your most recent period?	Less than once a day ☐<br>Once a day ☐<br>Twice a day ☐<br>Three times a day or more ☐
**A52**	*WASH3*	What did you use to wash yourself during your most recent period?	Water only ☐<br>Soap and water ☐<br>Not Applicable ☐<br>Other ☐<br>If you chose "Other", please explain: ___________
**PART VII: QUESTIONS ABOUT YOUR ATTITUDES ABOUT MENSTRUATION**<br>**These questions are about how feel about menstruation**			

For peer review only

**A53**	*ATTITUDE1*	I feel dirty or impure during my menstrual period	Strongly Disagree☐<br>Disagree☐<br>Neutral☐<br>Agree☐<br>Strongly Agree☐
**A54**	*ATTITUDE2*	I feel like I can talk to friends about menstruation	Strongly Disagree☐<br>Disagree☐<br>Neutral☐<br>Agree☐<br>Strongly Agree☐
**A55**	*ATTITUDE3*	I feel like I can talk to my family members about menstruation	Strongly Disagree☐<br>Disagree☐<br>Neutral☐<br>Agree☐<br>Strongly Agree☐
**A56**	*ATTITUDE4*	I feel proud that I have my periods	Strongly Disagree☐<br>Disagree☐<br>Neutral☐<br>Agree☐<br>Strongly Agree☐
**A57**		It is important that I keep my period a secret from everyone	Strongly Disagree☐<br>Disagree☐<br>Neutral☐<br>Agree☐<br>Strongly Agree☐
**A58**	*ATTITUDE5*	I worry that boys will tease me about my period	Strongly Disagree☐<br>Disagree☐<br>Neutral☐

For peer review only

			Agree ☐<br>Strongly Agree ☐
**A59**	*ATTITUDE6*	I am anxious about my next period	Strongly Disagree ☐<br>Disagree ☐<br>Neutral ☐<br>Agree ☐<br>Strongly Agree ☐
**PART VIII: PARTICIPATION**<br>**The following questions are about how your period may affect your everyday activities**			
**A60**	*PRTCPTN1*	In the last 3 months, have you missed work/school because of your period?	Yes ☐ No ☐<br>**If "No", skip next question**
**A61**	*PRTCPTN2*	Why did you miss work/school?	Please tick all that apply:<br>Pain ☐<br>Nothing To Use To Manage My Period ☐<br>No place to change or wash ☐<br>Ashamed or embarrassed ☐<br>Scared I will leak ☐<br>Told to go home/Not allowed ☐
**A62**	*PRTCPTN3*	In the last 3 months, have you missed social events or community/religious happenings because of your period?	Yes ☐ No ☐<br>**If "No", skip next question**
**A63**	*PRTCPTN4*	Why did you miss the social events or social/religious happenings?	Please tick all that apply:<br>Pain ☐<br>Nothing To Use To Manage My Period ☐<br>No place to change or wash ☐<br>Ashamed or embarrassed ☐

For peer review only

			Scared I will leak ☐<br>Told to go home/Not allowed ☐
**A64**	*PRTCPT N5*	In the last 3 months, what everyday activities have you missed out on because of your period?	Please tick all that apply:<br>Household activities (washing clothes, dishes, etc) ☐<br>Sports (athletics, running, swimming, etc) ☐<br>Income-generating activities ☐<br>Religious activities ☐<br>Socialising with friends and family ☐<br>School ☐
**PART IX: FACTORS AT HOME**<br>**The following questions are about the surroundings in which you manage your period at home**			
At home during my last (most recent) period...			
**A65**	*FHOME 1*	I was able to change my menstrual products when I wanted to	Strongly Disagree ☐<br>Disagree ☐<br>Agree ☐<br>Strongly Agree ☐
**A66**	*FHOME 2*	I was satisfied with the place I used to change my menstrual products	Strongly Disagree ☐<br>Disagree ☐<br>Agree ☐<br>Strongly Agree ☐
**A67**	*FHOME 3*	I had a clean place to change my menstrual products	Strongly Disagree ☐<br>Disagree ☐<br>Agree ☐<br>Strongly Agree ☐
**A68**	*FHOME 4*	I was worried I would not be able to change my menstrual products when I needed to	Strongly Disagree ☐<br>Disagree ☐<br>Agree ☐<br>Strongly Agree ☐
**A69**	*FHOME 5*	I was worried someone would	Strongly Disagree ☐

For peer review only

		see me while I was changing my menstrual products	Disagree ☐<br>Agree ☐<br>Strongly Agree ☐
**A70**	*FHOME 6*	I was worried someone would harm me while I was changing my menstrual products	Strongly Disagree ☐<br>Disagree ☐<br>Agree ☐<br>Strongly Agree ☐
**A71**	*FHOME 7*	I was worried something else would harm me while I was changing my menstrual products	Strongly Disagree ☐<br>Disagree ☐<br>Agree ☐<br>Strongly Agree ☐
**A72**	*FHOME 8*	Do you have access to a clean water source at your home	Yes ☐ No ☐<br>**If "No", skip next question**
**A73**	*FHOME 9*	Where is your clean water source located?	Please tick all that apply:<br>Inside the toilet ☐<br>Outside the toilet but inside the home ☐<br>Outside the house but inside the yard of my home ☐<br>In a public space ☐
**A74**	*FHOME 10*	Do you have access to a toilet at home	Yes ☐ No ☐<br>**If "No", skip next question**
**A75**	*FHOME 11*	Where is your toilet located?	Inside the house ☐<br>Outside the house but inside the yard ☐<br>In a public space ☐
**A76**	*FHOM12*	Does your toilet have a door and a working lock on the inside (for privacy)?	Yes ☐ No ☐

PART IX: FACTORS AT SCHOOL/WORK

The following questions are about how you manage your period at school/work

For peer review only

At school/work during my last period...			
**A77**	*FSW1*	I was able to change my menstrual products when I wanted to	Strongly Disagree ☐<br>Disagree ☐<br>Agree ☐<br>Strongly Agree ☐<br>Not Applicable ☐
**A78**	*FSW2*	I was satisfied with the place I used to change my menstrual products	Strongly Disagree ☐<br>Disagree ☐<br>Agree ☐<br>Strongly Agree ☐<br>Not Applicable ☐
**A79**	*FSW3*	I had a clean place to change my menstrual materials	Strongly Disagree ☐<br>Disagree ☐<br>Agree ☐<br>Strongly Agree ☐<br>Not Applicable ☐
**A80**	*FSW4*	I was worried I would not be able to change my menstrual material when I needed to	Strongly Disagree ☐<br>Disagree ☐<br>Agree ☐<br>Strongly Agree ☐<br>Not Applicable ☐
**A81**	*FSW5*	I was worried someone would see me while I was changing my menstrual products	Strongly Disagree ☐<br>Disagree ☐<br>Agree ☐<br>Strongly Agree ☐<br>Not Applicable ☐
**A82**	*FSW6*	I was worried someone would	Strongly Disagree ☐

For peer review only

		harm me while I was changing my menstrual products	Disagree ☐<br>Agree ☐<br>Strongly Agree ☐<br>Not Applicable ☐
**A83**	*FSW7*	I was worried something else would harm me while I was changing my menstrual products	Strongly Disagree ☐<br>Disagree ☐<br>Agree ☐<br>Strongly Agree ☐<br>Not Applicable ☐
**A84**	*FSW8*	Do you have access to a toilet at school/work?	Yes ☐ No ☐<br>Not Applicable ☐<br>**If "No" or "Not Applicable", skip next 2 questions**
**A85**	*FSW9*	Where is your toilet located?	Inside the building ☐<br>Outside the building but inside the school/work yard ☐<br>In a public area, outside the school/work yard ☐
**A88**	*FSW12*	Does your toilet have a door and a working lock on the inside (for privacy)?	Yes ☐ No ☐
**A86**	*FSW10*	Do you have access to water at school/work?	Yes ☐ No ☐<br>Not Applicable ☐<br>**If "No" or "Not Applicable", skip next question**
**A87**	*FSW11*	Where is your water source located?	Please tick all that apply:<br>Inside the toilet ☐<br>Outside the toilet but inside the school/work building ☐<br>Outside the building but inside the school/work yard ☐<br>In a public area, outside the school/work yard ☐

THANK YOU FOR YOUR TIME AND YOUR ANSWERS!

For peer review only

For peer review only

Reporting checklist for cohort study.

Based on the STROBE cohort guidelines.

Instructions to authors

Complete this checklist by entering the page numbers from your manuscript where readers will find each of the items listed below.

Your article may not currently address all the items on the checklist. Please modify your text to include the missing information. If you are certain that an item does not apply, please write "n/a" and provide a short explanation.

Upload your completed checklist as an extra file when you submit to a journal.

In your methods section, say that you used the STROBE cohortreporting guidelines, and cite them as:

von Elm E, Altman DG, Egger M, Pocock SJ, Gotzsche PC, Vandenbroucke JP. The Strengthening the Reporting of Observational Studies in Epidemiology (STROBE) Statement: guidelines for reporting observational studies.

		Reporting Item	Page Number
**Title and abstract**			
Title	#1a	Indicate the study's design with a commonly used term in the title or the abstract	3
Abstract	#1b	Provide in the abstract an informative and balanced summary of what was done and what was found	3-4
**Introduction**			
Background / rationale	#2	Explain the scientific background and rationale for the investigation being reported	6-7
Objectives	#3	State specific objectives, including any prespecified hypotheses	7
**Methods**			
Study design	#4	Present key elements of study design early in the paper	7

Setting	#5	Describe the setting, locations, and relevant dates, including periods of recruitment, exposure, follow-up, and data collection	7-8
Eligibility criteria	#6a	Give the eligibility criteria, and the sources and methods of selection of participants. Describe methods of follow-up.	8-9
Eligibility criteria	#6b	For matched studies, give matching criteria and number of exposed and unexposed	N/A Not a matched study
Variables	#7	Clearly define all outcomes, exposures, predictors, potential confounders, and effect modifiers. Give diagnostic criteria, if applicable	10-12
Data sources / measurement	#8	For each variable of interest give sources of data and details of methods of assessment (measurement). Describe comparability of assessment methods if there is more than one group. Give information separately for for exposed and unexposed groups if applicable.	10-12
Bias	#9	Describe any efforts to address potential sources of bias	10
Study size	#10	Explain how the study size was arrived at	9
Quantitative variables	#11	Explain how quantitative variables were handled in the analyses. If applicable, describe which groupings were chosen, and why	9
Statistical methods	#12a	Describe all statistical methods, including those used to control for confounding	
9-10			
Statistical methods	#12b	Describe any methods used to examine subgroups and interactions	9-10
Statistical methods	#12c	Explain how missing data were addressed	9-10
Statistical methods	#12d	If applicable, explain how loss to follow-up was addressed	9-10
Statistical methods	#12e	Describe any sensitivity analyses	

For peer review only

N/A

Results

Participants	#13a	Report numbers of individuals at each stage of study—eg numbers potentially eligible, examined for eligibility, confirmed eligible, included in the study, completing follow-up, and analysed. Give information separately for for exposed and unexposed groups if applicable.	14
Participants	#13b	Give reasons for non-participation at each stage	14
Participants	#13c	Consider use of a flow diagram	
14			
Descriptive data	#14a	Give characteristics of study participants (eg demographic, clinical, social) and information on exposures and potential confounders. Give information separately for exposed and unexposed groups if applicable.	15
Descriptive data	#14b	Indicate number of participants with missing data for each variable of interest	
14			
Descriptive data	#14c	Summarise follow-up time (eg, average and total amount)	
14-15			
Outcome data	#15	Report numbers of outcome events or summary measures over time. Give information separately for exposed and unexposed groups if applicable.	
16-17			
Main results	#16a	Give unadjusted estimates and, if applicable, confounder-adjusted estimates and their precision (eg, 95% confidence interval). Make clear which confounders were adjusted for and why they were included	17
Main results	#16b	Report category boundaries when continuous variables were categorized	16-17
Main results	#16c	If relevant, consider translating estimates of relative risk into absolute risk for a meaningful time period	

For peer review only

N/A			
Other analyses	#17	Report other analyses done—eg analyses of subgroups and interactions, and sensitivity analyses	17-22
**Discussion**			
Key results	#18	Summarise key results with reference to study objectives	23
Limitations	#19	Discuss limitations of the study, taking into account sources of potential bias or imprecision. Discuss both direction and magnitude of any potential bias.	24
Interpretation	#20	Give a cautious overall interpretation considering objectives, limitations, multiplicity of analyses, results from similar studies, and other relevant evidence.	24
Generalisability	#21	Discuss the generalisability (external validity) of the study results	24-25
**Other Information**			
Funding	#22	Give the source of funding and the role of the funders for the present study and, if applicable, for the original study on which the present article is based	28

The STROBE checklist is distributed under the terms of the Creative Commons Attribution License CC-BY. This checklist was completed on 23. August 2022 using https://www.goodreports.org/, a tool made by the EQUATOR Network in collaboration with Penelope.ai

For peer review only

S1 Table. Enhancing transparency in reporting the synthesis of qualitative research: ENTREQ Checklist (Tong, *et al.*, 2012)

Item No.	Guide and Description	Report Location
1. Aim	To investigate the effectiveness of a comprehensive MH intervention integrated within a community-based sexual and reproductive health (SRH) service on MH knowledge, perceptions, and practices among young women in Zimbabwe	Title and page 7
2. Synthesis methodology	We conducted a prospective cohort study from December 2019 to August 2021, using quantitative and qualitive methods to assess MH outcomes. Participants in the cohort study were followed up every three months for a year.<br>Following the 12-month visits, after exposure to the intervention, four focus group discussions (FGDs) were conducted with a total of 48 cohort participants in two of the intervention clusters. FGD participants were purposively selected to include a range of ages and MH product choices. 12-15 young women participated in each of four FGDs. In each of the two clusters, one FGD was conducted with 16-19-year-olds, and one was conducted with 20–24-year-olds.<br>Semi-structured topic guides were informed by findings from the MH pilot study and the preliminary analysis of the quantitative data. Analysis was guided by Braun and Clarke's approach to thematic analysis, using a mix of deductive and inductive themes.	Methods (pages 7-13)
3. Approach to searching	The research was pre-planned. Semi-structured topic guides were informed by findings from the MH pilot study and the preliminary analysis of the quantitative data. The topic guide explored participants' perspectives on the MH intervention, what they had learned and/or gained from the intervention, which MH products participants chose, if and how their experiences of managing menstruation had changed over time, how they felt about MH in general, and how COVID-19 had affected their lives and menstrual practices.	Page 13
4. Inclusion criteria	Participants were excluded if 1) they were currently pregnant or intended to get pregnant within the next year; 2) they experienced irregular or absent bleeding within the last 3 months; or 3) had any condition that precluded informed consent or made study participation unsafe.	Page 9
5. Data sources	A quantitative questionnaire informed by findings from the pilot intervention (20) and Hennegan's Menstrual Practice Needs Scale (MPNS-36) (19), was used to collect data on: 1) sociodemographic information; 2)	Page 10 - 14

For peer review only

	menstruation and puberty knowledge; 3) menstrual history; 4) pain management; 5) practices and perceptions during menstruation; 6) washing practices; and 7) attitudes about menstruation.<br><br>Semi-structured topic guides were informed by findings from the MH pilot study and the preliminary analysis of the quantitative data. The topic guide explored participants' perspectives on the MH intervention, what they had learned and/or gained from the intervention, which MH products participants chose, if and how their experiences of managing menstruation had changed over time, how they felt about MH in general, and how COVID-19 had affected their lives and menstrual practices.	
6. Electronic Search strategy	Not a systematic review	N/A
7. Study screening methods	Not a systematic review	N/A
8. Study characteristics	Not a systematic review	N/A
9. Study selection results	Not a systematic review	N/A
10. Rationale for appraisal	Not a systematic review	N/A
11. Appraisal items	Not a systematic review	N/A
12. Appraisal process	Not a systematic review	N/A
13. Appraisal results	Not a systematic review	N/A
14. Data extraction	Not a systematic review	N/A
15. Software	NVivo 12	Page 14
16. Number of reviewers	First author (MT) and senior social scientist (CMY)	Page 14
17. Coding	Initial deductive codes were based on the preliminary findings from the quantitative questionnaire. Additional inductive codes were generated through transcript familiarisation, initial manual coding, and analytical discussions between MT and CMY, and adapted through the coding process. Deductive and inductive codes were merged to develop a coding framework, which was used to code all transcripts.	Page 14

For peer review only

18. Study comparison	Not a systematic review	N/A
19. Derivation of themes	Analysis was guided by Braun and Clarke's approach to thematic analysis, using a mix of deductive and inductive themes	Page 14
20. Quotations	Quotes provided in text and table	Results- *Page 19 -23*
21. Synthesis output	Present rich, compelling and useful results that go beyond a summary of the primary studies (e.g. new interpretation, models of evidence, conceptual models, analytical framework, development of a new theory or construct)	Discussion - *highlights the generation and development of theory* Pages 23 - 26

For peer review only
